# Design, Synthesis and Biological Evaluation of a New Series of 1-Aryl-3-{4-[(pyridin-2-ylmethyl)thio]phenyl}urea Derivatives as Antiproliferative Agents

**DOI:** 10.3390/molecules24112108

**Published:** 2019-06-04

**Authors:** Chuanming Zhang, Xiaoyu Tan, Jian Feng, Ning Ding, Yongpeng Li, Zhe Jin, Qingguo Meng, Xiaoping Liu, Chun Hu

**Affiliations:** 1 Key Laboratory of Structure-Based Drug Design & Discovery, Ministry of Education, Shenyang Pharmaceutical University, Shenyang 110016, China; chuanming_zhang@yeah.net (C.Z.); tanxiaoyuaikaoyan@163.com (X.T.); mr.fengjian@foxmail.com (J.F.); dingning2216@163.com (N.D.); yongpengli1993@163.com (Y.L.); jinzheln@163.com (Z.J.); 2 Department of Pharmacy, Yantai University, Yantai 264005, China; qinggmeng@163.com

**Keywords:** antiproliferative agent, urea, synthesis, antiproliferative activity, apoptosis

## Abstract

To discover new antiproliferative agents with high efficacy and selectivity, a new series of 1-aryl-3-{4-[(pyridin-2-ylmethyl)thio]phenyl}urea derivatives (**7a**–**7t**) were designed, synthesized and evaluated for their antiproliferative activity against A549, HCT-116 and PC-3 cancer cell lines in vitro. Most of the target compounds demonstrated significant antiproliferative effects on all the selective cancer cell lines. Among them, the target compound, 1-[4-chloro-3-(trifluoromethyl)phenyl]-3-{4-{{[3-methyl-4-(2,2,2-trifluoroethoxy)pyridin-2-yl]methyl}thio}phenyl}urea (**7i**) was identified to be the most active one against three cell lines, which was more potent than the positive control with an IC_50_ value of 1.53 ± 0.46, 1.11 ± 0.34 and 1.98 ± 1.27 μM, respectively. Further cellular mechanism studies confirmed that compound **7i** could induce the apoptosis of A549 cells in a concentration-dependent manner and elucidated compound **7i** arrests cell cycle at G1 phase by flow cytometry analysis. Herein, the studies suggested that the 1-aryl-3-{4-[(pyridin-2-ylmethyl)thio]phenyl}urea skeleton might be regarded as new chemotypes for designing effective antiproliferative agents.

## 1. Introduction

Cancer is a major public health problem in developed countries and will become the most serious life-threatening disease worldwide in the near future [1]. Some advances in cancer treatment by molecule-targeted drugs, such as imatinib, gefitinib, and trastuzumab, were expected to improve cancer cure rates and also to reduce severe adverse reactions because of the high specificity of the targeted molecules, which are expressed and have critical roles in cancer cells, but not in normal cells. However, the clinical effect was found to be limited and did not last for a long time period because of the acquired resistance of the tumor cells. Furthermore, these molecules often cause on-target and/or off-target severe toxicity [2]. Therefore, the development of more target-specific therapy, with minimum toxicity, is warranted to extend disease-free survival and improve the quality of life of cancer patients.

**Figure 1:**
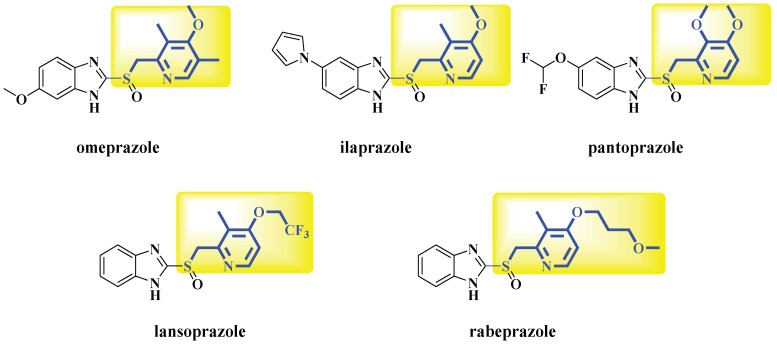
Chemical structures of proton pump inhibitors (PPIs).

In recent years, proton pump inhibitors (PPIs) as potential anticancer agents were intensively studied in cancer treatment. Lugini et al. compared the anti-tumor efficacy of different PPIs, including omeprazole, esomeprazole, lansoprazole, rabeprazole and pantoprazole in vitro and in vivo. The result indicated that all the PPIs have shown different degrees of antitumor efficacy and lansoprazole showed a higher anti-tumor effect when compared to the other PPIs. [3]. Recently, the research by Zeng and Zheng et al. indicated that T-cell originated protein kinase (TOPK) activities were inhibited by pantoprazole and ilaprazole with high affinity and selectivity [4,5]. TOPK (also known as PBK or PDZ-binding kinase) was first reported by Abe et al. in 2000 [6], and it is a Ser/Thr protein kinase overexpressed in hematologic tumors, breast cancer, melanoma, colorectal cancer, prostate cancer, cervical cancer, bladder cancer and lung cancer [7,8,9,10,11,12,13,14]. The results of their studies demonstrated that pantoprazole can suppress the growth of colorectal cancer cells as a TOPK inhibitor both in vitro and in vivo, and also showed that the TOPK activities were inhibited by ilaprazole in HCT-116, ES-2, A549, SW1990 cancer cells in vitro [4,5]. As shown in Figure 1, all of the PPIs molecules contain thiomethylpyridine fragments. It can be predicted that these fragments should play an important role in the antiproliferative activity of proton pump inhibitors.

**Figure 2:**
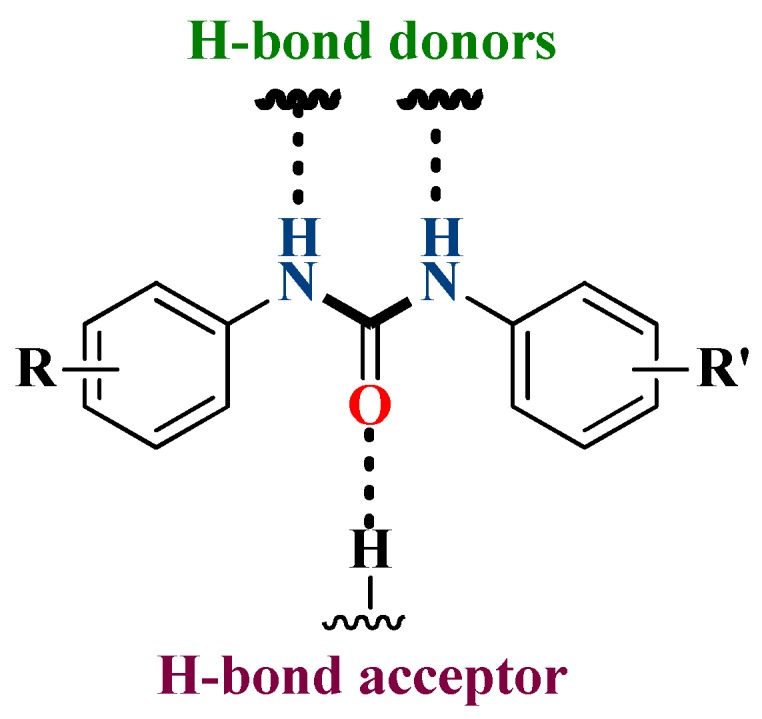
H-bond acceptor and donors within the diaryl urea scaffold.

**Figure 3:**
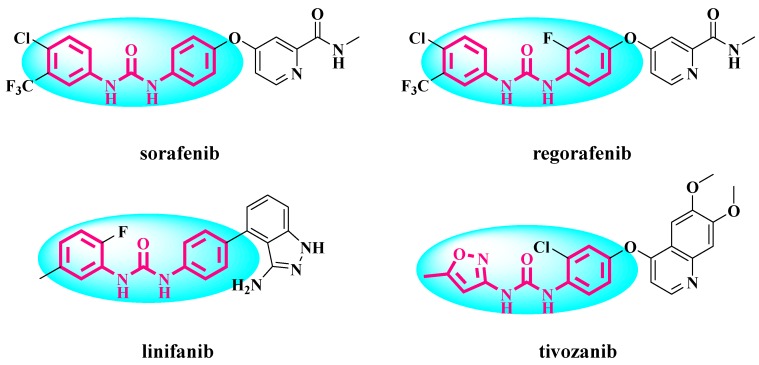
Some anticancer drugs of the diaryl urea moiety.

As known, the diaryl urea is a fragment of great importance in medicinal chemistry and can be used for the synthesis of numerous heterocyclic compounds with diversified biological activities, including antithrombotic [15], antimalarial [16], antibacterial [17,18] and anti-inflammatory [19] properties, and it is characterized by its ability to form hydrogen bond interactions with drug targets [20,21,22]. The carbonyl oxygen atom acts as a proton acceptor while the two amide nitrogen atoms are proton donors (Figure 2). This unique type of structure endows urea derivatives with the ability to bind a variety of enzymes and receptors in the biological systems. Remarkably, the diaryl urea moiety is widely used in the design of anticancer drugs, such as sorafenib, regorafenib, linifanib and tivozanib (Figure 3).

**Figure 4:**
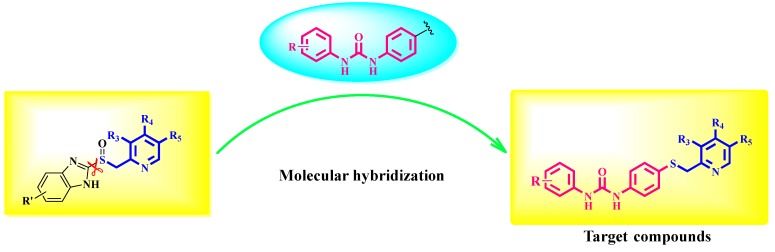
Rational design of the target compounds based on molecular hybridization strategy.

Molecular hybridization strategy is a useful concept in drug design and development based on the combination of pharmacophoric moieties of different bioactive substances to produce a new structure, the affinity and efficacy would be improved, when compared to the parent drugs [23]. These above interesting findings and our continuous quest to identify more potent antiproliferative agents led to the molecular hybridization of diaryl urea and thiomethylpyridine to integrate them in one molecular platform to generate a new hybrid, as shown in Figure 4, and expected that taking this way could get the antiproliferative agents with highly inhibitory activity.

## 2. Results and Discussion

### 2.1. Chemistry

**Scheme 1:**
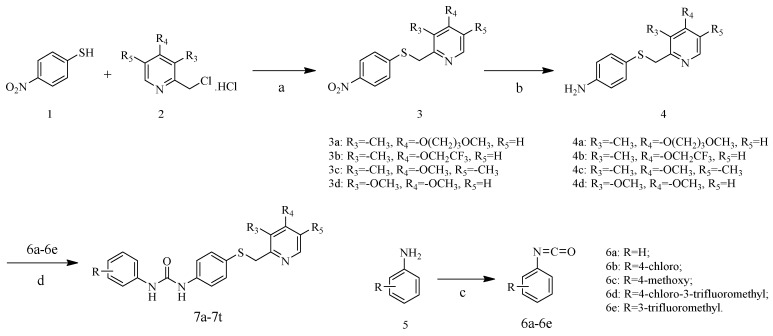
Synthetic route of the target compounds **7a**–**7t.** Reagents and conditions: (a) NaOH (aq. 2M), EtOH, r.t.; (b) H_2_, 1 atm, 10% Pd-C, MeOH, r.t.; (c) BTC, Et_3_N, CH_2_Cl_2_, r.t.; (d) intermediate **4**, aryl isocyanates **6a**–**6e**, CH_2_Cl_2_, r.t.

The general synthetic route is illustrated in Scheme 1. The reaction of the commercially available 4-nitrobenzenethiol (**1**) with 2-(chloromethyl)pyridine derivatives (**2**) in ethanol at r.t. (room temperature) obtained compounds **3a**–**3d [24]**, which converted to key intermediates **4a**–**4d** via Pd-C catalytic hydrogenation reduction [25]. The aryl isocyanates **6a**–**6e** were prepared by reaction between aromatic amines and bis(trichloromethyl)carbonate (BTC) [26]. Finally, treatment of **4a**–**4d** with aryl isocyanates **6a**–**6e** in methylene dichloride yielded 1-aryl-3-{4-[(pyridin-2-ylmethyl)thio]phenyl}urea derivatives (**7a**–**7t**) as the target compounds [26]. The structures of the target compounds were characterized by infraredspectra (IR), proton nuclear magnetic resonance spectra (^1^H-NMR), carbon nuclear magnetic resonance spectra (^1^^3^C-NMR), electrospray ionization mass spectra (ESI-MS) and high-resolution mass spectra (HRMS).

### 2.2. Biological Evaluation

#### 2.2.1. Antiproliferative Activity

Using sorafenib as a positive control, all of the target compounds were evaluated for the antiproliferative activity in vitro against cancer cell lines, including A549 (lung cancer), HCT-116 (colorectal cancer), and PC-3 (prostate cancer) cell lines by MTT assay. The antiproliferative assay results evaluated as IC_50_ value (Table 1) and demonstrated that several target compounds have shown moderate to excellent potency against A549, HCT-116, and PC-3 cancer cell lines. Among the target compounds**7i** showed the more potent inhibitory effect against three cancer cell lines than positive control with IC_50_ values of 1.53 ± 0.46, 1.11 ± 0.34 and 1.98 ± 1.27 μM, respectively.

**Figure 5:**
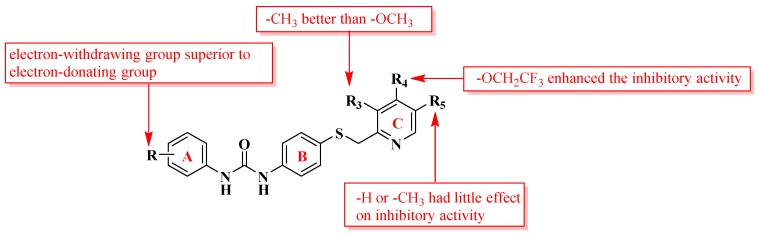
SARs summary for the target compounds.

All the target compounds could be divided into four classes according to different substituents on the pyridine ring (Figure 5). The analyses of the structure-activity relationships (SARs) were summarized as follows: (1) The results of cytostatic activity assay showed that the substitutions of the 4 (R_4_) and 5 (R_5_) positions of the C ring had a weak effect on the inhibitory activity. However, if the 3-position (R_3_) hydrogen atom of the C ring was substituted by a methoxy group, the inhibitory activity was significantly decreased. At the same time, it could be seen that the inhibitory activity was better than other classes when the 4-position (R_4_) of the C ring was occupied by the trifluoroethoxy group. (2) The substituents on the A ring had a significant effect on the inhibitory activity of each class. When the substituents on C ring were the same, if there was no substituent on A ring, the inhibitory activity was worst in each class, such as compound **7a**, **7f**, **7k** and **7p**. Moreover, when the substituents in A ring were electron-withdrawing groups, such as 4-Cl or 3-CF_3_, the inhibitory activity was better than that substitution of the electron-donating groups, such as 4-OCH_3_ in each class. Furthermore, when the two electron-withdrawing groups coexist on the A ring, the target compounds displayed the strongest inhibitory activity, such as compound **7d**, **7i, 7n** and **7s**.

#### 2.2.2. Cell Apoptosis Assay 

**Figure 6:**
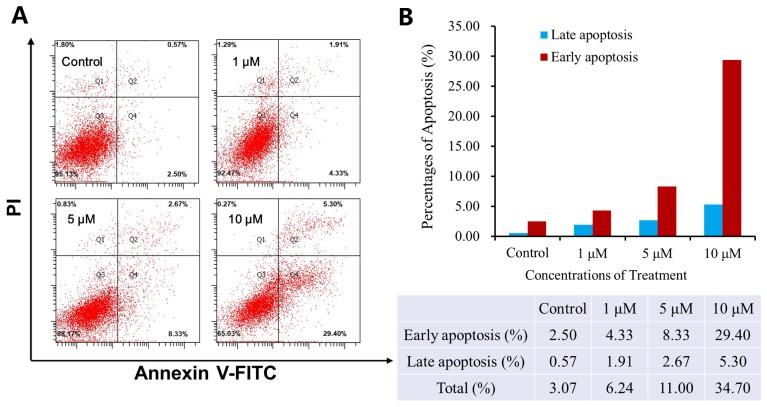
Compound **7i** induced apoptosis of A549 cells. (**A**) Apoptosis effect on A549 cell line induced by compound **7i** for 24 h using Annexin V-FITC/PI double staining and flow-cytometry calculation. The lower left quadrant represents live cells, the lower right is for early/primary apoptotic cells, upper right is for late/secondary apoptotic cells, and the upper left represents cells damaged during the procedure; (**B**) Quantitative analysis of apoptotic cells. The experiments were performed three times, and a representative experiment is shown.

The acceptable antiproliferative activity of compound **7i** promoted us to investigate its effect on cell apoptosis. To explore the effect of compound **7i** on cell apoptosis, the apoptotic analysis was performed with Annexin V-FITC/PI double staining and analyzed with flow-cytometry calculation. Treatment of A549 cells with compound **7i** resulted in a concentration-dependent apoptosis increase, as shown in Figure 6. Specifically, the percentage of early/primary apoptotic cells was about 4.33% for the low concentration (1 μM) of compound **7i**. When treated with high concentration (10 μM) of compound **7i**, around 29.47% of early/primary apoptosis rate was observed. While the late apoptosis rate of A549 cells was not changed significantly with increasing concentrations.

#### 2.2.3. Cell Cycle Analysis

**Figure 7:**
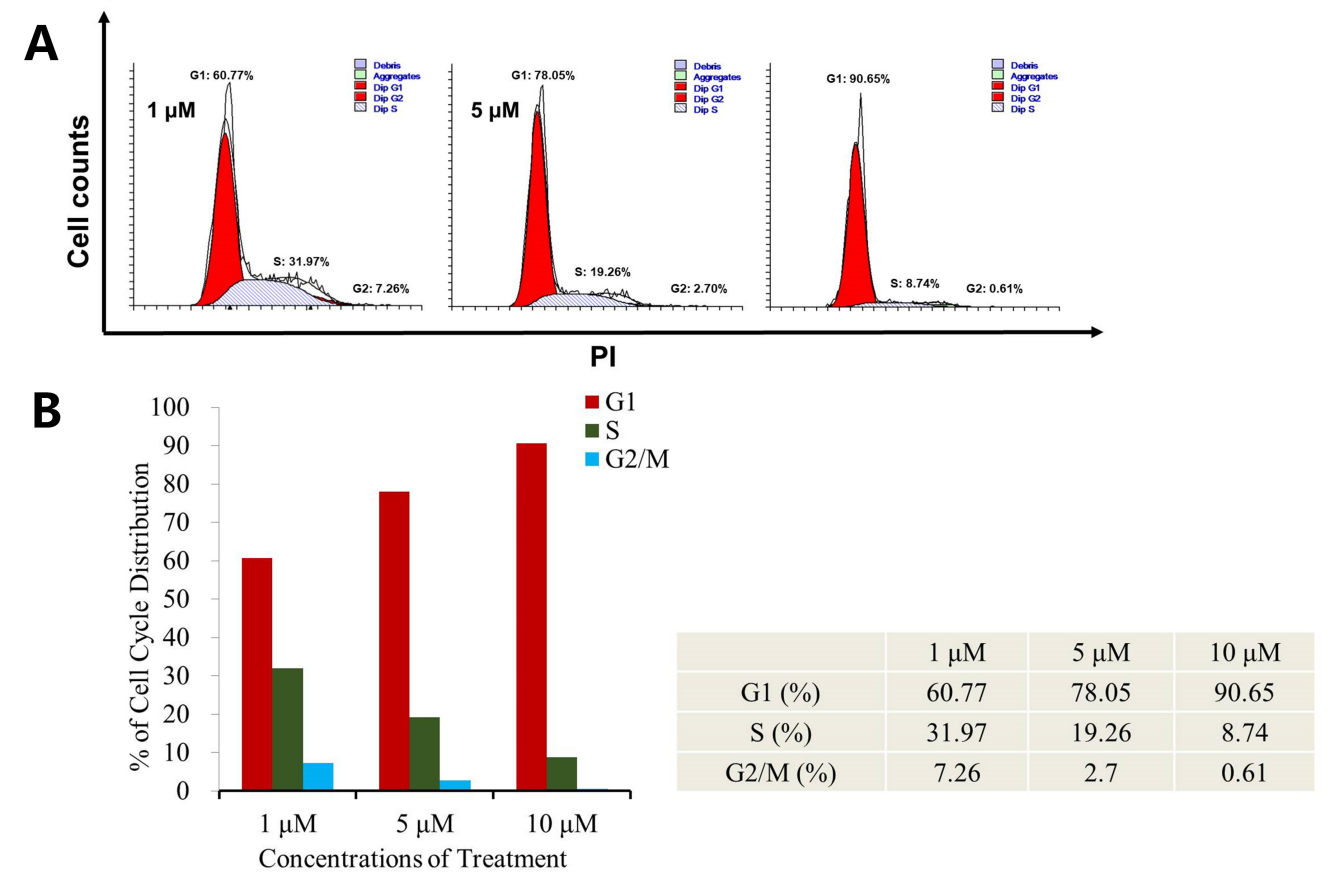
Effects of compound **7i** on A549 cell cycle progress for 24 h. (**A**) Treatment of A549 cells with compound **7i** at different concentrations (1 μM, 5 μM, 10 μM) for 24 h. (**B**) Quantitative analysis of cell cycle. The experiments were performed three times, and a representative experiment is shown.

The effect of compound **7i** on the cell cycle was also evaluated. After treatment of A549 cells with compound **7i** for 24 h at indicated concentrations (1, 5, 10 μM), the percentage of cells in G1 phase were 60.77%, 78.05% and 90.65%, respectively (Figure 7), suggesting that compound **7i** caused an obvious G1 arrest in a concentration-dependent manner with a concomitant decrease in terms of the number of cells in other phases of the cell cycle. 

## 3. Materials and Methods

### 3.1. Synthesis

All reagents were obtained from commercial suppliers and used without further purification. Reaction progress was monitored by thin layer chromatography (TLC) on silica gel plates. The spots were visualized by ultraviolet (UV) light (254 nm). The column chromatography was performed using 200−300 mesh silica gel (Qingdao PUKE, Qingdao, China). Melting points were obtained by X-5 micro-melting point apparatus (Beijing Zhongyi Boteng Technology Co., Ltd., Beijing, China) and were uncorrected. ^1^H-NMR and ^1^^3^C-NMR spectra were recorded on Bruker NMR spectrophotometers (Karlsruhe, Germany) using DMSO-*d*_6_ as the solvent and TMS as the internal standard. Mass spectra were measured with an electrospray (ESI-MS) on a Waters spectrometer (Waters Corporation, Milford, MA, USA). High resolution mass spectrometry (HRMS) analyses were performed on an Agilent Technologies 6530 Accurate-Mass Q-TOF Mass Spectrometer (Santa Clara, CA, USA). The purities were determined by high-performance liquid chromatography (HPLC) using an Agilent 1100 series HPLC (Santa Clara, CA, USA).

The original figures of ^1^H-NMR, ^13^C-NMR, MS and HRMS of all the target compounds as the Supplementary Materials are available online.

#### 3.1.1. General Procedure for the Preparation of 2-{[(4-nitrophenyl)thio]methyl}pyridine Derivatives (**3a**–**3d**)

4-Nitrobenzenethiol **1** (1.55 g, 0.01 mol), and 2-(chloromethyl)pyridine hydrochloride derivatives **2** (0.01 mol) were dissolved in EtOH (100 mL), then aqueous NaOH (2M) was added dropwise. After the addition completed, the solution was stirred for 8 h at room temperature. Upon completion, the excess ethanol was evaporated to give the residue. A large number of white solids have been precipitated when 200 mL of water was added. The Precipitate was filtered off and washed with water to obtain the intermediates (**3a**–**3d**), which was used for next step without further purification. 

*4-(3-Methoxypropoxy)-3-methyl-2-{[(4-nitrophenyl)thio]methyl}pyridine* (**3a**) by using compound **1** (1.55 g, 0.01 mol) and 2-(chloromethyl)-4-(3-methoxypropoxy)-3-methylpyridine hydrochloride (2.66 g, 0.01 mol), obtained a yellow solid (3.12 g) in 89.7% yield. ESI-MS (*m*/*z*): 349.3 ([M + H]^+^).

*3-Methyl-2-{[(4-nitrophenyl)thio]methyl}-4-(2,2,2-trifluoroethoxy)pyridine* (**3b**) by using compound **1** (1.55 g, 0.01 mol) and 2-(chloromethyl)-3-methyl-4-(2,2,2-trifluoroethoxy)pyridine hydrochloride (2.76 g, 0.01 mol), obtained a yellow solid (3.26 g) in 91.2% yield. ESI-MS (*m*/*z*): 359.1 ([M + H]^+^).

*4-Methoxy-3,5-dimethyl-2-{[(4-nitrophenyl)thio]methyl}pyridine* (**3c**) by using compound **1** (1.55 g, 0.01 mol) and 2-(chloromethyl)-4-methoxy-3,5-dimethylpyridine hydrochloride (2.22 g, 0.01 mol), obtained a yellow solid (2.69 g) in 88.5% yield. ESI-MS (*m*/*z*): 305.0 ([M + H]^+^).

*3,4-Dimethoxy-2-{[(4-nitrophenyl)thio]methyl}pyridine* (**3d**) by using compound **1** (1.55 g, 0.01 mol) and 2-(chloromethyl)-3,4-dimethoxypyridine hydrochloride (2.24 g, 0.01 mol), obtained a yellow solid (2.86 g) in 93.5% yield. ESI-MS (*m*/*z*): 307.4 ([M + H]^+^).

#### 3.1.2. General Procedure for the Preparation of 4-{[(pyridin-2-yl)methyl]thio}aniline Derivatives (**4a**–**4d**)

A mixture of **3a**–**3d** (5 mmol) and 0.1 g of preequilibrated 10% palladium/carbon in MeOH (50 mL) was hydrogenated at room temperature and atmospheric pressure. The reaction was completely monitored by TLC. When the reaction has completed, the mixture was filtered, and the filtrate was evaporated to yield intermediate (**4a**–**4d**) as yellowish oil, which was used for the next step without further purification.

*4-{{[4-(3-Methoxypropoxy)-3-methylpyridin-2-yl]methyl}thio}aniline* (**4a**) by using compound **3a** (1.74 g, 5 mmol), H_2_ and 10% Pd-C, obtained a yellowish oil (1.52 g) in 95.4% yield. ESI-MS (*m*/*z*): 319.3 ([M + H]^+^).

*4-{{[3-Methyl-4-(2,2,2-trifluoroethoxy)pyridin-2-yl]methyl}thio}aniline* (**4b**) by using compound **3b** (1.79 g, 5 mmol), H_2_ and 10% Pd-C, obtained a yellowish oil (1.58 g) in 96.1% yield. ESI-MS (*m*/*z*): 329.6 ([M + H]^+^).

*4-{[(4-Methoxy-3,5-dimethylpyridin-2-yl)methyl]thio}aniline* (**4c**) by using compound **3c** (1.52 g, 5 mmol), H_2_ and 10% Pd-C, obtained a yellowish oil (1.30 g) in 94.8% yield. ESI-MS (*m*/*z*): 275.2 ([M + H]^+^).

*4-{[(3,4-Dimethoxypyridin-2-yl)methyl]thio}aniline* (**4d**) by using compound **3d** (1.53 g, 5 mmol), H_2_ and 10% Pd-C, obtained a yellowish oil (1.30 g) in 94.2% yield. ESI-MS (*m*/*z*): 276.1 ([M + H]^+^).

#### 3.1.3. General Procedure for the Preparation of the Target Compounds (**7a**–**7t**)

To a solution of BTC (1 mmol) in CH_2_Cl_2_ (20 mL) was added dropwise to primary aromatic amine **5** (1 mmol) in CH_2_Cl_2_ (20 mL) followed by the dropwise addition of triethylamine (1 mL) in CH_2_Cl_2_ (10 mL). The solvent was evaporated. The resulting residue was dissolved in CH_2_Cl_2_ (20 mL), and intermediates (**4a**–**4d**) (1 mmol) in CH_2_Cl_2_ (10 mL) was added dropwise. The mixture was stirred for about 3 h, monitored by TLC. After the reaction completed, the solvent was washed with water and brine, then dried over anhydrous magnesium sulfate. The mixture was filtered, the filtrate was evaporated and purified by silica gel chromatography (CH_2_Cl_2_/MeOH = 60/1, *v*/*v*) to obtain target compounds.

*1-{4-{{[4-(3-Methoxypropoxy)-3-methylpyridin-2-yl]methyl}thio}phenyl}-3-phenylurea* (**7a**)


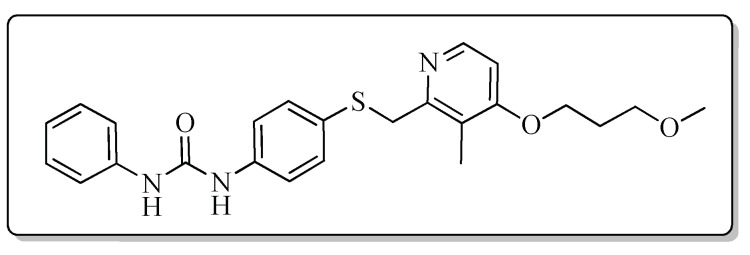


Compound **7a** was prepared according to the general procedure by using compound **4a** (0.32 g, 1 mmol) and aniline (0.10 g, 1 mmol), obtained a white solid (0.20 g) in 45.1% yield. m.p. 105.2–106.8 °C. IR (KBr, cm^−1^): υ 3421.1, 2922.7, 2852.4, 1596.1, 1545.1, 1492.4, 1460.8, 1440.3, 1398.2, 1385.2, 1309.3, 1231.3, 1174.2, 1092.1, 1006.7, 894.6, 832.0, 799.2, 751.9, 693.4, 617.3, 507.5. ^1^H-NMR (400 MHz, DMSO-*d*_6_) δ 8.74 (s, 1H), 8.69 (s, 1H), 8.17 (d, *J* = 5.6 Hz, 1H), 7.46 (s, 1H), 7.44 (s, 1H), 7.41 (s, 1H), 7.39 (s, 1H), 7.32 (s, 1H), 7.31–7.29 (m, 1H), 7.28 (s, 1H), 7.26 (s, 1H), 6.97 (t, *J* = 7.3 Hz, 1H), 6.90 (d, *J* = 5.7 Hz, 1H), 4.22 (s, 2H), 4.09 (t, *J* = 6.2 Hz, 2H), 3.48 (t, *J* = 6.2 Hz, 2H), 3.25 (s, 3H), 2.12 (s, 3H), 2.02–1.94 (m, 2H). ^13^C-NMR (101 MHz, DMSO-*d*_6_) δ 163.17, 156.31, 152.87, 147.91, 140.07, 131.77, 129.23, 128.07, 122.34, 120.30, 119.09, 118.69, 106.49, 68.79, 65.48, 58.44, 31.14, 29.16, 10.88. ESI-MS (*m*/*z*): 438.4 ([M + H]^+^), 460.2 ([M + Na]^+^). HRMS (ESI) (*m*/*z*): Calcd. for C_24_H_27_N_3_O_3_S, 438.1846 ([M + H]^+^), found: 438.1856 ([M + H]^+^). Purity (HPLC): 99.27%.

*1-(4-Chlorophenyl)-3-{4-{{[4-(3-methoxypropoxy)-3-methylpyridin-2-yl]methyl}thio}phenyl}urea* (**7b**)


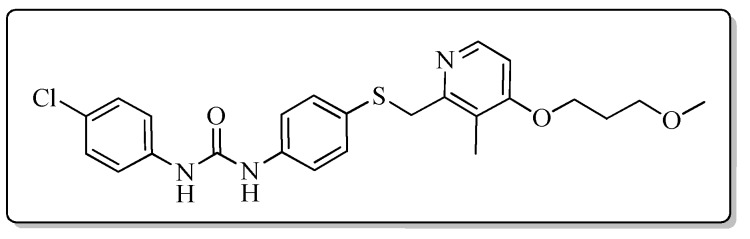


Compound **7b** was prepared according to the general procedure by using compound **4a** (0.32 g, 1 mmol) and 4-chloroaniline (0.13 g, 1 mmol), obtained a white solid (0.36 g) in 75.3% yield. m.p. 191.7–192.5 °C. IR (KBr, cm^−1^): υ 3428.1, 2923.1, 2852.9, 1631.8, 1490.8, 1398.9, 1384.8, 1298.9, 1273.5, 1237.0, 1174.2, 1121.4, 1086.2, 1008.0, 881.2, 832.1, 702.8, 619.7, 506.0. ^1^H-NMR (400 MHz, DMSO-*d*_6_) δ 8.83 (s, 1H), 8.77 (s, 1H), 8.17 (d, *J* = 5.6 Hz, 1H), 7.49 (d, *J* = 2.2 Hz, 1H), 7.47 (d, *J* = 2.1 Hz, 1H), 7.40 (d, *J* = 2.0 Hz, 1H), 7.39 (d, *J* = 2.1 Hz, 1H), 7.34 (s, 1H), 7.33 (s, 1H), 7.32 (d, *J* = 2.1 Hz, 1H), 7.31 (s, 1H), 6.90 (d, *J* = 5.7 Hz, 1H), 4.22 (s, 2H), 4.08 (t, *J* = 6.2 Hz, 2H), 3.48 (t, *J* = 6.2 Hz, 2H), 3.25 (s, 3H), 2.12 (s, 3H), 2.00–1.94 (m, 2H). ^13^C-NMR (101 MHz, DMSO-*d*_6_) δ 163.09, 156.35, 152.77, 147.99, 139.09, 138.88, 131.62, 129.07, 128.41, 125.86, 120.22, 119.24, 106.47, 68.80, 65.45, 58.43, 31.14, 29.16, 10.89. ESI-MS (*m*/*z*): 473.3 ([M + H]^+^), 495.2 ([M + Na]^+^). HRMS (ESI) (*m*/*z*): Calcd. for C_24_H_26_ClN_3_O_3_S, 472.1456 ([M + H]^+^), found: 472.1467 ([M + H]^+^). Purity (HPLC): 98.66%.

*1-(4-Methoxyphenyl)-3-{4-{{[4-(3-methoxypropoxy)-3-methylpyridin-2-yl]methyl}thio}phenyl}urea* (**7c**)


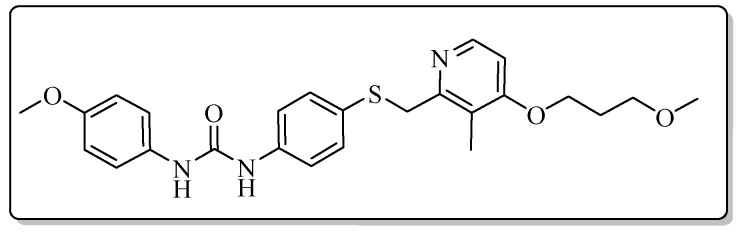


Compound **7c** was prepared according to the general procedure by using compound **4a** (0.32 g, 1 mmol) and 4-methoxyaniline (0.12 g, 1 mmol), obtained a white solid (0.22 g) in 46.3% yield. m.p. 144.8–146.6 °C. IR (KBr, cm^−1^): υ 3428.6, 2984.7, 2923.0, 2852.7, 1635.5, 1599.5, 1562.6, 1510.7, 1492.7, 1461.8, 1441.3, 1398.1, 1289.8, 1245.2, 1173.5, 1120.3, 1093.5, 1035.2, 1005.6, 800.0, 617.1, 548.4, 522.7. ^1^H-NMR (400 MHz, DMSO-*d*_6_) δ 8.64 (s, 1H), 8.47 (s, 1H), 8.16 (d, *J* = 5.7 Hz, 1H), 7.39 (d, *J* = 1.9 Hz, 1H), 7.37 (d, *J* = 2.2 Hz, 1H), 7.35 (d, *J* = 2.2 Hz, 1H), 7.33 (d, *J* = 2.3 Hz, 1H), 7.30 (d, *J* = 2.1 Hz, 1H), 7.29 (d, *J* = 2.0 Hz, 1H), 6.89 (d, *J* = 5.6 Hz, 1H), 6.87 (d, *J* = 2.2 Hz, 1H), 6.86 (d, *J* = 2.2 Hz, 1H), 4.21 (s, 2H), 4.08 (t, *J* = 6.2 Hz, 2H), 3.71 (s, 3H), 3.48 (t, *J* = 6.2 Hz, 2H), 3.25 (s, 3H), 2.12 (s, 3H), 2.00–1.94 (m, 2H). ^13^C-NMR (101 MHz, DMSO-*d*_6_) δ 163.06, 156.41, 154.97, 153.07, 148.01, 139.37, 133.09, 131.78, 127.82, 120.52, 120.22, 118.98, 114.44, 106.44, 68.79, 65.42, 58.43, 55.63, 31.14, 29.16, 10.88. ESI-MS (*m*/*z*): 468.4 ([M + H]^+^), 490.2 ([M + Na]^+^). HRMS (ESI) (*m*/*z*): Calcd. for C_25_H_29_N_3_O_4_S, 468.1952 ([M + H]^+^), found: 468.1959 ([M + H]^+^). Purity (HPLC): 98.91%.

*1-[4-Chloro-3-(trifluoromethyl)phenyl]-3-{4-{{[4-(3-methoxypropoxy)-3-methylpyridin-2-yl]methyl}thio}phenyl}urea* (**7d**)


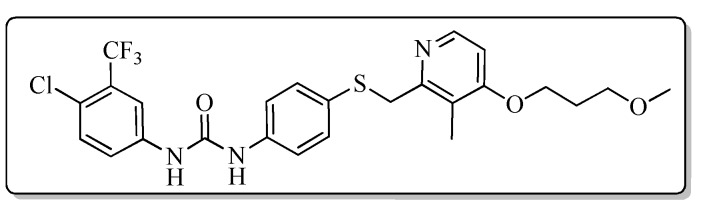


Compound **7d** was prepared according to the general procedure by using compound **4a** (0.32 g, 1 mmol) and 3-chloro-4-(trifluoromethyl)aniline (0.20 g, 1 mmol), obtained a white solid (0.24 g) in 43.8% yield. m.p. 136.0–137.2 °C. IR (KBr, cm^−1^): υ 3425.2, 2922.0, 2852.7, 1590.1, 1546.1, 1482.2, 1463.1, 1384.5, 1306.6, 1175.4, 1117.7, 1034.4, 820.6. ^1^H-NMR (400 MHz, DMSO-*d*_6_) δ 9.16 (s, 1H), 8.89 (s, 1H), 8.16 (d, *J* = 5.6 Hz, 1H), 8.10 (d, *J* = 2.2 Hz, 1H), 7.63 (d, *J* = 2.2 Hz, 1H), 7.62 (s, 1H), 7.42 (d, *J* = 2.0 Hz, 1H), 7.40 (d, *J* = 2.2 Hz, 1H), 7.33 (d, *J* = 2.1 Hz, 1H), 7.32 (d, *J* = 2.0 Hz, 1H), 6.89 (d, *J* = 5.7 Hz, 1H), 4.23 (s, 2H), 4.09 (t, *J* = 6.2 Hz, 2H), 3.48 (t, *J* = 6.2 Hz, 2H), 3.25 (s, 3H), 2.13 (s, 3H), 2.00–1.94 (m, 2H). ^13^C-NMR (101 MHz, DMSO-*d*_6_) δ 163.08, 156.34, 152.76, 148.01, 139.76, 138.48, 132.45, 131.46, 128.91, 123.55, 122.80, 120.24, 119.57, 117.23, 106.48, 68.80, 65.44, 58.43, 31.14, 29.16, 10.89. ESI-MS (*m*/*z*): 540.2 ([M + H]^+^), 562.0 ([M + Na]^+^). HRMS (ESI) (*m*/*z*): Calcd. for C_25_H_25_ClF_3_N_3_O_3_S, 540.1330 ([M + H]^+^), found: 540.1320 ([M + H]^+^). Purity (HPLC): 97.33%.

*1-{4-{{[4-(3-Methoxypropoxy)-3-methylpyridin-2-yl]methyl}thio}phenyl}-3-[3-(trifluoromethyl)phenyl]urea* (**7e**)


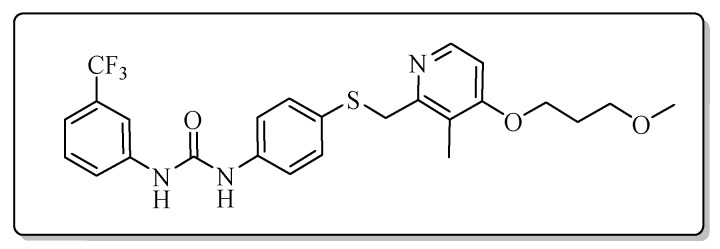


Compound **7e** was prepared according to the general procedure by using compound **4a** (0.32 g, 1 mmol) and 3-(trifluoromethyl)aniline (0.16 g, 1 mmol), obtained a white solid (0.27 g) in 53.1% yield. m.p. 136.1–137.9 °C. IR (KBr, cm^−1^): υ 3327.8, 2958.7, 2927.8, 2859.0, 2377.4, 2350.2, 2311.0, 1724.0, 1648.5, 1585.5, 1552.2, 1492.5, 1465.2, 1397.5, 1338.6, 1295.8, 1230.8, 1166.2, 1116.1, 1092.4, 1068.9, 1005.4, 890.0, 804.1, 732.9, 699.2, 602.3, 505.8. ^1^H-NMR (400 MHz, DMSO-*d*_6_) δ 9.05 (s, 1H), 8.84 (s, 1H), 8.17 (d, *J* = 5.6 Hz, 1H), 8.09 (s, 0H), 8.00 (d, *J* = 2.3 Hz, 1H), 7.57 (d, *J* = 8.4 Hz, 1H), 7.51 (t, *J* = 7.9 Hz, 1H), 7.42 (d, *J* = 2.0 Hz, 1H), 7.40 (s, 1H), 7.33 (d, *J* = 2.1 Hz, 1H), 7.32 (d, *J* = 2.1 Hz, 1H), 7.30 (s, 1H), 6.90 (d, *J* = 5.7 Hz, 1H), 4.23 (s, 2H), 4.09 (t, *J* = 6.2 Hz, 2H), 3.48 (t, *J* = 6.2 Hz, 2H), 3.25 (s, 3H), 2.13 (s, 3H), 2.01–1.94 (m, 3H). ^13^C-NMR (101 MHz, DMSO-*d*_6_) δ 163.11, 156.32, 152.85, 147.97, 140.98, 138.68, 131.55, 130.34, 129.10, 128.64, 122.30, 120.25, 119.40, 118.53, 106.46, 68.79, 65.44, 58.42, 31.13, 29.16, 10.88. ESI-MS (*m*/*z*): 506.3 ([M + H]^+^). HRMS (ESI) (*m*/*z*): Calcd. for C_25_H_26_F_3_N_3_O_3_S, 506.1720 ([M + H]^+^), found: 506.1728 ([M + H]^+^). Purity (HPLC): 97.09%.

*1-{4-{{[3-Methyl-4-(2,2,2-trifluoroethoxy)pyridin-2-yl]methyl}thio}phenyl}-3-phenylurea* (**7f**)


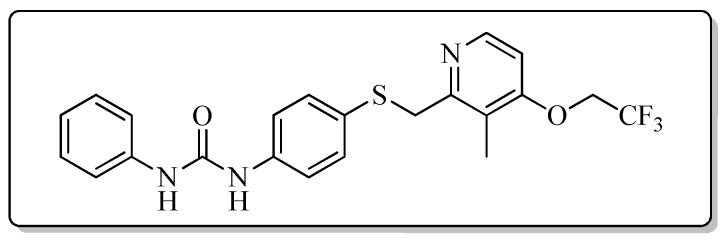


Compound **7f** was prepared according to the general procedure by using compound **4b** (0.33 g, 1 mmol) and aniline (0.10 g, 1 mmol), obtained a white solid (0.18 g) in 39.9% yield. m.p. 143.3–145.1 °C. IR (KBr, cm^−1^): υ 3424.1, 2923.9, 2852.6, 1687.8, 1639.9, 1600.0, 1548.5, 1495.9, 1441.2, 1399.4, 1384.7, 1307.8, 1284.4, 1266.4, 1232.1, 1176.6, 1112.2, 970.6, 915.9, 854.7, 836.1, 801.5, 783.1, 751.0, 696.2, 657.1, 618.8, 574.4. ^1^H-NMR (400 MHz, DMSO-*d*_6_) δ 8.73 (s, 1H), 8.67 (s, 1H), 8.24 (d, *J* = 5.7 Hz, 1H), 7.46 (s, 1H), 7.44 (s, 1H), 7.41 (s, 1H), 7.39 (s, 1H), 7.32 (s, 1H), 7.30 (s, 1H), 7.28 (s, 1H), 7.26 (s, 1H), 7.03 (d, *J* = 5.7 Hz, 1H), 6.97 (t, *J* = 7.3 Hz, 1H), 4.89 (q, *J* = 8.7 Hz, 2H), 4.25 (s, 2H), 2.16 (s, 3H). ^13^C-NMR (101 MHz, DMSO-*d*_6_) δ 161.62, 157.24, 152.87, 148.00, 140.05, 139.22, 131.90, 129.23, 127.85, 125.66, 122.90, 122.35, 120.44, 119.10, 118.71, 107.07, 64.92, 31.13, 10.74. ESI-MS (*m*/*z*): 448.4 ([M + H]^+^), 470.2 ([M + Na]^+^). HRMS (ESI) (*m*/*z*): Calcd. for C_22_H_20_F_3_N_3_O_2_S, 448.1301 ([M + H]^+^), found: 448.1295 ([M + H]^+^). Purity (HPLC): 97.04%.

*1-(4-Chlorophenyl)-3-{4-{{[3-methyl-4-(2,2,2-trifluoroethoxy)pyridin-2-yl]methyl}thio}phenyl}urea* (**7g**)


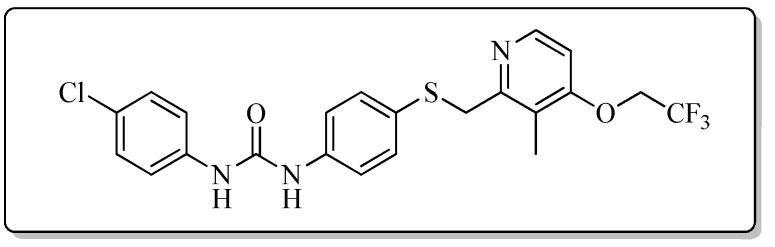


Compound **7g** was prepared according to the general procedure by using compound **4b** (0.33 g, 1 mmol) and 4-chloroaniline (0.13 g, 1 mmol), obtained a white solid (0.29 g) in 61.0% yield. m.p. 203.6–205.2 °C. IR (KBr, cm^−1^): υ 3424.1, 2984.9, 2923.1, 2851.9, 2350.0, 2311.0, 1611.4, 1548.6, 1491.9, 1440.3, 1399.6, 1384.9, 1370.1, 1311.1, 1268.3, 1172.6, 1111.7, 1051.4, 1004.4, 897.2, 798.3, 668.5, 615.4. ^1^H-NMR (400 MHz, DMSO-*d*_6_) δ 8.81 (s, 1H), 8.75 (s, 1H), 8.23 (d, *J* = 5.7 Hz, 1H), 7.48 (d, *J* = 2.1 Hz, 1H), 7.47 (d, *J* = 2.2 Hz, 1H), 7.40 (d, *J* = 2.0 Hz, 1H), 7.38 (s, 1H), 7.33 (d, *J* = 2.1 Hz, 1H), 7.32 (d, *J* = 2.2 Hz, 1H), 7.31 (s, 1H), 7.30 (d, *J* = 1.9 Hz, 1H), 7.03 (d, *J* = 5.7 Hz, 1H), 4.88 (q, *J* = 8.7 Hz, 2H), 4.25 (s, 2H), 2.16 (s, 3H). ^13^C-NMR (101 MHz, DMSO-*d*_6_) δ 161.62, 157.22, 152.77, 148.00, 139.08, 138.98, 131.80, 129.07, 128.11, 125.87, 122.90, 120.43, 120.23, 119.23, 107.08, 65.09, 31.14, 10.74. ESI-MS (*m*/*z*): 482.6 ([M + H]^+^), 504.3 ([M + Na]^+^). HRMS (ESI) (*m*/*z*): Calcd. for C_22_H_19_ClF_3_N_3_O_2_S, 482.0911 ([M + H]^+^), found: 482.0916 ([M + H]^+^). Purity (HPLC): 99.19%.

*1-(4-Methoxyphenyl)-3-{4-{{[3-methyl-4-(2,2,2-trifluoroethoxy)pyridin-2-yl]methyl}thio}phenyl}urea* (**7h**)


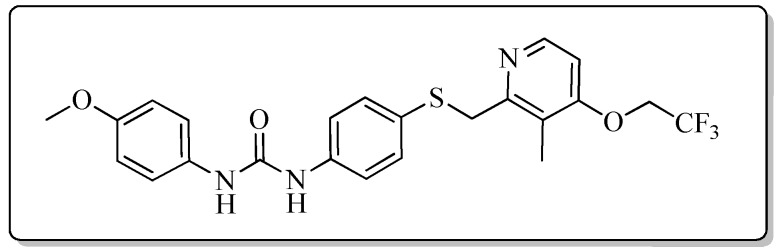


Compound **7h** was prepared according to the general procedure by using compound **4b** (0.33 g, 1 mmol) and 4-methoxyaniline (0.12 g, 1 mmol), obtained a white solid (0.22 g) in 45.9% yield. m.p. 171.3–172.1 °C. IR (KBr, cm^−1^): υ 3383.8, 2922.1, 2851.4, 2377.6, 2349.6, 1703.0, 1656.7, 1619.2, 1591.3, 1546.4, 1511.1, 1492.7, 1465.5, 1399.3, 1312.1, 1264.5, 1231.2, 1175.1, 1112.8, 1040.3, 1006.7, 970.2, 918.1, 897.2, 831.5, 799.7, 658.3. ^1^H-NMR (400 MHz, ) δ 8.64 (s, 1H), 8.47 (s, 1H), 8.16 (d, *J* = 5.7 Hz, 1H), 7.39 (d, *J* = 1.9 Hz, 1H), 7.37 (d, *J* = 2.2 Hz, 1H), 7.35 (d, *J* = 2.2 Hz, 1H), 7.33 (d, *J* = 2.3 Hz, 1H), 7.30 (d, *J* = 2.1 Hz, 1H), 7.29 (d, *J* = 2.0 Hz, 1H), 6.89 (d, *J* = 5.6 Hz, 1H), 6.87 (d, *J* = 2.2 Hz, 1H), 6.86 (d, *J* = 2.2 Hz, 1H), 4.91-4.85 (m, 2H), 4.21 (s, 2H), 3.71 (s, 3H), 2.12 (s, 3H). ^13^C-NMR (101 MHz, DMSO-*d*_6_) δ 161.84, 156.03, 154.81, 153.88, 153.39, 148.09, 133.72, 133.38, 125.65, 121.84, 120.37, 120.33, 115.40, 114.43, 108.04, 65.10, 55.63, 31.13, 10.44. ESI-MS (*m*/*z*): 478.3 ([M + H]^+^). HRMS (ESI) (*m*/*z*): Calcd. for C_23_H_22_F_3_N_3_O_3_S, 478.1407 ([M + H]^+^), found: 478.1408 ([M + H]^+^). Purity (HPLC): 99.66%.

*1-[4-Chloro-3-(trifluoromethyl)phenyl]-3-{4-{{[3-methyl-4-(2,2,2-trifluoroethoxy)pyridin-2-yl]methyl}thio}phenyl}urea* (**7i**)


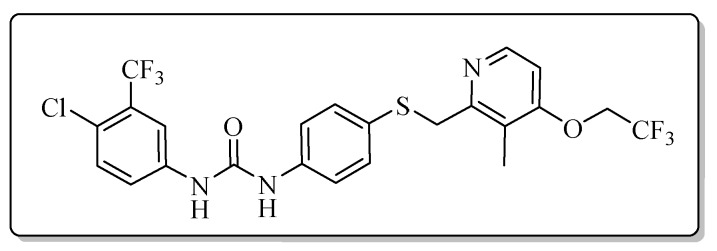


Compound **7i** was prepared according to the general procedure by using compound **4b** (0.33 g, 1 mmol) and 3-chloro-4-(trifluoromethyl)aniline (0.20 g, 1 mmol), obtained a white solid (0.27 g) in 49.5% yield. m.p. 142.0–143.0 °C. IR (KBr, cm^−1^): υ 3422.3, 2922.1, 2852.6, 1587.4, 1547.6, 1480.8, 1419.0, 1309.8, 1263.9, 1177.2, 1111.0, 1035.5, 974.7, 819.3, 618.3. ^1^H-NMR (400 MHz, DMSO-*d*_6_) δ 9.17 (s, 1H), 8.90 (s, 1H), 8.24 (d, *J* = 5.7 Hz, 1H), 8.10 (d, *J* = 2.2 Hz, 1H), 7.64 (dd, *J* = 8.9, 2.2 Hz, 1H), 7.61 (d, *J* = 8.7 Hz, 1H), 7.42 (d, *J* = 2.0 Hz, 1H), 7.40 (d, *J* = 2.2 Hz, 1H), 7.33 (d, *J* = 2.2 Hz, 1H), 7.32 (d, *J* = 2.1 Hz, 1H), 7.04 (d, *J* = 5.7 Hz, 1H), 4.89 (q, *J* = 8.7 Hz, 2H), 2.16 (s, 3H). ^13^C-NMR (101 MHz, DMSO-*d*_6_) δ 161.61, 157.18, 152.75, 148.00, 139.74, 138.60, 132.42, 131.64, 128.58, 127.33, 127.02, 125.65, 123.53, 122.80, 120.44, 119.55, 117.28, 117.22, 107.07, 64.79, 39.69, 10.72. ESI-MS (*m*/*z*): 550.1, 552.1, 553.1 ([M + H]^+^). HRMS (ESI) (*m*/*z*): Calcd. for C_23_H_18_ClF_6_N_3_O_2_S, 550.0785 ([M + H]^+^), found: 550.0769 ([M + H]^+^). Purity (HPLC): 99.97%.

*1-{4-{{[3-Methyl-4-(2,2,2-trifluoroethoxy)pyridin-2-yl]methyl}thio}phenyl}-3-[3-(trifluoromethyl)phenyl]urea* (**7j**)


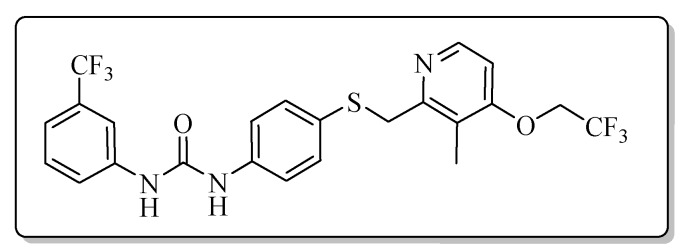


Compound **7j** was prepared according to the general procedure C by using compound **4b** (0.33 g, 1 mmol) and 3-(trifluoromethyl)aniline (0.16 g, 1 mmol), obtained a white solid (0.34 g) in 66.1% yield. m.p. 174.7–176.4 °C. IR (KBr, cm^−1^): υ 3421.4, 2985.6, 2924.1, 2852.9, 2349.2, 2311.0, 1614.9, 1491.8, 1445.1, 1399.1, 1339.8, 1313.1, 1288.0, 1264.6, 1231.2, 1173.2, 1114.6, 1071.2, 1006.3, 976.0, 832.4, 798.2, 700.6, 616.4. ^1^H-NMR (400 MHz, DMSO-*d*_6_) δ 9.07 (s, 1H), 8.87 (s, 1H), 8.24 (d, *J* = 5.7 Hz, 1H), 8.01 (d, *J* = 2.0 Hz, 1H), 7.60–7.54 (m, 1H), 7.51 (t, *J* = 7.9 Hz, 1H), 7.43 (d, *J* = 2.0 Hz, 1H), 7.41 (d, *J* = 2.1 Hz, 1H), 7.33 (d, *J* = 2.1 Hz, 1H), 7.33–7.31 (m, 1H), 7.30 (d, *J* = 1.7 Hz, 1H), 7.04 (d, *J* = 5.7 Hz, 1H), 4.89 (q, *J* = 8.7 Hz, 2H), 4.26 (s, 2H), 2.16 (s, 3H). ^13^C-NMR (101 MHz, DMSO-*d*_6_) δ 161.66, 157.16, 152.86, 147.96, 140.97, 138.80, 131.76, 130.35, 130.15, 129.84, 128.31, 122.30, 120.47, 119.40, 118.54, 114.59, 107.09, 65.09, 49.05, 31.13, 10.73. ESI-MS (*m*/*z*): 516.2 ([M + H]^+^). HRMS (ESI) (*m*/*z*): Calcd. for C_23_H_19_F_6_N_3_O_2_S, 516.1175 ([M + H]^+^), found: 516.1174 ([M + H]^+^). Purity (HPLC): 97.29%.

*1-{4-{[(4-Methoxy-3,5-dimethylpyridin-2-yl)methyl]thio}phenyl}-3-phenylurea* (**7k**)


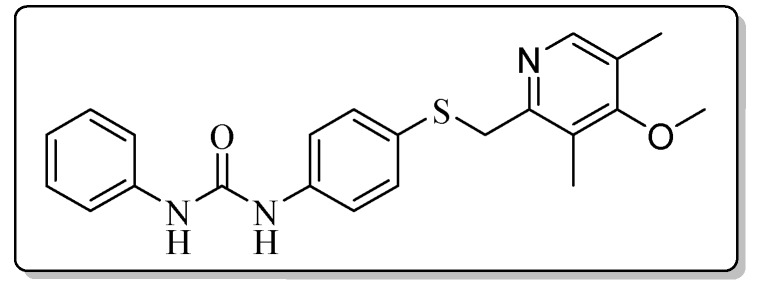


Compound **7k** was prepared according to the general procedure by using compound **4c** (0.27 g, 1 mmol) and aniline (0.10 g, 1 mmol), obtained a white solid (0.23 g) in 57.3% yield. m.p. 188.1–188.9 °C. IR (KBr, cm^−1^): υ 3422.4, 2923.8, 2852.4, 2351.0, 2321.9, 1644.0, 1597.4, 1553.7, 1494.2, 1441.5, 1398.0, 1312.9, 1270.7, 1237.5, 1173.3, 1127.7, 1073.5, 1002.5, 798.1, 755.7, 738.0, 694.0, 616.4. ^1^H-NMR (400 MHz, DMSO-*d*_6_) δ 8.72 (s, 1H), 8.67 (s, 1H), 8.12 (s, 1H), 7.45 (d, *J* = 1.3 Hz, 1H), 7.44-7.42 (m, 1H), 7.40 (d, *J* = 2.1 Hz, 1H), 7.39 (d, *J* = 2.1 Hz, 1H), 7.31 (d, *J* = 2.2 Hz, 1H), 7.29 (d, *J* = 2.1 Hz, 1H), 7.28 (s, 1H), 7.26 (d, *J* = 1.6 Hz, 1H), 6.97 (t, *J* = 7.4 Hz, 1H), 4.21 (s, 2H), 3.70 (s, 3H), 2.18 (s, 6H). ^13^C-NMR (101 MHz, DMSO-*d*_6_) δ 163.86, 155.80, 152.86, 148.97, 140.05, 139.16, 131.86, 129.24, 127.98, 125.19, 125.03, 122.35, 119.11, 118.70, 60.17, 31.15, 13.38, 11.34. ESI-MS (*m*/*z*): 394.6 ([M + H]^+^), 416.3 ([M + Na]^+^). HRMS (ESI) (*m*/*z*): Calcd. for C_22_H_23_N_3_O_2_S, 394.1584 ([M + H]^+^), found: 394.1586 ([M + H]^+^). Purity (HPLC): 99.89%.

*1-(4-Chlorophenyl)-3-{4-{[(4-methoxy-3,5-dimethylpyridin-2-yl)methyl]thio}phenyl}urea* (**7l**)


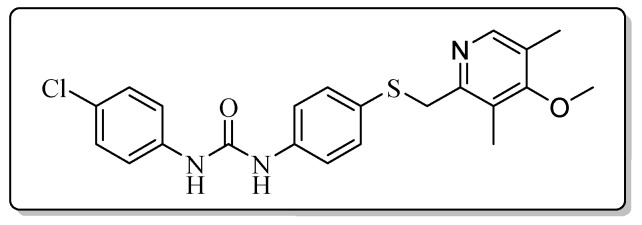


Compound **7l** was prepared according to the general procedure by using compound **4c** (0.27 g, 1 mmol) and 4-chloroaniline (0.13 g, 1 mmol), obtained a white solid (0.28 g) in 66.2% yield. m.p. 206.8–208.2 °C. IR (KBr, cm^−1^): υ 3422.5, 2923.0, 2852.0, 2377.1, 2349.6, 2310.8, 1630.4, 1547.7, 1491.7, 1439.7, 1399.2, 1385.1, 1309.9, 1270.9, 1235.5, 1173.0, 1124.1, 1051.3, 1004.6, 832.1, 798.2, 702.1, 668.3, 617.0. ^1^H-NMR (400 MHz, DMSO-*d*_6_) δ 8.81 (s, 1H), 8.75 (s, 1H), 8.11 (s, 1H), 7.48 (d, *J* = 2.1 Hz, 1H), 7.46 (d, *J* = 2.2 Hz, 1H), 7.40 (d, *J* = 2.0 Hz, 1H), 7.38 (d, *J* = 2.2 Hz, 1H), 7.33 (d, *J* = 2.1 Hz, 1H), 7.32 (s, 1H), 7.31 (s, 1H), 7.30 (d, *J* = 2.1 Hz, 1H), 4.21 (s, 2H), 3.70 (s, 3H), 2.18 (s, 3H), 2.18 (s, 3H). ^13^C-NMR (101 MHz, DMSO-*d*_6_) δ 163.86, 155.79, 152.77, 148.97, 139.08, 138.94, 131.76, 129.07, 127.98, 128.25, 125.87, 125.19, 125.03, 120.23, 119.25, 60.17, 31.14, 13.37, 11.33. ESI-MS (*m*/*z*): 428.7 ([M + H]^+^). HRMS (ESI) (*m*/*z*): Calcd. for C_22_H_22_ClN_3_O_2_S, 428.1194 ([M + H]^+^), found: 428.1199 ([M + H]^+^). Purity (HPLC): 99.53%.

*1-{4-{[(4-Methoxy-3,5-dimethylpyridin-2-yl)methyl)]thio}phenyl}-3-(4-methoxyphenyl)urea* (**7m**)


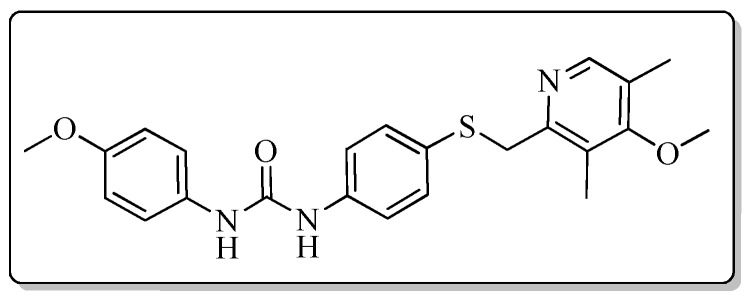


Compound **7m** was prepared according to the general procedure by using compound **4c** (0.27 g, 1 mmol) and 4-methoxyaniline (0.12 g, 1 mmol), obtained a white solid (0.21 g) in 49.2% yield. m.p. 171.4–172.6 °C. IR (KBr, cm^−1^): υ 3422.5, 2921.1, 2850.5, 1642.5, 1593.2, 1547.5, 1493.7, 1468.2, 1439.0, 1397.7, 1311.5, 1292.4, 1270.1, 1240.4, 1173.4, 1073.2, 1053.2, 1031.3, 1003.9, 828.2, 797.9, 616.3. ^1^H-NMR (400 MHz, DMSO-*d*_6_) δ 8.68 (s, 1H), 8.50 (s, 1H), 8.15 (s, 1H), 7.39 (d, *J* = 1.9 Hz, 1H), 7.37 (d, *J* = 2.1 Hz, 1H), 7.35 (d, *J* = 2.0 Hz, 1H), 7.33 (d, *J* = 2.2 Hz, 1H), 7.29 (d, *J* = 2.1 Hz, 1H), 7.27 (d, *J* = 1.9 Hz, 1H), 6.87 (d, *J* = 2.3 Hz, 1H), 6.86 (d, *J* = 2.2 Hz, 1H), 4.21 (s, 2H), 3.73 (s, 3H), 3.71 (s, 3H), 2.20 (s, 3H), 2.17 (s, 3H). ^13^C-NMR (101 MHz, DMSO-*d*_6_) δ 164.47, 155.40, 154.98, 153.08, 148.27, 139.64, 133.09, 132.28, 127.19, 125.70, 125.46, 125.41, 120.49, 118.95, 118.40, 114.46, 60.30, 55.65, 31.14, 13.45, 11.35. ESI-MS (*m*/*z*): 424.3 ([M + H]^+^). HRMS (ESI) (*m*/*z*): Calcd. for C_23_H_25_N_3_O_3_S, 424.1689 ([M + H]^+^), found: 424.1698 ([M + H]^+^). Purity (HPLC): 96.88%.

*1-[4-Chloro-3-(trifluoromethyl)phenyl]-3-{4-{[(4-methoxy-3,5-dimethylpyridin-2-yl)methyl]thio}phenyl}urea* (**7n**)


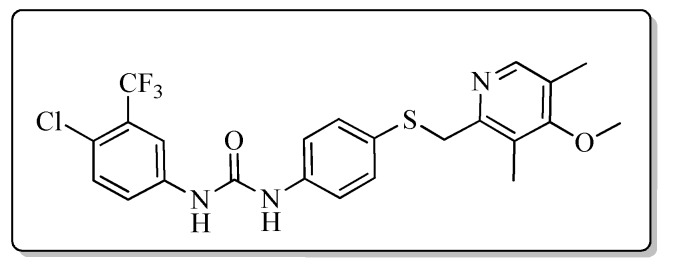


Compound **7n** was prepared according to the general procedure by using compound **4c** (0.27 g, 1 mmol) and 3-chloro-4-(trifluoromethyl)aniline (0.20 g, 1 mmol), obtained a white solid (0.35 g) in 70.1% yield. m.p. 152.3–153.1 °C. IR (KBr, cm^−1^): υ 3422.2, 2921.6, 2852.2, 1719.2, 1593.8, 1546.4, 1480.4, 1419.8, 1384.6, 1311.1, 1265.2, 1229.0, 1174.6, 1130.8, 1073.5, 1031.7, 823.3, 619.9. ^1^H-NMR (400 MHz, DMSO-*d*_6_) δ 9.18 (s, 1H), 8.91 (s, 1H), 8.12 (s, 1H), 8.10 (d, *J* = 2.0 Hz, 1H), 7.64 (d, *J* = 8.9 Hz, 1H), 7.62 (s, 1H), 7.42 (d, *J* = 2.1 Hz, 1H), 7.40 (d, *J* = 2.1 Hz, 1H), 7.33 (s, 1H), 7.31 (d, *J* = 1.9 Hz, 1H), 4.22 (s, 2H), 3.70 (s, 3H), 2.19 (s, 3H), 2.18 (s, 3H). ^13^C-NMR (101 MHz, DMSO-*d*_6_) δ 164.07, 154.69, 154.49, 153.03, 149.00, 140.01, 132.84, 132.38, 126.45, 126.36, 124.20, 123.34, 122.49, 121.03, 115.40, 60.18, 31.13, 13.43, 11.00. ESI-MS (*m*/*z*): 496.1; 497.1; 498.1; 499.1 ([M + H]^+^). HRMS (ESI) (*m*/*z*): Calcd. for C_23_H_21_ClF_3_N_3_O_2_S, 496.1068 ([M + H]^+^), found: 496.1066 ([M + H]^+^). Purity (HPLC): 98.56%.

*1-{4-{[(4-Methoxy-3,5-dimethylpyridin-2-yl)methyl]thio}phenyl}-3-[3-(trifluoromethyl)pHenyl]urea* (**7o**)


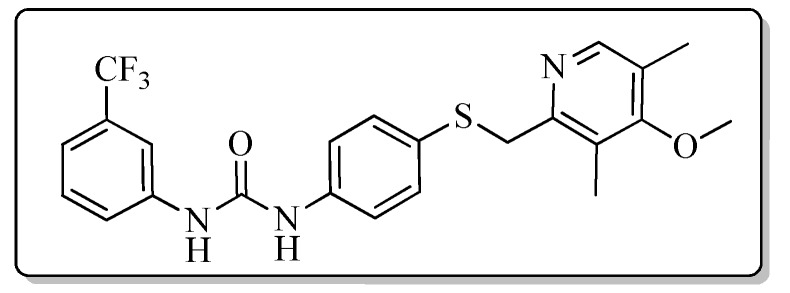


Compound **7o** was prepared according to the general procedure by using compound **4c** (0.27 g, 1 mmol) and 3-(trifluoromethyl)aniline (0.16 g, 1 mmol), obtained a white solid (0.30 g) in 64.7% yield. m.p. 156.9–158.1 °C. IR (KBr, cm^−1^): υ 3420.6, 2984.8, 2922.8, 2851.8, 2350.3, 2321.1, 1609.8, 1491.8, 1443.2, 1398.4, 1369.9, 1338.2, 1311.7, 1271.2, 1229.2, 1172.1, 1124.2, 1072.1, 1002.9, 797.9, 698.2, 616.0. ^1^H-NMR (400 MHz, DMSO-*d*_6_) δ 9.04 (s, 1H), 8.84 (s, 1H), 8.12 (s, 1H), 8.00 (d, *J* = 2.0 Hz, 1H), 7.57 (d, *J* = 8.8 Hz, 1H), 7.51 (t, *J* = 7.8 Hz, 1H), 7.42 (d, *J* = 1.9 Hz, 1H), 7.40 (d, *J* = 2.2 Hz, 1H), 7.33 (s, 1H), 7.32 (s, 1H), 7.30 (d, *J* = 2.6 Hz, 1H), 4.22 (s, 2H), 3.70 (s, 3H), 2.19 (s, 3H), 2.18 (s, 3H). ^13^C-NMR (101 MHz, DMSO-*d*_6_) δ 163.88, 155.76, 152.85, 148.95, 140.97, 138.73, 131.69, 130.35, 128.51, 125.21, 125.05, 123.31, 122.32, 119.43, 118.58, 114.64, 60.16, 31.13, 13.36, 11.33. ESI-MS (*m*/*z*): 462.3 ([M + H]^+^). HRMS (ESI) (*m*/*z*): Calcd. for C_23_H_22_F_3_N_3_O_2_S, 462.1458 ([M + H]^+^), found: 462.1469 ([M + H]^+^). Purity (HPLC): 99.79%.

*1-{4-{[(3,4-Dimethoxypyridin-2-yl)methyl]thio}phenyl}-3-phenylurea* (**7p**)


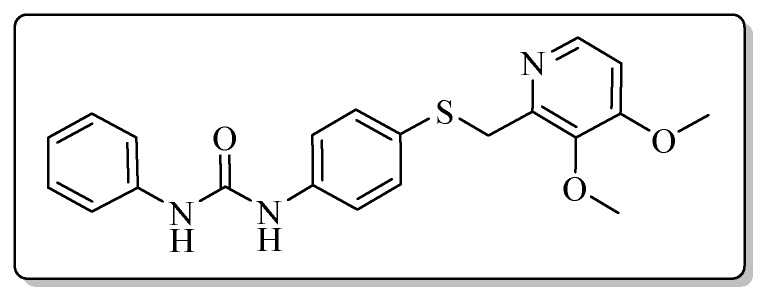


Compound **7p** was prepared according to the general procedure by using compound **4d** (0.28 g, 1 mmol) and aniline (0.10 g, 1 mmol), obtained a white solid (0.18 g) in 45.8% yield. m.p. 127.7–128.5 °C. IR (KBr, cm^−1^): υ 3287.2, 2937.3, 1654.0, 1593.7, 1548.0, 1487.0, 1442.7, 1421.2, 1379.4, 1297.9, 1270.4, 1231.6, 1175.0, 1071.7, 997.3, 932.8, 829.0, 782.9, 742.9, 692.4, 618.5, 516.5. ^1^H-NMR (400 MHz, DMSO-*d*_6_) δ 8.72 (s, 1H), 8.67 (s, 1H), 8.12 (d, *J* = 5.5 Hz, 1H), 7.49–7.45 (m, 1H), 7.44 (s, 1H), 7.41 (d, *J* = 2.0 Hz, 1H), 7.40 (d, *J* = 2.2 Hz, 1H), 7.33 (d, *J* = 2.0 Hz, 1H), 7.31 (d, *J* = 1.9 Hz, 1H), 7.29 (d, *J* = 7.7 Hz, 1H), 7.28–7.25 (m, 1H), 7.03 (d, *J* = 5.5 Hz, 1H), 6.97 (t, *J* = 7.3 Hz, 1H), 4.17 (s, 2H), 3.87 (s, 3H), 3.74 (s, 3H). ^13^C-NMR (101 MHz, DMSO-*d*_6_) δ 158.52, 152.88, 151.53, 145.72, 143.40, 138.98, 131.39, 129.24, 128.54, 122.33, 119.17, 118.69, 108.29, 60.99, 56.34, 36.37, 31.14. ESI-MS (*m*/*z*): 396.3 ([M + H]^+^), 418.2 ([M + Na]^+^). HRMS (ESI) (*m*/*z*): Calcd. for C_21_H_21_N_3_O_3_S, 396.1376 ([M + H]^+^), found: 396.1380 ([M + H]^+^). Purity (HPLC): 99.90%.

*1-(4-Chlorophenyl)-3-{4-{[(3,4-dimethoxypyridin-2-yl)methyl]thio}phenyl}urea* (**7q**)


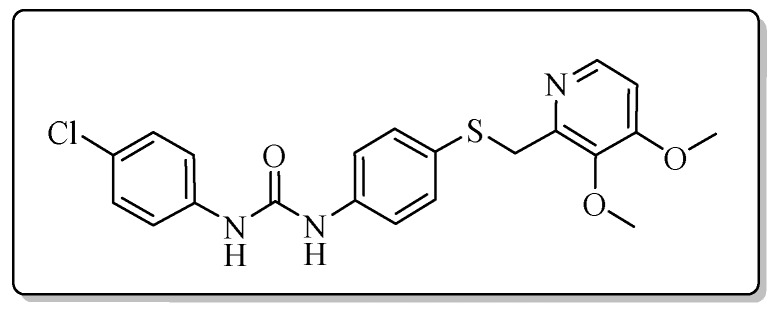


Compound **7q** was prepared according to the general procedure by using compound **4d** (0.28 g, 1 mmol) and 4-chloroaniline (0.13 g, 1 mmol), obtained a white solid (0.21 g) in 49.1% yield. m.p. 141.7–142.9 °C. IR (KBr, cm^−1^): υ 3345.3, 3096.8, 2924.2, 2852.2, 1711.6, 1631.2, 1590.8, 1535.1, 1490.0, 1449.2, 1427.9, 1399.4, 1300.6, 1284.5, 1237.1, 1195.2, 1174.0, 1087.1, 1067.2, 996.9, 828.3, 703.0, 509.1. ^1^H-NMR (400 MHz, DMSO-*d*_6_) δ 8.83 (s, 1H), 8.76 (s, 1H), 8.11 (d, *J* = 5.5 Hz, 1H), 7.49 (s, 1H), 7.47 (s, 1H), 7.41 (s, 1H), 7.39 (s, 1H), 7.34-7.31 (m, 2H), 7.03 (d, *J* = 5.5 Hz, 1H), 4.17 (s, 2H), 3.87 (s, 3H), 3.74 (s, 3H). ^13^C-NMR (101 MHz, DMSO-*d*_6_) δ 158.52, 152.78, 151.50, 145.71, 143.40, 139.10, 138.75, 131.29, 129.07, 128.79, 125.85, 120.21, 119.30, 108.30, 60.99, 56.34, 36.29, 31.14. ESI-MS (*m*/*z*): 430.6 ([M + H]^+^), 452.1 ([M + Na]^+^). HRMS (ESI) (*m*/*z*): Calcd. for C_21_H_20_ClN_3_O_3_S, 430.0987 ([M + H]^+^), found: 430.0993 ([M + H]^+^). Purity (HPLC): 99.33%.

*1-{4-{[(3,4-Dimethoxypyridin-2-yl)methyl]thio}phenyl}-3-(4-methoxyphenyl)urea* (**7r**)


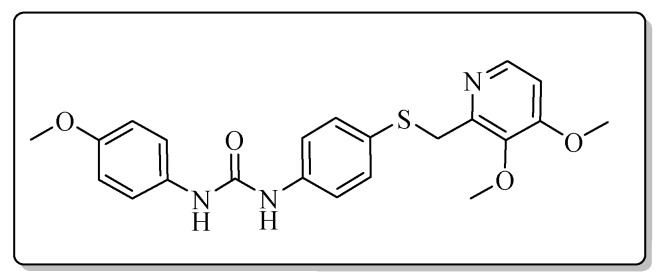


Compound **7r** was prepared according to the general procedure by using compound **4d** (0.28 g, 1 mmol) and 4-methoxyaniline (0.12 g, 1 mmol), obtained a white solid (0.20 g) in 47.9% yield. m.p. 179.0–180.6 °C. IR (KBr, cm^−1^): υ 3428.5, 2985.2, 2923.2, 2851.3, 1630.7, 1587.2, 1557.0, 1510.3, 1490.9, 1442.6, 1398.6, 1299.7, 1270.7, 1232.4, 1173.6, 1072.2, 1033.0, 1000.9, 934.0, 829.1, 799.5, 617.4, 549.0, 523.3. ^1^H-NMR (400 MHz, DMSO-*d*_6_) δ 8.62 (s, 1H), 8.46 (s, 1H), 8.11 (d, *J* = 5.5 Hz, 1H), 7.39 (d, *J* = 2.0 Hz, 1H), 7.38 (d, *J* = 2.2 Hz, 1H), 7.35 (d, *J* = 2.2 Hz, 1H), 7.33 (d, *J* = 2.2 Hz, 1H), 7.31 (d, *J* = 2.2 Hz, 1H), 7.30 (d, *J* = 2.0 Hz, 1H), 7.03 (d, *J* = 5.5 Hz, 1H), 6.87 (d, *J* = 2.2 Hz, 1H), 6.85 (d, *J* = 2.2 Hz, 1H), 4.15 (s, 2H), 3.87 (s, 3H), 3.74 (s, 3H), 3.71 (s, 3H). ^13^C-NMR (101 MHz, DMSO-*d*_6_) δ 158.51, 154.98, 153.07, 151.55, 145.73, 143.40, 139.22, 133.08, 131.45, 128.24, 120.54, 119.06, 114.46, 108.30, 60.99, 56.35, 55.65, 36.43, 31.15. ESI-MS (*m*/*z*): 426.3 ([M + H]^+^). HRMS (ESI) (*m*/*z*): Calcd. for C_22_H_23_N_3_O_4_S, 426.1482 ([M + H]^+^), found: 426.1489 ([M + H]^+^). Purity (HPLC): 98.84%.

*1-[4-Chloro-3-(trifluoromethyl)phenyl]-3-{4-{[(3,4-dimethoxypyridin-2-yl)methyl]thio}pHenyl}urea* (**7s**)


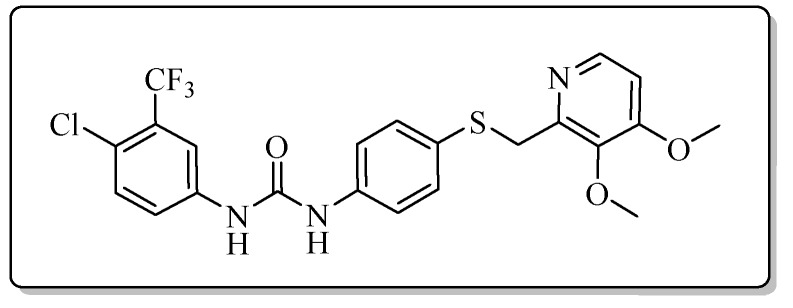


Compound **7s** was prepared according to the general procedure by using compound **4d** (0.28 g, 1 mmol) and 3-chloro-4-(trifluoromethyl)aniline (0.20 g, 1 mmol), obtained a white solid (0.32 g) in 64.5% yield. m.p. 188.1–189.2 °C. IR (KBr, cm^−1^): υ 3425.5, 2921.9, 2852.4, 1589.9, 1545.2, 1485.2, 1419.2, 1384.4, 1306.1, 1229.2, 1175.6, 1132.0, 1068.8, 1033.0, 824.9. ^1^H-NMR (400 MHz, DMSO-*d*_6_) δ 9.15 (s, 1H), 8.88 (s, 1H), 8.11 (d, *J* = 5.5 Hz, 1H), 8.10 (d, *J* = 2.2 Hz, 1H), 7.64 (d, *J* = 8.8 Hz, 1H), 7.62–7.58 (m, 1H), 7.42 (d, *J* = 2.0 Hz, 1H), 7.41 (d, *J* = 2.2 Hz, 1H), 7.34 (d, *J* = 2.2 Hz, 1H), 7.33 (d, *J* = 2.0 Hz, 1H), 7.03 (d, *J* = 5.5 Hz, 1H), 4.17 (s, 2H), 3.88 (s, 3H), 3.74 (s, 3H). ^13^C-NMR (101 MHz, DMSO-*d*_6_) δ 158.52, 152.76, 151.47, 145.73, 143.40, 139.76, 138.36, 132.44, 131.12, 129.28, 123.53, 122.78, 119.62, 117.24, 108.32, 61.00, 56.35, 36.17, 31.14. ESI-MS (*m*/*z*): 498.2 ([M + H]^+^). HRMS (ESI) (*m*/*z*): Calcd. for C_22_H_19_ClF_3_N_3_O_3_S, 498.0861 ([M + H]^+^), found: 498.0844 ([M + H]^+^). Purity (HPLC): 98.10%.

*1-{4-{[(3,4-Dimethoxypyridin-2-yl)methyl]thio}phenyl}-3-[3-(trifluoromethyl)phenyl]urea* (**7t**)


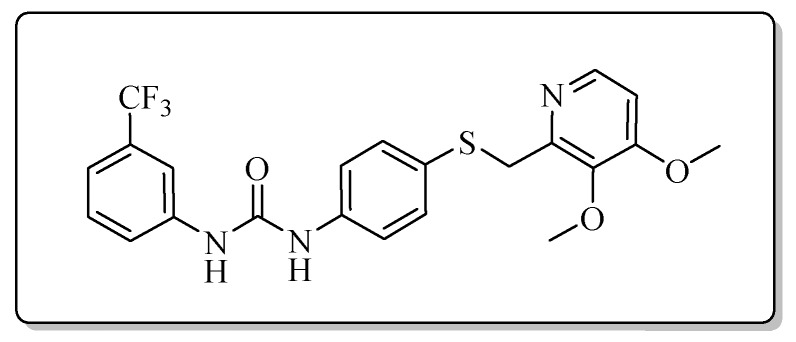


Compound **7t** was prepared according to the general procedure by using compound **4d** (0.28 g, 1 mmol) and 3-(trifluoromethyl)aniline (0.16 g, 1 mmol), obtained a white solid (0.21 g) in 44.3% yield. m.p. 198.4–199.8 °C. IR (KBr, cm^−1^): υ 3422.2, 2985.4, 2377.5, 2349.8, 2320.7, 2024.8, 1712.9, 1594.1, 1564.4, 1537.2, 1491.3, 1445.8, 1399.4, 1370.2, 1316.3, 1273.6, 1230.1, 1173.8, 1124.9, 1068.3, 1002.1, 932.4, 892.8, 828.2, 798.3, 743.3, 697.8, 615.6. ^1^H-NMR (400 MHz, DMSO-*d*_6_) δ 9.04 (s, 1H), 8.83 (s, 1H), 8.11 (d, *J* = 5.5 Hz, 1H), 8.01 (d, *J* = 2.0 Hz, 1H), 7.60–7.54 (m, 1H), 7.51 (t, *J* = 7.9 Hz, 1H), 7.43 (d, *J* = 2.0 Hz, 1H), 7.41 (d, *J* = 2.2 Hz, 1H), 7.34 (d, *J* = 2.2 Hz, 1H), 7.32 (d, *J* = 2.5 Hz, 1H), 7.30 (s, 1H), 7.03 (d, *J* = 5.6 Hz, 1H), 4.17 (s, 2H), 3.88 (s, 3H), 3.75 (s, 3H). ^13^C-NMR (101 MHz, DMSO-*d*_6_) δ 158.52, 152.87, 151.50, 145.72, 143.41, 140.98, 138.55, 131.22, 130.34, 129.08, 122.31, 119.49, 118.56, 114.65, 108.31, 60.99, 56.34, 36.24, 31.12. ESI-MS (*m*/*z*): 464.2 ([M + H]^+^). Purity (HPLC): 98.85%.

### 3.2. Biological Evaluation

#### 3.2.1. Antiproliferative Activity Assays

The antiproliferative activities of target compounds were determined using a standard MTT assay [27,28,29,30]. Exponentially growing cells A549 (3 × 10^3^ cells/well), HCT-116 (1 × 10^4^ cells/well) and PC-3 (8 × 10^3^ cells/well) were seeded into 96-well plates and incubated for 24 h to allow the cells to attach. After 24 h of incubation, the culture medium was removed and fresh medium containing various concentrations of the candidate compounds was added to each well. The cells were then incubated for 72 h, thereafter MTT assays were performed and cell viability was assessed at 570 nm by a microplate reader (ThermoFisher Scientific (Shanghai) Instrument Co., Ltd., Shanghai, China).

#### 3.2.2. Cell Apoptosis Assay

A549 cells were seeded into a 6-well plate (2 × 10^5^/well) and incubated for 24 h. Then the cells were treated with different concentrations of the tested compound **7i** for 24 h. Thereafter, the cells were collected and the Annexin-V-FITC/PI apoptosis kit (Biovision, Milpitas, CA, USA) was used according to the manufacturer’s protocol. The cells were analyzed by Accuri C6 flow cytometric (Becton Dickinson, Franklin Lakes, NJ, USA) [31].

#### 3.2.3. Cell Cycle Analysis

For flow cytometric analysis of DNA content, 5 × 10^5^ A549 cells in exponential growth were treated with different concentrations of the compound **7i** for 24 or 48 h. After an incubation period, the cells were collected, centrifuged and fixed with ice cold ethanol (70%). The cells were then treated with buffer containing RNAse A and 0.1% Triton X-100 and then stained with the propidium iodide (PI). The samples were analyzed on Accuri C6 flow cytometer (Becton Dickinson). [32].

## 4. Conclusions

In summary, a new series of 1-aryl-3-{4-[(pyridin-2-ylmethyl)thio]phenyl}urea derivatives were designed and synthesized based on molecular hybridization strategy. Majority of target compounds showed moderate to good growth inhibition against the tested cancer cells. Particularly, compound **7i** exhibited more potent antiproliferative activity than well-known anticancer drug sorafenib against all three cancer cell lines (A549, HCT-116 and PC-3). The preliminary mechanism investigation showed that compound **7i** could induce A549 cells to apoptosis, and halted cell cycle progression at the G1 phase. The SARs illustrated that these target compounds in this work might serve as bioactive fragments, and compound **7i** could be used as a lead compound for the development of potent cancer chemotherapeutic agents in the drug discovery process.

## Figures and Tables

**Table 1:**
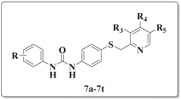
The chemical structures and inhibitory activities of the target compounds.

Compound	R	R_3_	R_4_	R_5_	IC_50_ (μM) ^a^		
					A549	HCT-116	PC-3
**7a**	H	-CH_3_	-O(CH_2_)_3_OCH_3_	H	12.31 ± 1.90	29.26 ± 4.53	27.22 ± 3.36
**7b**	4-chloro	-CH_3_	-O(CH_2_)_3_OCH_3_	H	3.03 ± 2.79	4.80 ± 1.57	6.00 ± 0.22
**7c**	4-methoxy	-CH_3_	-O(CH_2_)_3_OCH_3_	H	6.35 ± 0.51	5.29 ± 0.22	6.95 ± 1.24
**7d**	4-chloro-3-trifluoromethyl	-CH_3_	-O(CH_2_)_3_OCH_3_	H	2.02 ± 2.20	1.94 ± 3.45	3.97 ± 1.02
**7e**	3-trifluoromethyl	-CH_3_	-O(CH_2_)_3_OCH_3_	H	2.90 ± 0.36	3.78 ± 1.41	11.48 ± 0.98
**7f**	H	-CH_3_	-OCH_2_CF_3_	H	14.29 ± 1.77	19.31 ± 3.64	20.40 ± 2.89
**7g**	4-chloro	-CH_3_	-OCH_2_CF_3_	H	2.23 ± 1.27	3.04 ± 0.37	6.78 ± 0.23
**7h**	4-methoxy	-CH_3_	-OCH_2_CF_3_	H	4.71 ± 0.11	12.09 ± 1.46	15.65 ± 0.69
**7i**	4-chloro-3-trifluoromethyl	-CH_3_	-OCH_2_CF_3_	H	1.53 ± 0.46	1.11 ± 0.34	1.98 ± 1.27
**7j**	3-trifluoromethyl	-CH_3_	-OCH_2_CF_3_	H	4.22 ± 0.99	10.69 ± 0.87	14.33 ± 3.24
**7k**	H	-CH_3_	-OCH_3_	-CH_3_	13.37 ± 0.81	>100	>100
**7l**	4-chloro	-CH_3_	-OCH_3_	-CH_3_	2.23 ± 1.38	3.20 ± 2.75	5.97 ± 0.55
**7m**	4-methoxy	-CH_3_	-OCH_3_	-CH_3_	5.27 ± 1.01	3.49 ± 0.78	6.95 ± 0.35
**7n**	4-chloro-3-trifluoromethyl	-CH_3_	-OCH_3_	-CH_3_	2.63 ± 1.25	2.51 ± 0.15	6.32 ± 1.68
**7o**	3-trifluoromethyl	-CH_3_	-OCH_3_	-CH_3_	4.88 ± 1.02	3.50 ± 0.13	7.18 ± 1.58
**7p**	H	-OCH_3_	-OCH_3_	H	26.98 ± 4.12	45.93 ± 6.65	30.95 ± 5.41
**7q**	4-chloro	-OCH_3_	-OCH_3_	H	14.52 ± 2.67	5.15 ± 1.01	18.57 ± 2.34
**7r**	4-methoxy	-OCH_3_	-OCH_3_	H	16.47 ± 4.51	33.10 ± 2.90	22.51 ± 3.07
**7s**	4-chloro-3-trifluoromethyl	-OCH_3_	-OCH_3_	H	6.23 ± 0.60	5.61 ± 0.66	10.05 ± 2.23
**7t**	3-trifluoromethyl	-OCH_3_	-OCH_3_	H	14.13 ± 1.82	17.37 ± 1.69	19.77 ± 0.74
sorafenib					2.12 ± 0.18	2.25 ± 0.71	3.60 ± 1.08

^a^ Inhibitory activity was assayed by exposure for 72 h to substance and expressed as the concentration required to inhibit tumor cell proliferation by 50% (IC_50_). Data are presented as the means ± SEMs of three independent experiments.

## Supplementary Materials

The following are available online. ^1^H-NMR, ^13^C-NMR, ESI-MS and HRMS of the target compounds, respectively.
