# Supplementary material for: Design, Synthesis and Biological Evaluation of a New Series of 1-Aryl-3-{4-[(pyridin-2-ylmethyl)thio]phenyl}urea Derivatives as Antiproliferative Agents
Source: Molecules. 2019 Jun 4;24(11):2108. doi: 10.3390/molecules24112108 (PMC6600452; doi:10.3390/molecules24112108)
Supplement: Supplementary file 1 [file molecules-24-02108-s001.pdf]

molecules

Supplementary Materials

Design, synthesis and biological evaluation of a new series of 1-aryl-3-{4-[(pyridin-2-ylmethyl)thio]phenyl}urea derivatives as antiproliferative agents

Chuanming Zhang [1], Xiaoyu Tan [1], Jian Feng [1], Ning Ding [1], Yongpeng Li [1], Zhe Jin [1], Qingguo Meng [2], Xiaoping Liu [1,*] and Chun Hu [1,*]

[1] Key Laboratory of Structure-Based Drug Design & Discovery, Ministry of Education, Shenyang Pharmaceutical University, Shenyang 110016, China; chuanming_zhang@yeah.net (C.Z.); tanxiaoyuaikaoyan@163.com (X.T.); mr.fengjian@foxmail.com (J.F.); dingning2216@163.com (N.D.); yongpengli1993@163.com (Y.L.); jinzheln@163.com (Z.J.);

[2] Department of Pharmacy, Yantai University, Yantai 264005, China; qinggmeng@163.com.

* Correspondence: lxp19730107@163.com (X.L.); chunhu@syphu.edu.cn (C.H.); Tel.: +86-24-43520246 (X.L. & C.H.)

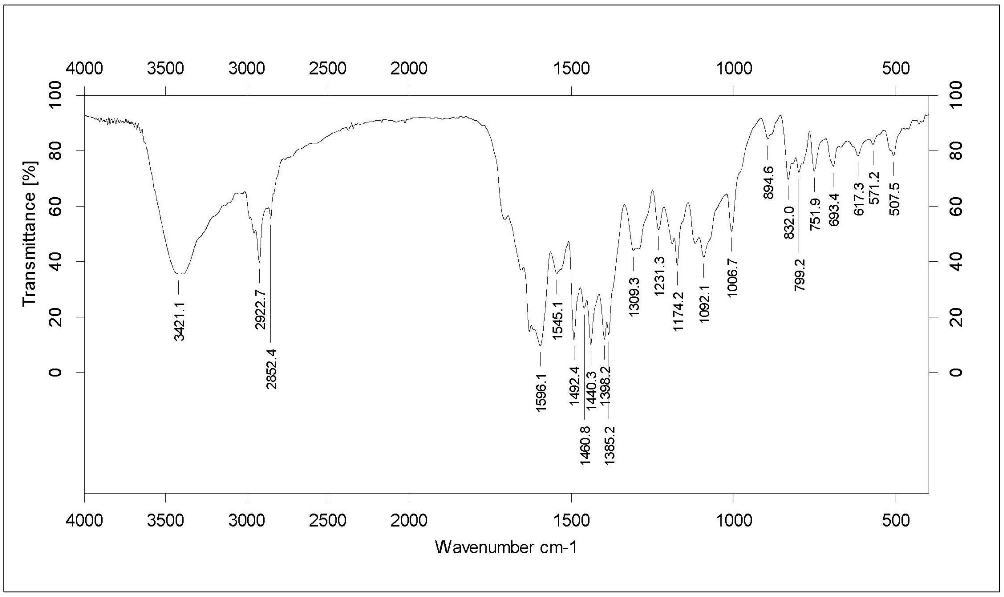

Sample : ZCM-01		Frequency Range : 3999.64 - 400.157	Measured on : 2019-4-12
Technique : Sample form	Resolution : 2	Instrument : EQUINOX55	Sample Scans : 16
Customer : Default	Zerofilling : 2	Acquisition : Double Sided,Forv	

Figure S1. IR of the target compound 7a.

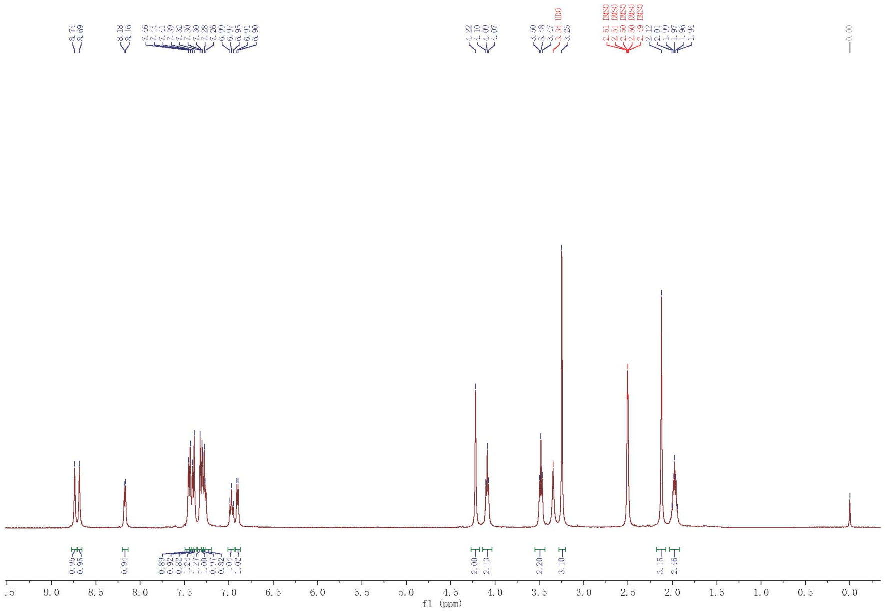

Figure S2. ^{1}H-NMR spectra of the target compound 7a.

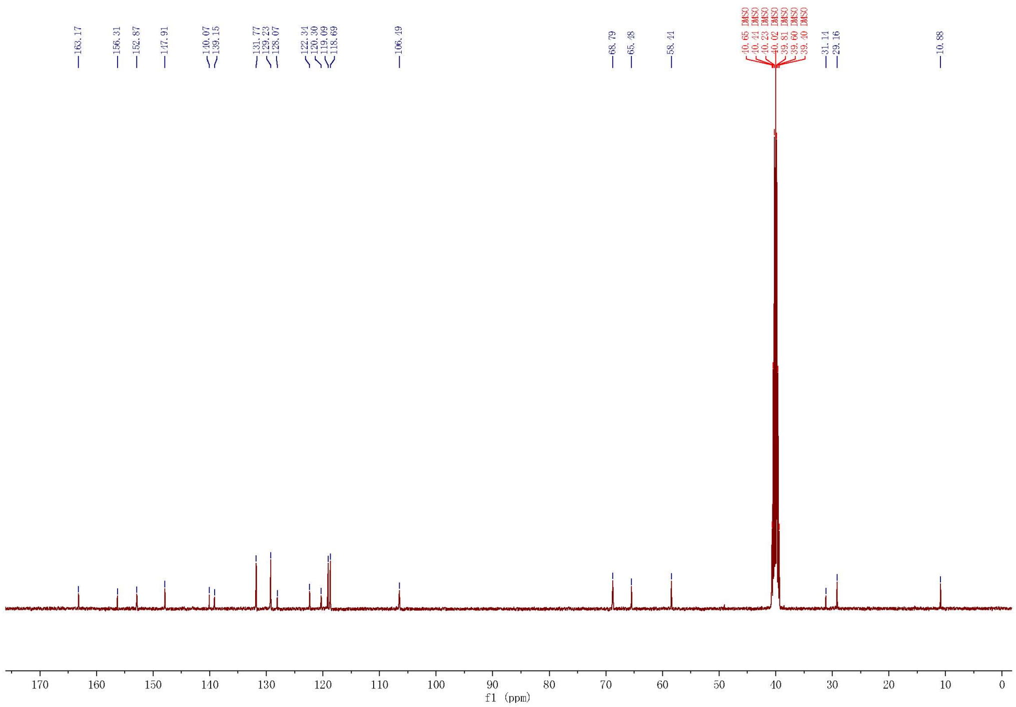

Figure S3. ^{13}C-NMR spectra of the target compound 7a

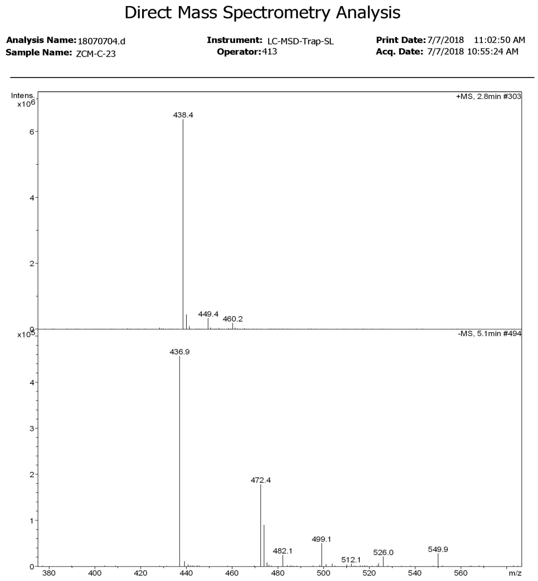

Figure S4. ESI-MS of the target compound 7a

Mass Spectrum SmartFormula Report

Analysis Info

Analysis Name	D:\data\20190521\ZCM-7a pos_000001.d	Acquisition Date	5/21/2019 5:13:48 PM
Method	120-1200_pos_20190520	Operator	
Sample Name		Instrument	solariX
Comment			

Acquisition Parameter

Acquisition Mode	Single MS	Acquired Scans	3	Calibration Date	Mon May 20 10:10:48
Polarity	Positive	No. of Cell Fills	1	Data Acquisition Size	1048576
Broadband Low Mass	118.2 m/z	No. of Laser Shots	500	Data Processing Size (SI)	2097152
Broadband High Mass	1200.0 m/z	Laser Power	20.0 lp	Apodization	Full-Sine
Source Accumulation	0.000 sec	Laser Shot Frequency	0.001 sec		
Ion Accumulation Time	0.050 sec				

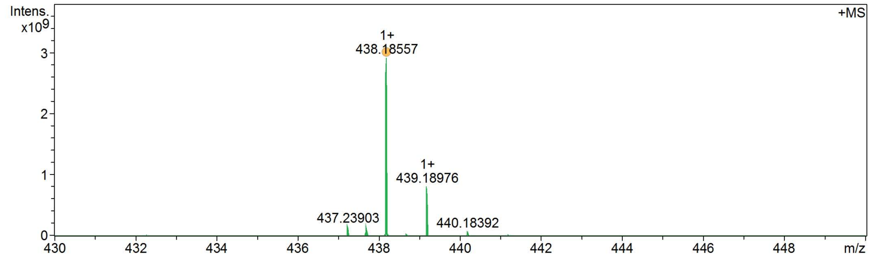

Meas. m/z	#	Ion Formula	Score	m/z	err [ppm]
438.185570	1	C24H28N3O3S	100.00	438.184589	-2.2

Figure S5. HRMS of the target compound 7a

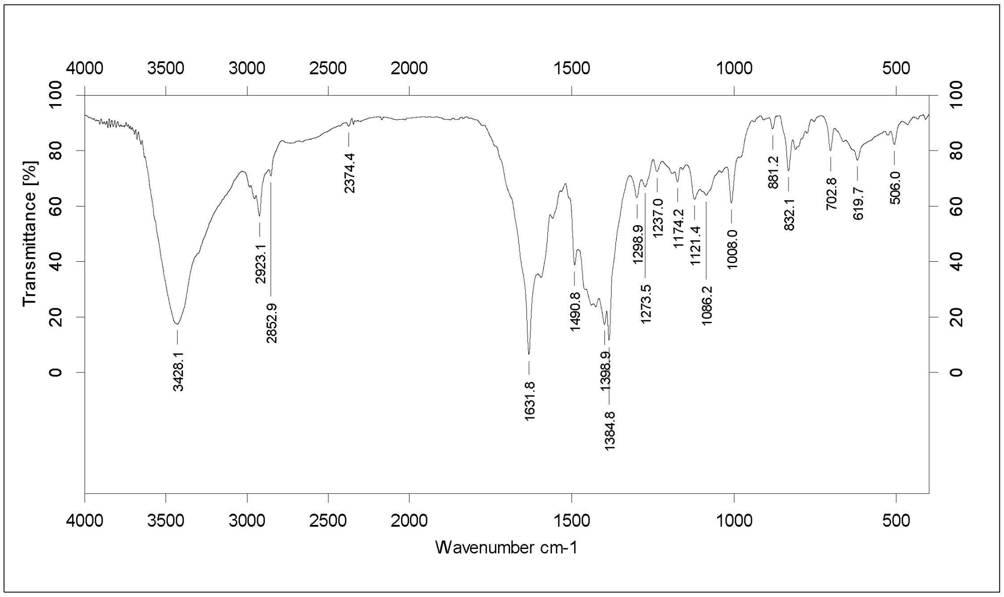

Sample : ZCM-02	Frequency Range : 3999.64 - 400.157		Measured on : 2019-4-12
Technique : Sample form	Resolution : 2	Instrument : EQUINOX55	Sample Scans : 16
Customer : Default	Zerofilling : 2	Acquisition : Double Sided,Forv	

Figure S6. IR of the target compound 7b

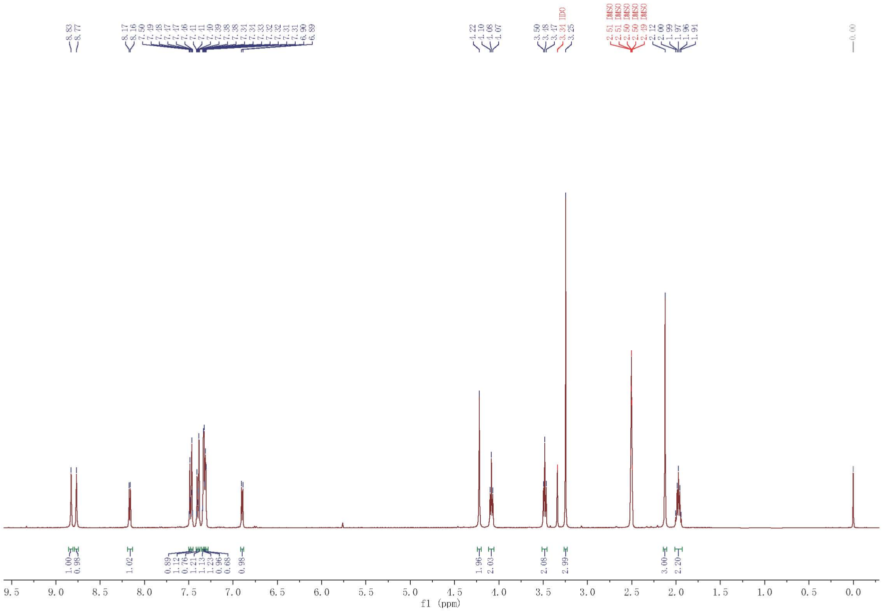

Figure S7. ^{1}H-NMR spectra of the target compound 7b

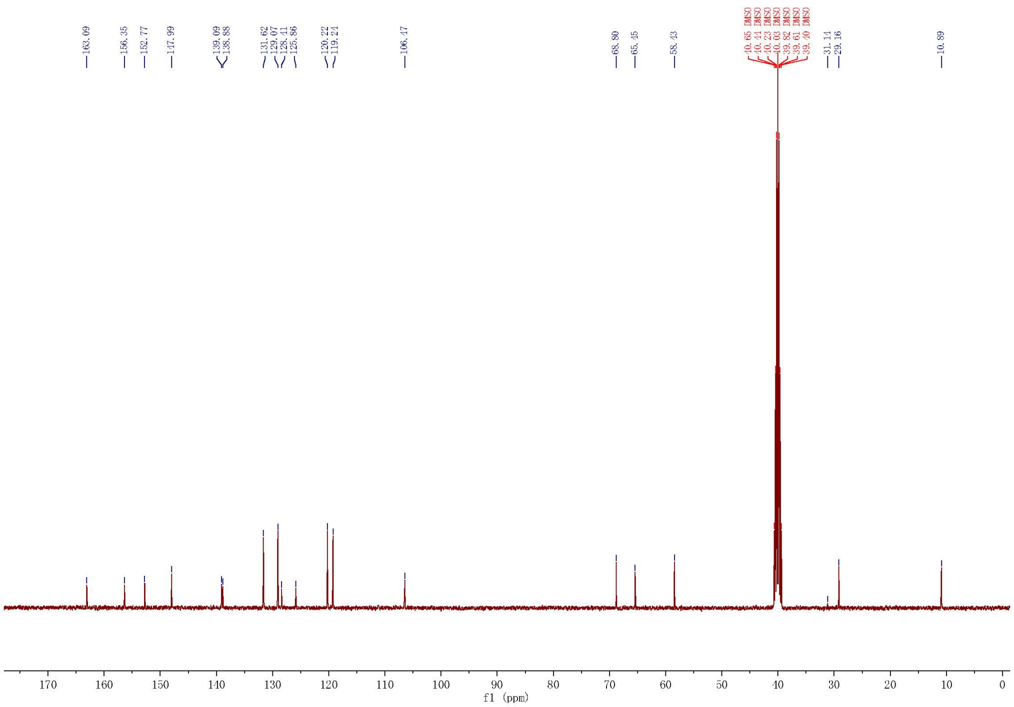

Figure S8. ^{13}C-NMR spectra of the target compound 7b

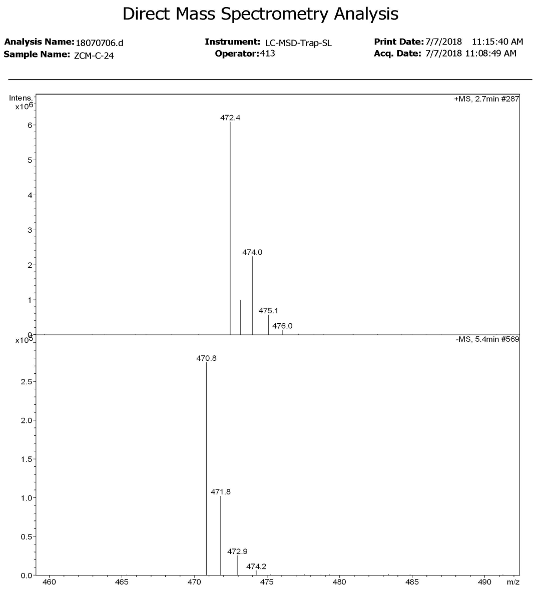

Figure S9. ESI-MS of the target compound 7b

Mass Spectrum SmartFormula Report

Analysis Info

Analysis Name	D:\data\20190521\ZCM-7b POS _000001.d	Acquisition Date	5/21/2019 1:32:30 PM
Method	120-1200_pos_20190520	Operator	
Sample Name		Instrument	solariX
Comment			

Acquisition Parameter

Acquisition Mode	Single MS	Acquired Scans	6	Calibration Date	Mon May 20 10:10:48
Polarity	Positive	No. of Cell Fills	1	Data Acquisition Size	1048576
Broadband Low Mass	118.2 m/z	No. of Laser Shots	500	Data Processing Size (SI)	2097152
Broadband High Mass	1200.0 m/z	Laser Power	20.0 lp	Apodization	Full-Sine
Source Accumulation	0.000 sec	Laser Shot Frequency	0.001 sec		
Ion Accumulation Time	0.050 sec				

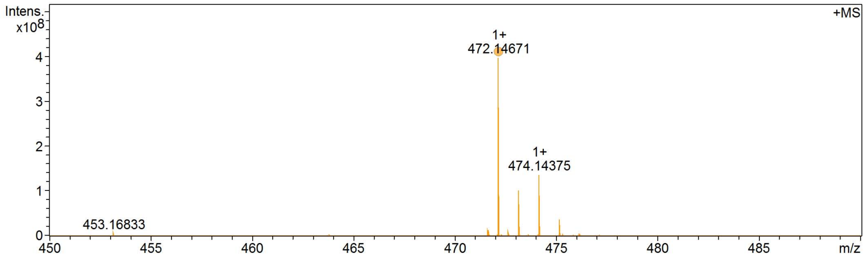

Meas. m/z	#	Ion Formula	Score	m/z	err [ppm]
472.146707	1	C24H27ClN3O3S	100.00	472.145617	-2.3

Bruker Compass DataAnalysis 4.4 By: Shenyang Pharmaceutical University Page 1 of 1

Figure S10. HRMS of the target compound 7b

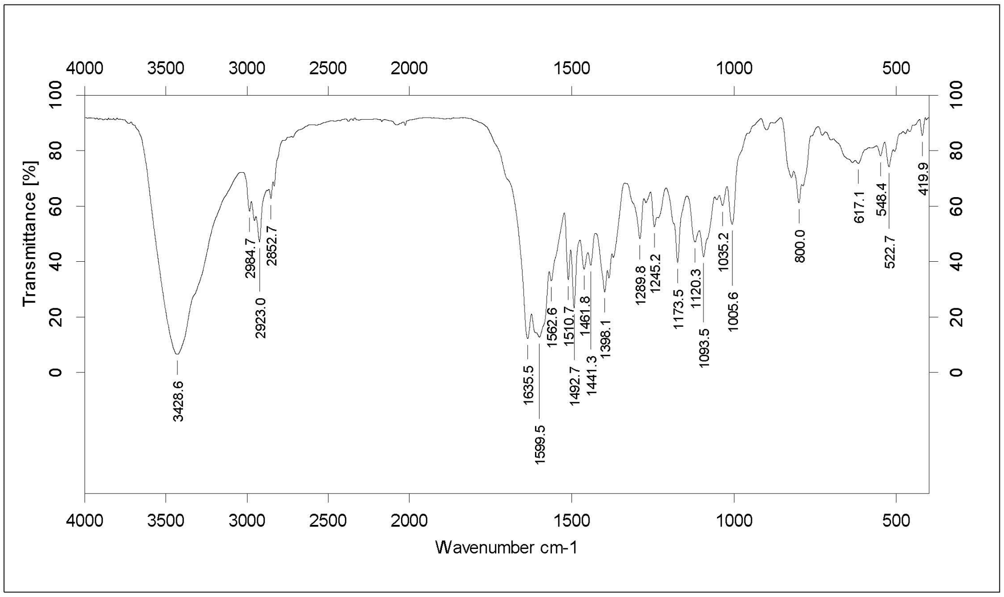

Sample : ZCM-09	Frequency Range : 3999.64 - 400.157	Measured on : 2019-4-12	
Technique : Sample form	Resolution : 2	Instrument : EQUINOX55	Sample Scans : 16
Customer : Default	Zerofilling : 2	Acquisition : Double Sided,Forv	

Figure S11. IR of the target compound 7c

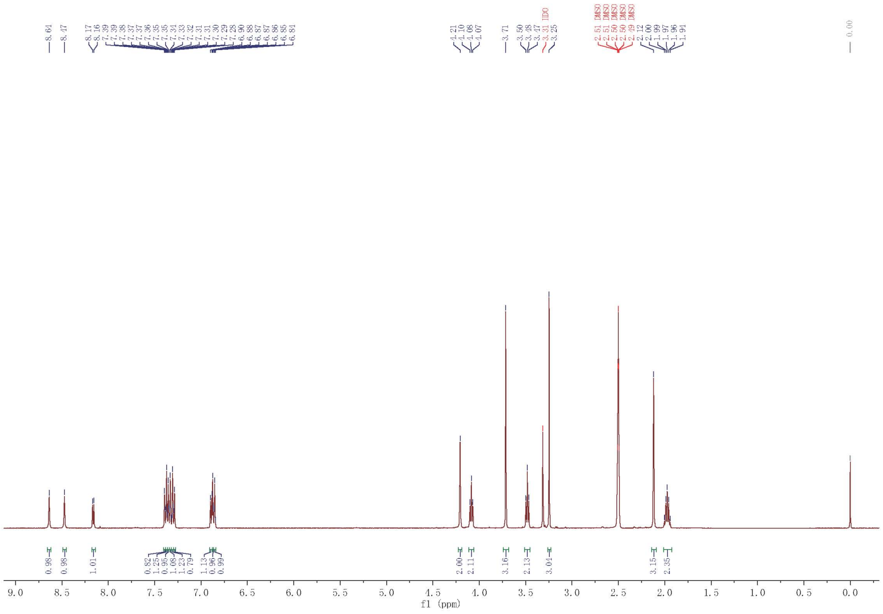

Figure S12. [1] H-NMR spectra of the target compound 7c

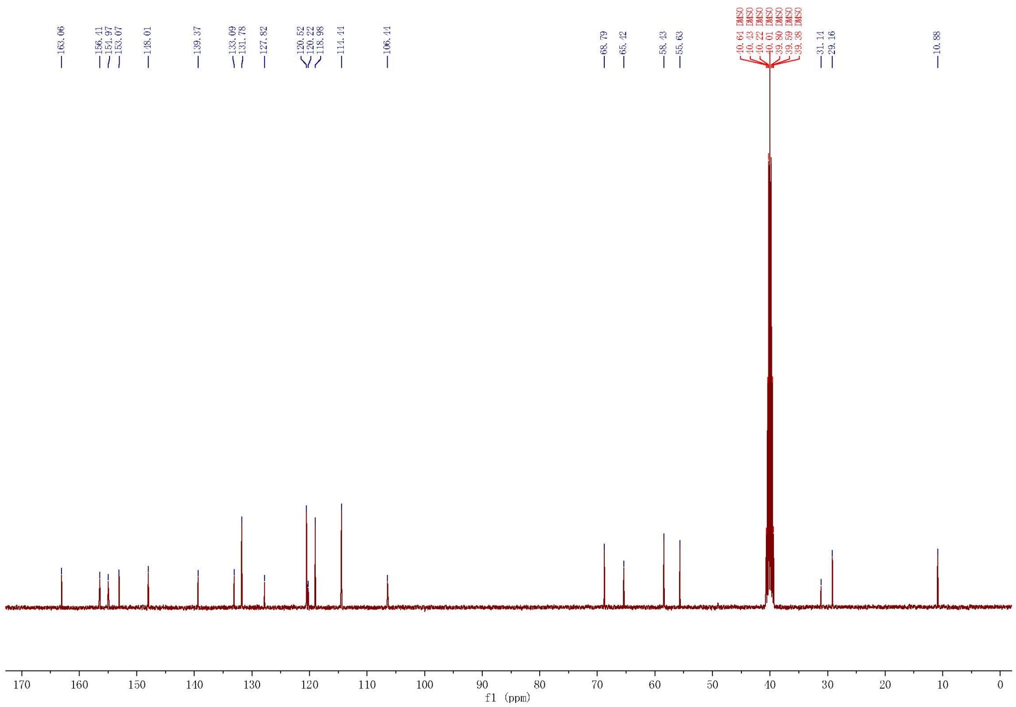

Figure S13. ^{13}C-NMR spectra of the target compound 7c

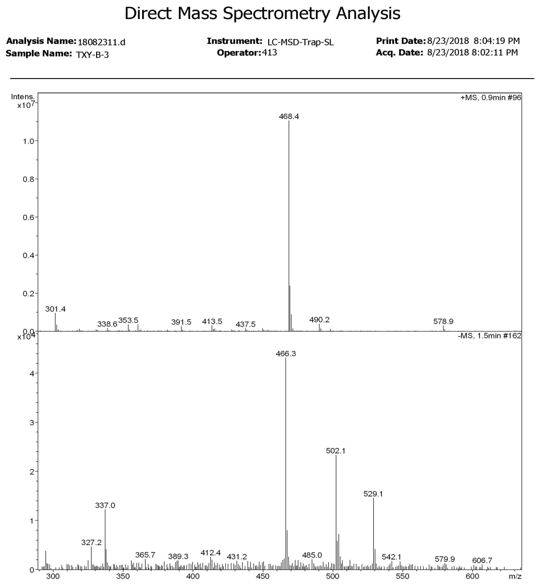

Figure S14. ESI-MS of the target compound 7c

Mass Spectrum SmartFormula Report

Analysis Info

Analysis Name	D:\data\20190521\ZCM-7c-5 POS _000001.d	Acquisition Date	5/21/2019 2:57:20 PM
Method	120-1200_pos_20190520	Operator	
Sample Name		Instrument	solariX
Comment			

Acquisition Parameter

Acquisition Mode	Single MS	Acquired Scans	2	Calibration Date	Mon May 20 10:10:48
Polarity	Positive	No. of Cell Fills	1	Data Acquisition Size	1048576
Broadband Low Mass	118.2 m/z	No. of Laser Shots	500	Data Processing Size (SI)	2097152
Broadband High Mass	1200.0 m/z	Laser Power	20.0 lp	Apodization	Full-Sine
Source Accumulation	0.000 sec	Laser Shot Frequency	0.001 sec		
Ion Accumulation Time	0.050 sec				

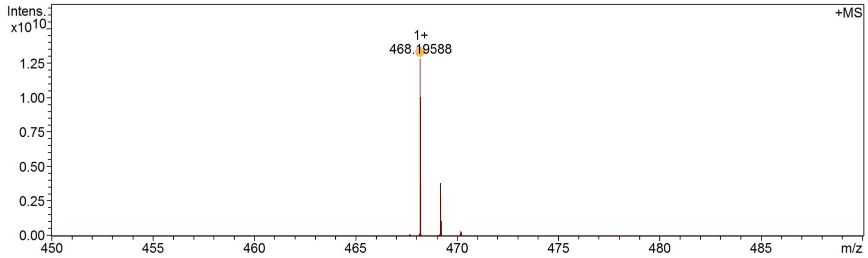

Meas. m/z	#	Ion Formula	Score	m/z	err [ppm]
468.195883	1	C25H30N3O4S	100.00	468.195154	-1.6

Bruker Compass DataAnalysis 4.4 By: Shenyang Pharmaceutical University Page 1 of 1

Figure S15. HRMS of the target compound 7c

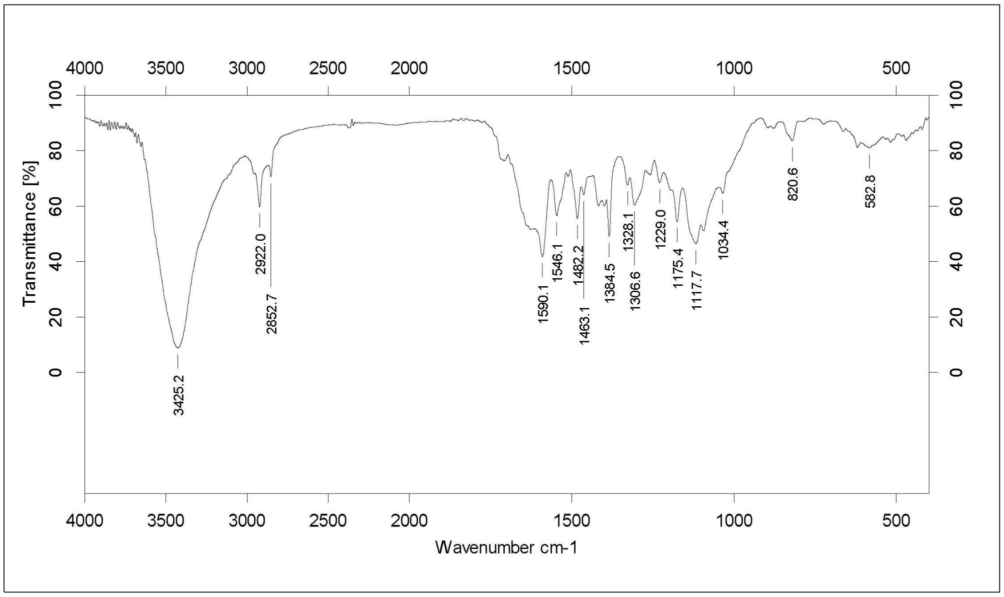

Sample : ZCM-04	Frequency Range : 3999.64 - 400.157	Measured on : 2019-4-25	
Technique : Sample form	Resolution : 2	Instrument : EQUINOX55	Sample Scans : 16
Customer : Default	Zerofilling : 2	Acquisition : Double Sided,Forv	

Figure S16. IR of the target compound 7d

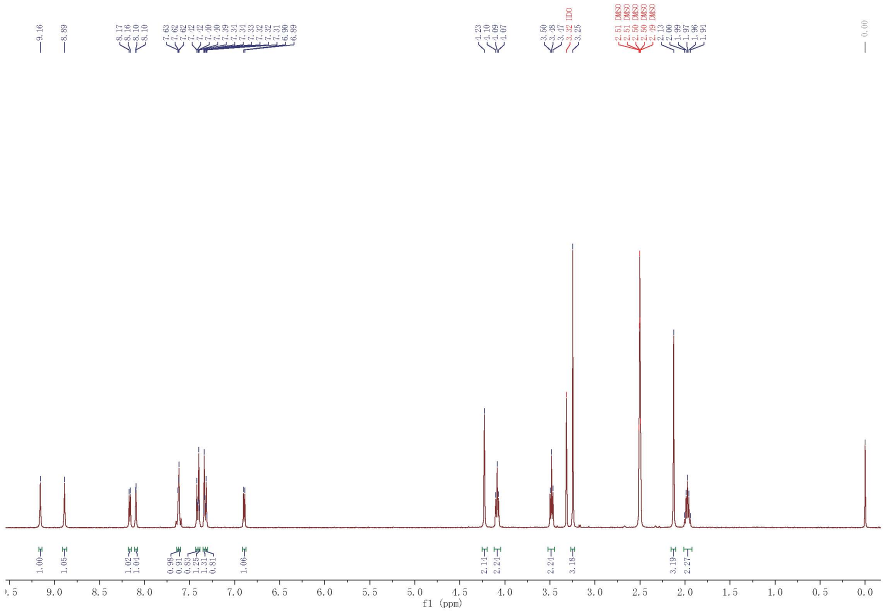

Figure S17. ^{1}H-NMR spectra of the target compound 7d

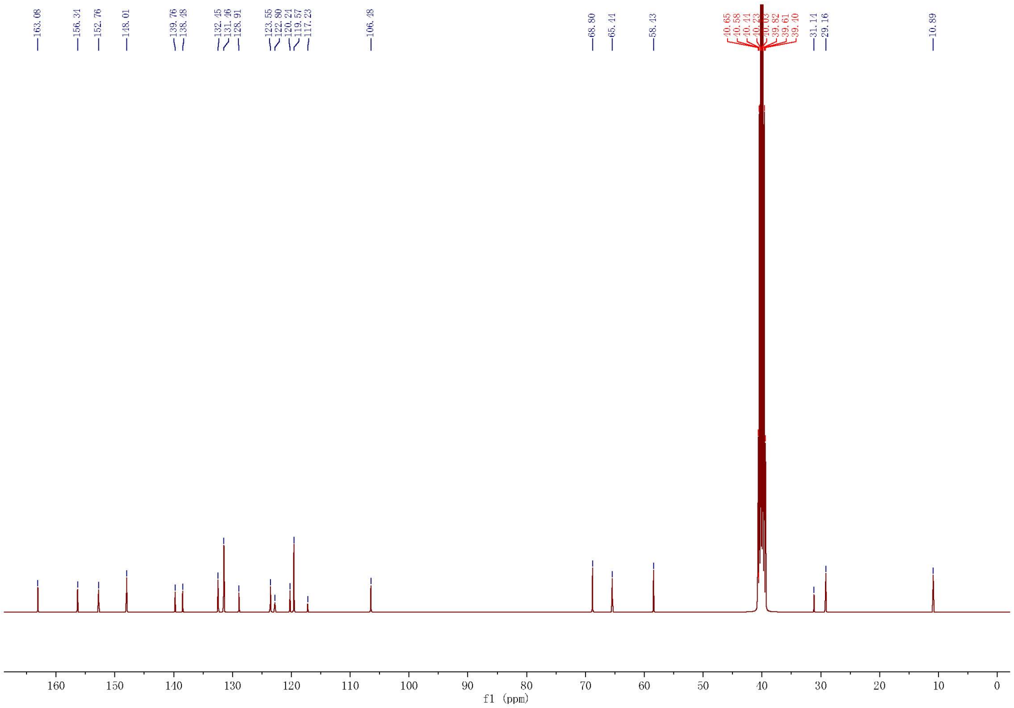

Figure S18. ^{13}C-NMR spectra of the target compound 7d

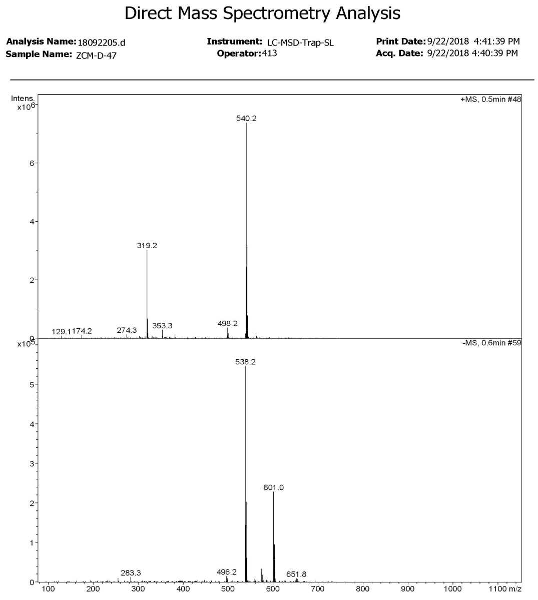

Figure S19. ESI-MS of the target compound 7d

Qualitative Analysis Report

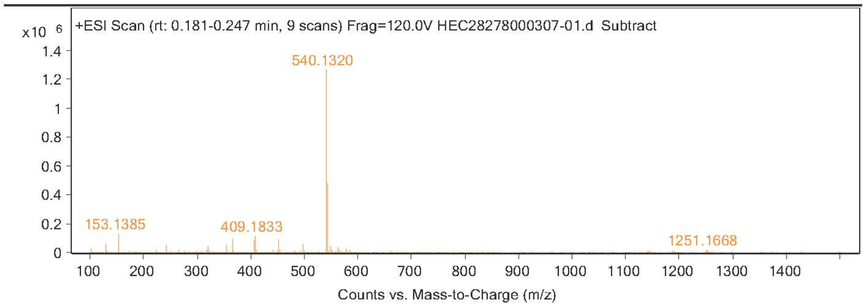

Peak List

m/z	z	Abund
130.1589		53146.53
153.1385	1	123770.56
242.2839		44372.42
355.3678		45426.55
365.1571	1	92442
407.3265	1	85753.79
409.1833	1	106679.53
453.2093	1	90174.73
497.2354		56441.5
540.132	1	1265271.63
541.1356	1	355511.13
541.2596		46171.88
542.1302	1	481802.59
543.1331	1	124155.11
550.0775		37683.75

--- End Of Report ---

Figure S20. HRMS of the target compound 7d

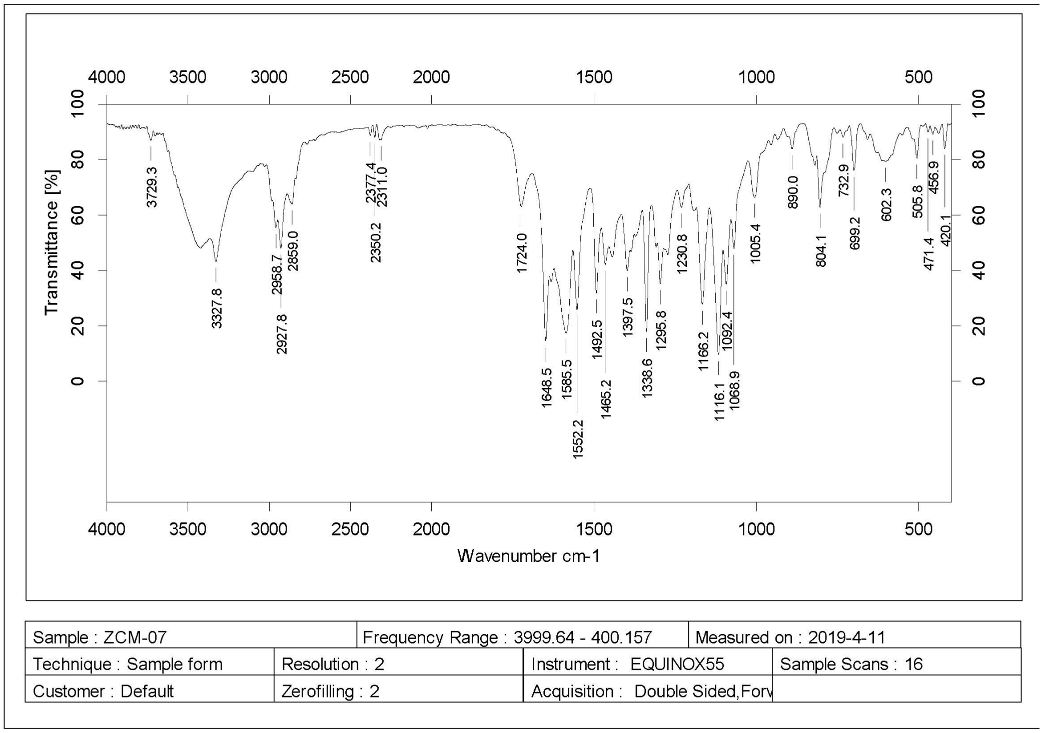

Figure S21. IR of the target compound 7e

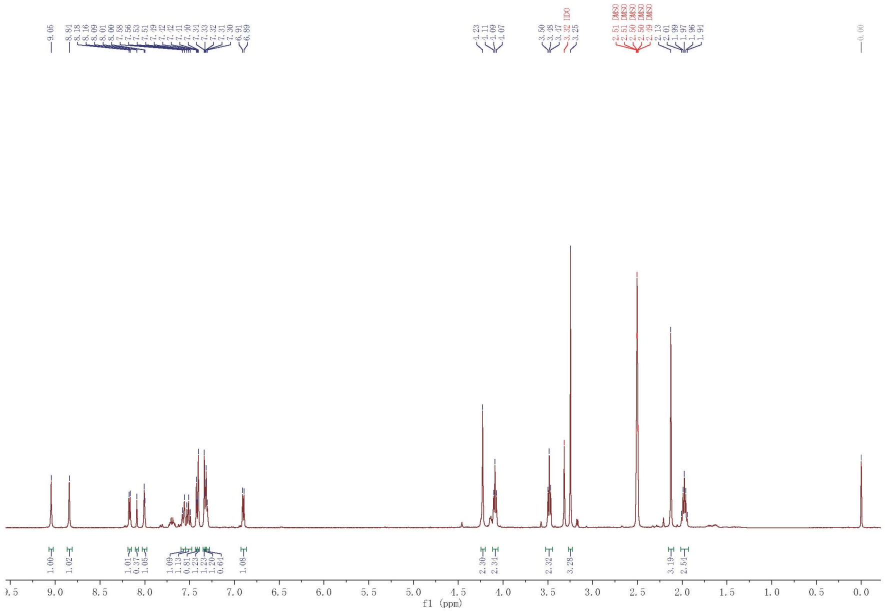

Figure S22. ^{1}H-NMR spectra of the target compound 7e

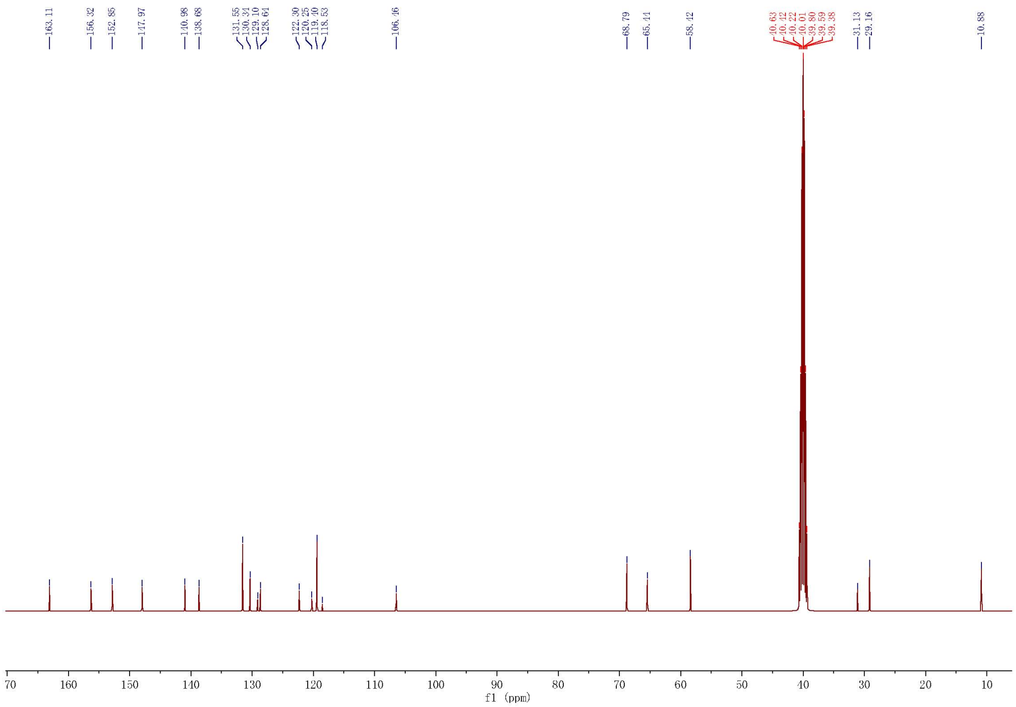

Figure S23. ^{13}C-NMR spectra of the target compound 7e

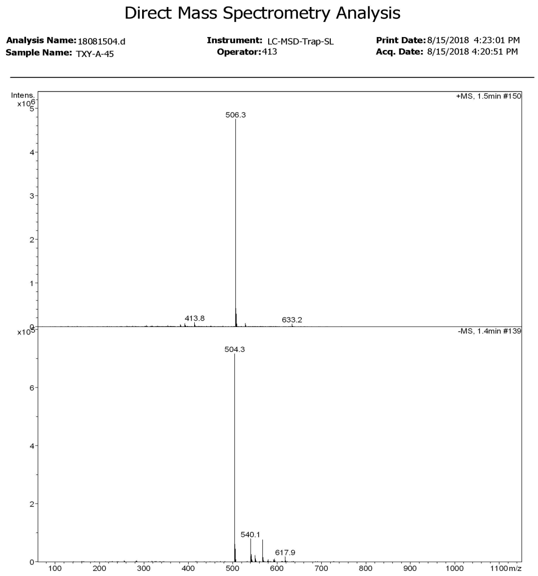

Figure S24. ESI-MS of the target compound 7e

Mass Spectrum SmartFormula Report

Analysis Info

Analysis Name	D:\data\20190521\ZCM-7e-1 POS _000001.d	Acquisition Date	5/21/2019 2:07:35 PM
Method	120-1200_pos_20190520	Operator	
Sample Name		Instrument	solariX
Comment			

Acquisition Parameter

Acquisition Mode	Single MS	Acquired Scans	3	Calibration Date	Mon May 20 10:10:48
Polarity	Positive	No. of Cell Fills	1	Data Acquisition Size	[illegible]
Broadband Low Mass	118.2 m/z	No. of Laser Shots	500	Data Processing Size (SI)	2097152
Broadband High Mass	1200.0 m/z	Laser Power	20.0 lp	Apodization	Full-Sine
Source Accumulation	0.000 sec	Laser Shot Frequency	0.001 sec		
Ion Accumulation Time	0.050 sec				

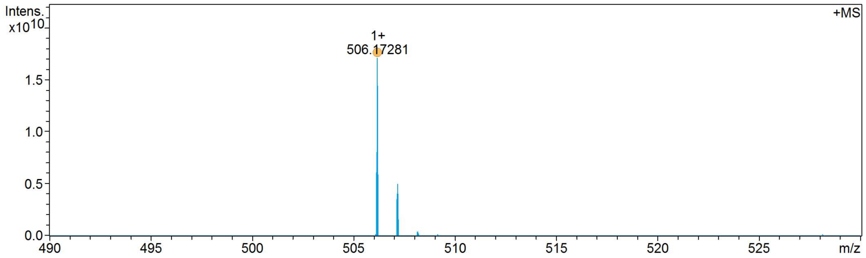

Meas. m/z	#	Ion Formula	Score	m/z	err [ppm]
506.172805	1	C25H27F3N3O3S	100.00	506.171974	-1.6

Bruker Compass DataAnalysis 4.4 By: Shenyang Pharmaceutical University Page 1 of 1

Figure S25. HRMS of the target compound 7e

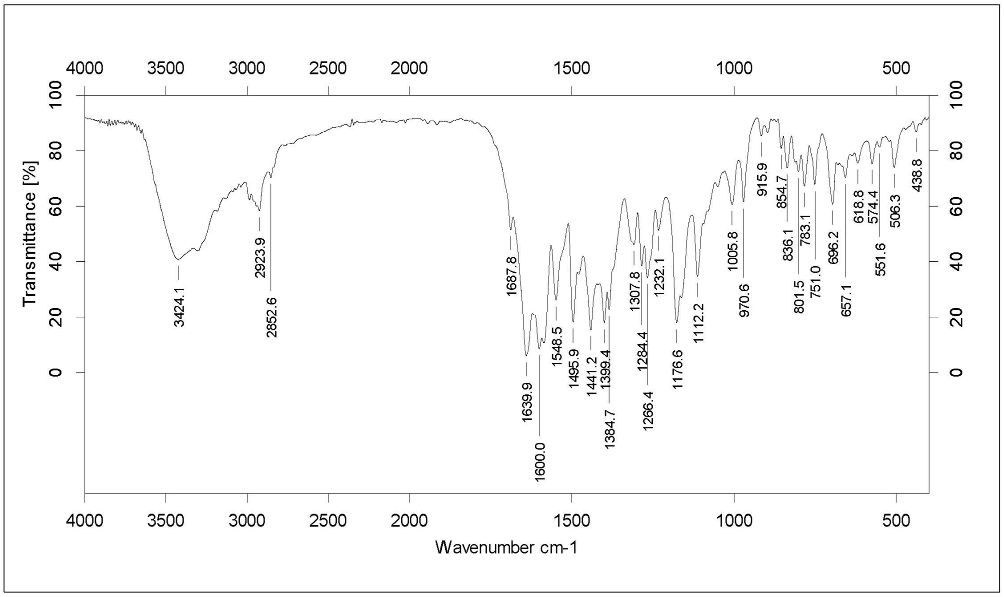

Sample : ZCM-34	Frequency Range : 3999.64 - 400.157		Measured on : 2019-4-12
Technique : Sample form	Resolution : 2	Instrument : EQUINOX55	Sample Scans : 16
Customer : Default	Zerofilling : 2	Acquisition : Double Sided,Forv	

Figure S26. IR of the target compound 7f

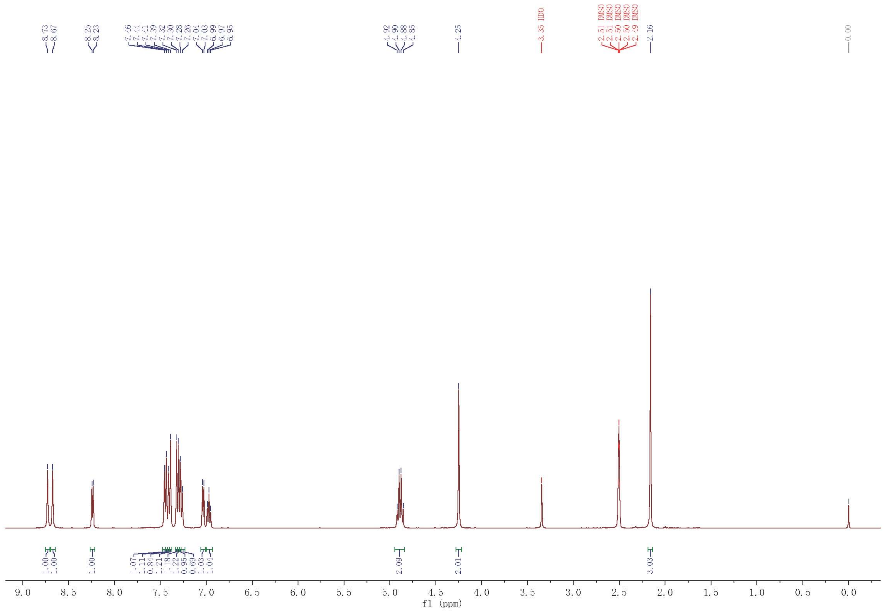

Figure S27. [1] H-NMR spectra of the target compound 7f

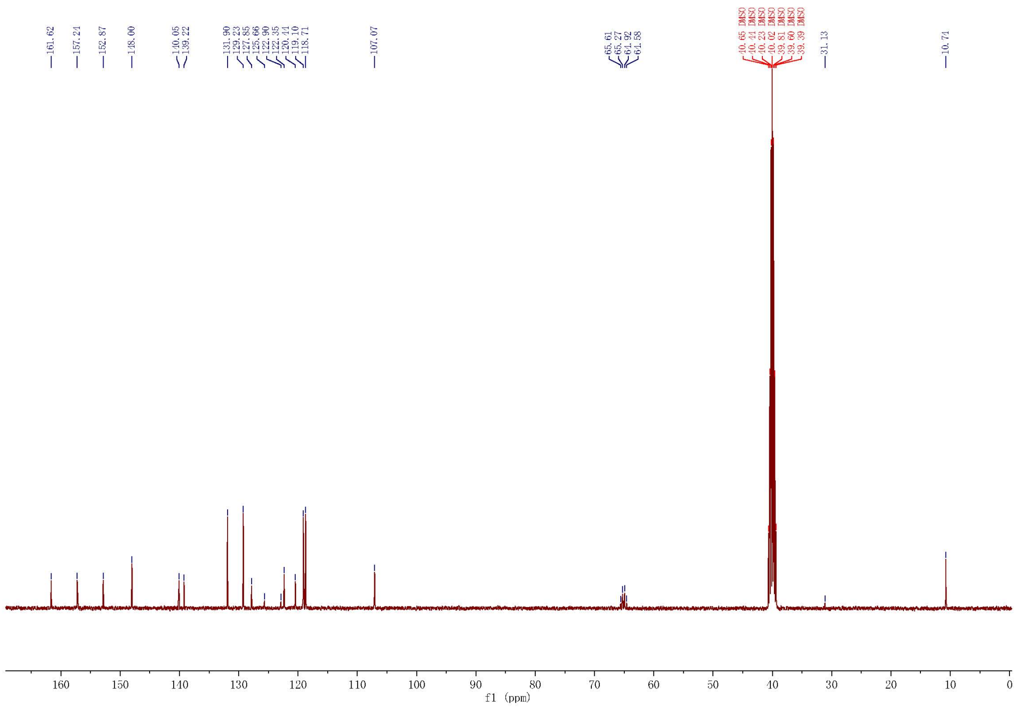

Figure S28. ^{13}C-NMR spectra of the target compound 7f

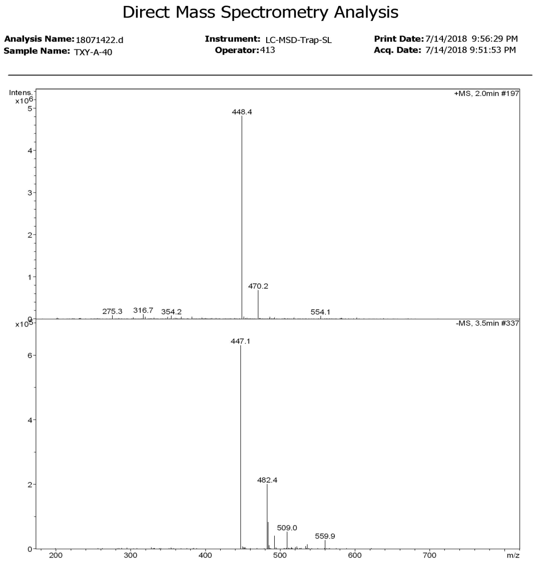

Figure S29. ESI-MS of the target compound 7f

Mass Spectrum SmartFormula Report

Analysis Info

Analysis Name	D:\data\20190521\ZCM-7f-1 POS _000001.d	Acquisition Date	5/21/2019 2:22:18 PM
Method	120-1200_pos_20190520	Operator	
Sample Name		Instrument	solariX
Comment			

Acquisition Parameter

Acquisition Mode	Single MS	Acquired Scans	8	Calibration Date	Mon May 20 10:10:48
Polarity	Positive	No. of Cell Fills	1	Data Acquisition Size	1048576
Broadband Low Mass	118.2 m/z	No. of Laser Shots	500	Data Processing Size (SI)	2097152
Broadband High Mass	1200.0 m/z	Laser Power	20.0 lp	Apodization	Full-Sine
Source Accumulation	0.000 sec	Laser Shot Frequency	0.001 sec		
Ion Accumulation Time	0.050 sec				

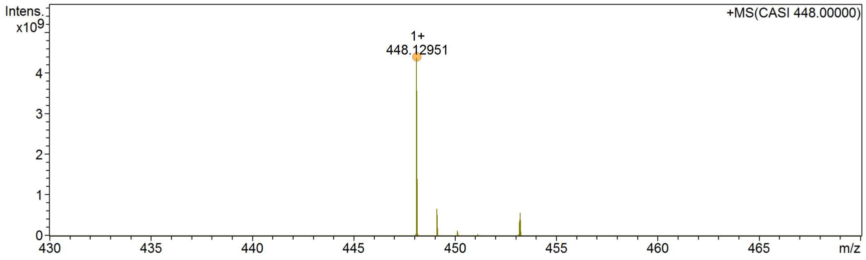

Meas. m/z	#	Ion Formula	Score	m/z	err [ppm]
448.129513	1	C22H21F3N3O2S	78.33	448.130109	1.3

Figure S30. HRMS of the target compound 7f

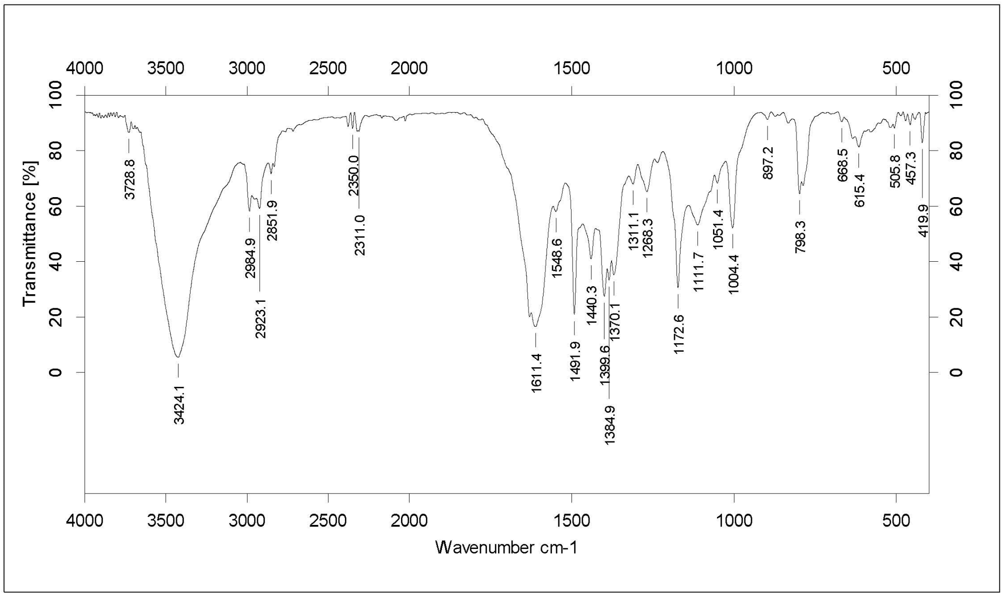

Sample : ZCM-35	Frequency Range : 3999.64 - 400.157		Measured on : 2019-4-11
Technique : Sample form	Resolution : 2	Instrument : EQUINOX55	Sample Scans : 16
Customer : Default	Zerofilling : 2	Acquisition : Double Sided,Forv	

Figure S31. IR of the target compound 7g

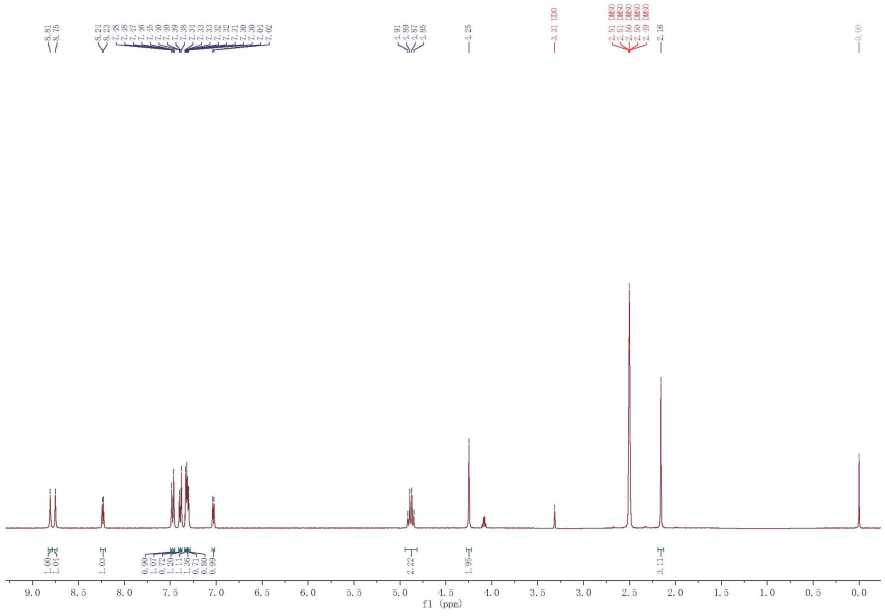

Figure S32. [1] H-NMR spectra of the target compound 7g

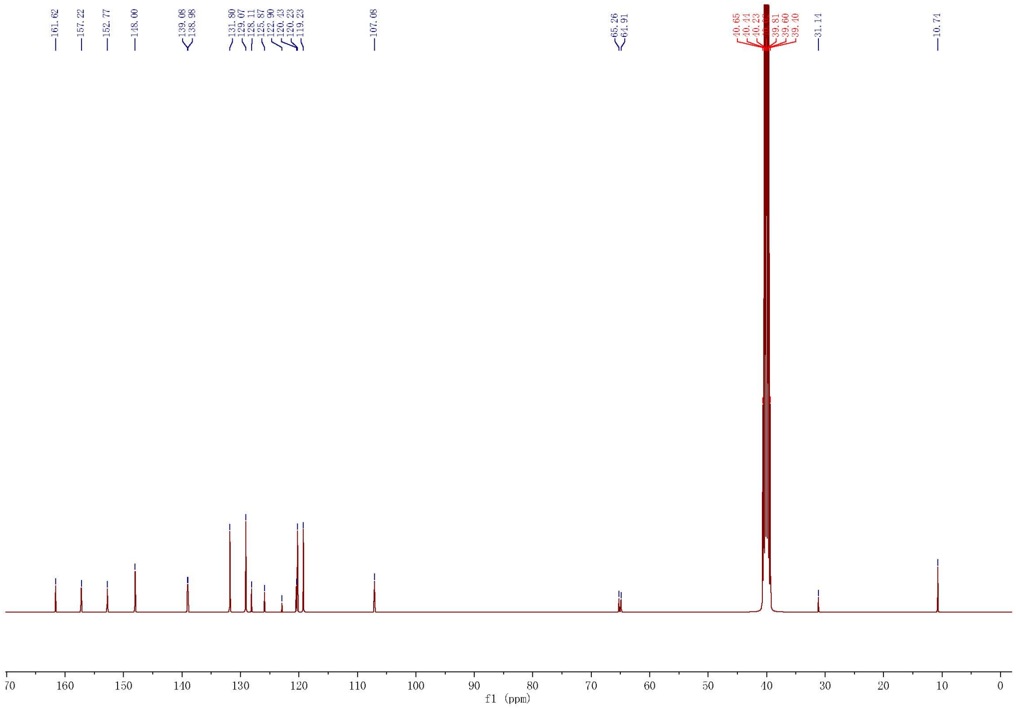

Figure S33. ^{13}C-NMR spectra of the target compound 7g

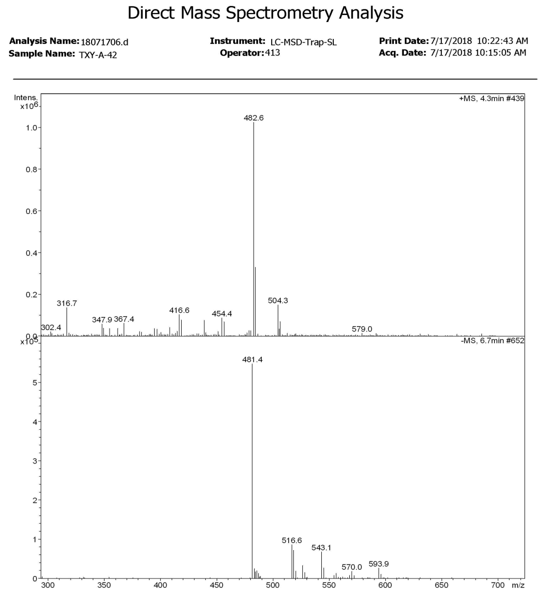

Figure S34. ESI-MS of the target compound 7g

Mass Spectrum SmartFormula Report

Analysis Info

Analysis Name	D:\data\20190521\ZCM-7g pos_000001.d	Acquisition Date	5/21/2019 5:06:07 PM
Method	120-1200_pos_20190520	Operator	
Sample Name		Instrument	solariX
Comment			

Acquisition Parameter

Acquisition Mode	Single MS	Acquired Scans	3	Calibration Date	Mon May 20 10:10:48
Polarity	Positive	No. of Cell Fills	1	Data Acquisition Size	[illegible]
Broadband Low Mass	118.2 m/z	No. of Laser Shots	500	Data Processing Size (SI)	2097152
Broadband High Mass	1200.0 m/z	Laser Power	20.0 lp	Apodization	Full-Sine
Source Accumulation	0.000 sec	Laser Shot Frequency	0.001 sec		
Ion Accumulation Time	0.050 sec				

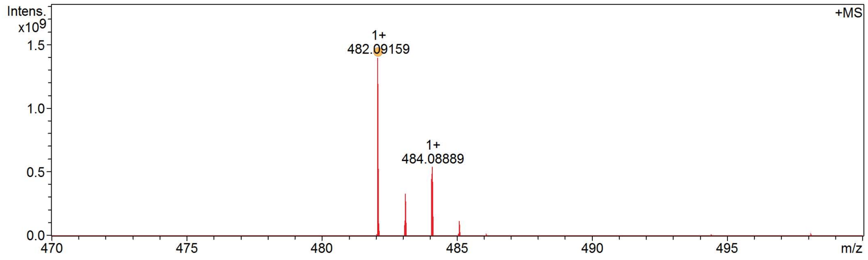

Meas. m/z	#	Ion Formula	Score	m/z	err [ppm]
482.091592	1	C22H20ClF3N3O2S	100.00	482.091137	-0.9

Figure S35. HRMS of the target compound 7g

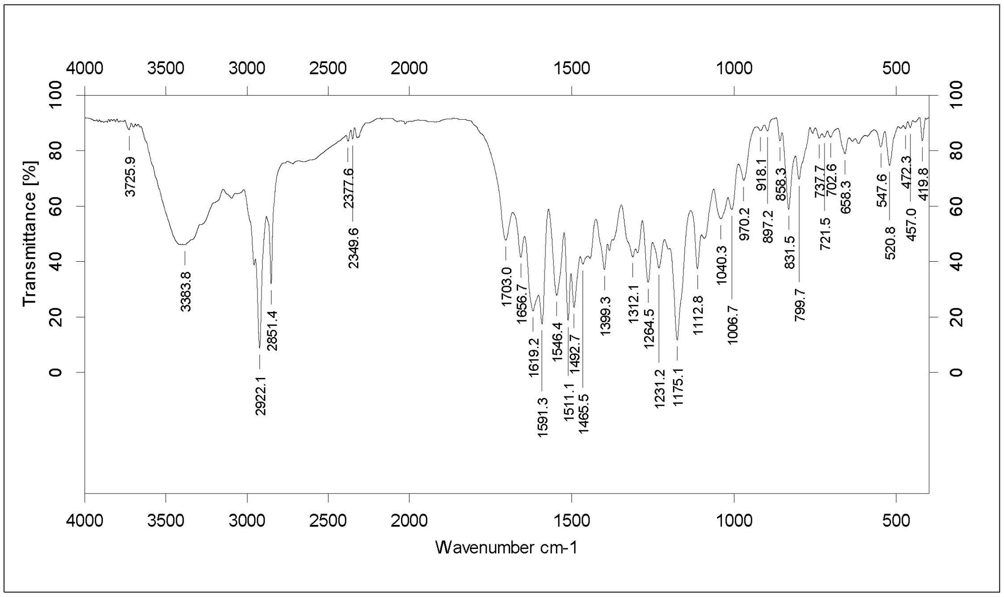

Sample : ZCM-38		Frequency Range : 3999.64 - 400.157	Measured on : 2019-4-11
Technique : Sample form	Resolution : 2	Instrument : EQUINOX55	Sample Scans : 16
Customer : Default	Zerofilling : 2	Acquisition : Double Sided,Forv	

Figure S36. IR of the target compound 7h

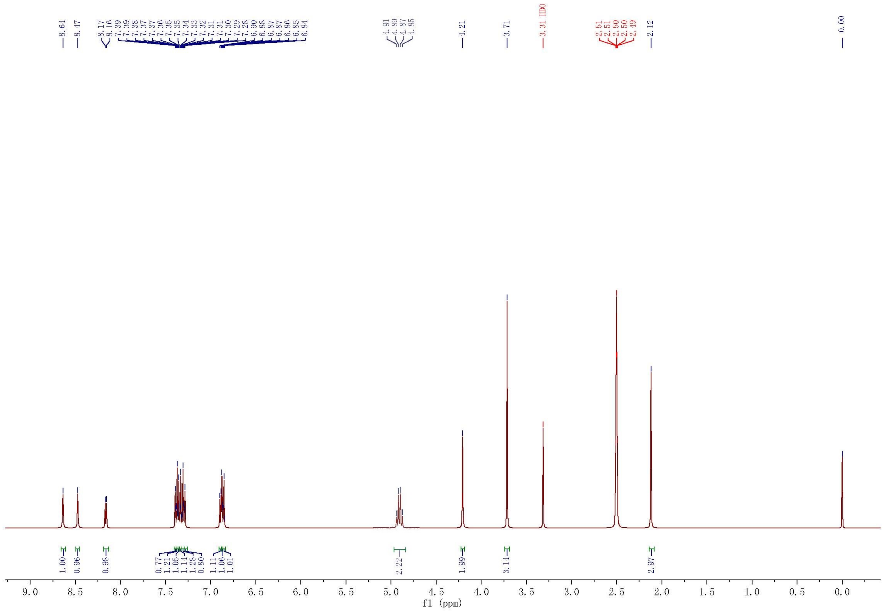

Figure S37. ^{1}H-NMR spectra of the target compound 7h

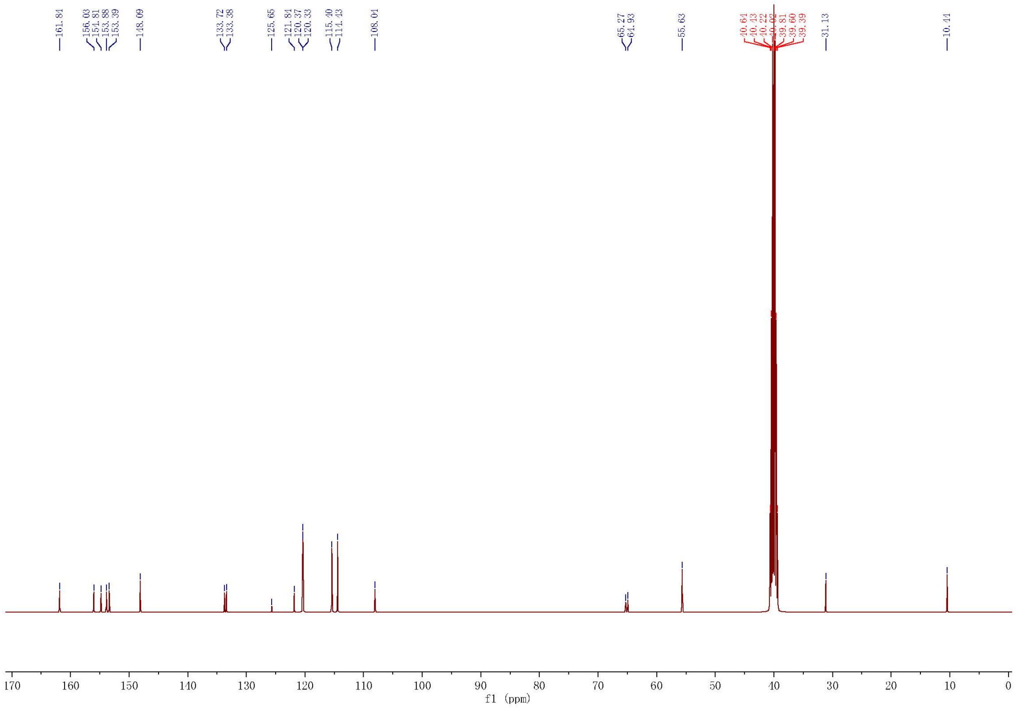

Figure S38. ^{13}C-NMR spectra of the target compound 7h

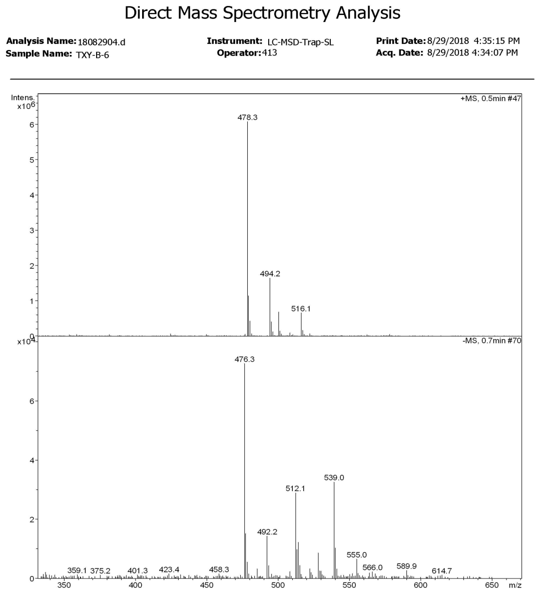

Figure S39. ESI-MS of the target compound 7h

Mass Spectrum SmartFormula Report

Analysis Info

Analysis Name	D:\data\20190521\ZCM-7h pos_000001.d	Acquisition Date	5/21/2019 5:26:21 PM
Method	120-1200_pos_20190520	Operator	
Sample Name		Instrument	solariX
Comment			

Acquisition Parameter

Acquisition Mode	Single MS	Acquired Scans	4	Calibration Date	Mon May 20 10:10:48
Polarity	Positive	No. of Cell Fills	1	Data Acquisition Size	1048576
Broadband Low Mass	118.2 m/z	No. of Laser Shots	500	Data Processing Size (SI)	2097152
Broadband High Mass	1200.0 m/z	Laser Power	20.0 lp	Apodization	Full-Sine
Source Accumulation	0.000 sec	Laser Shot Frequency	0.001 sec		
Ion Accumulation Time	0.050 sec				

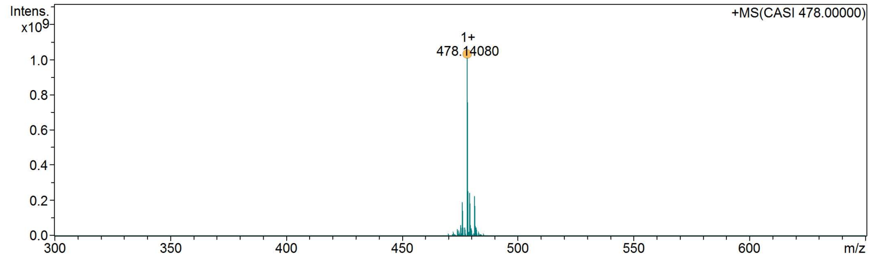

Meas. m/z	#	Ion Formula	Score	m/z	err [ppm]
478.140802	1	C23H23F3N3O3S	100.00	478.140674	-0.3

Bruker Compass DataAnalysis 4.4 By: Shenyang Pharmaceutical University Page 1 of 1

Figure S40. HRMS of the target compound 7h

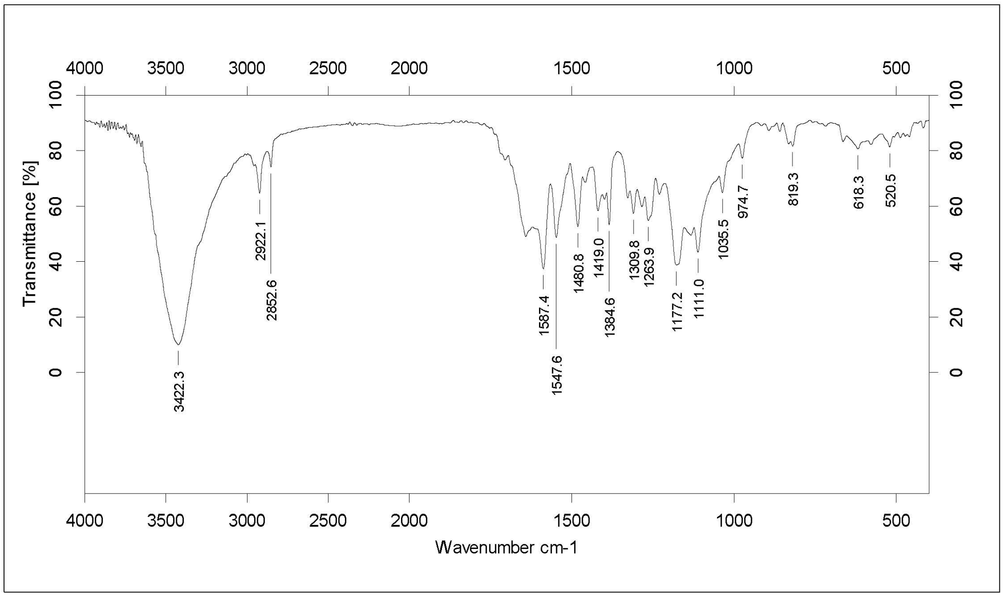

Sample : ZCM-31	Frequency Range : 3999.64 - 400.157	Measured on : 2019-4-25	
Technique : Sample form	Resolution : 2	Instrument : EQUINOX55	Sample Scans : 16
Customer : Default	Zerofilling : 2	Acquisition : Double Sided,Forv	

Figure S41. IR of the target compound 7i

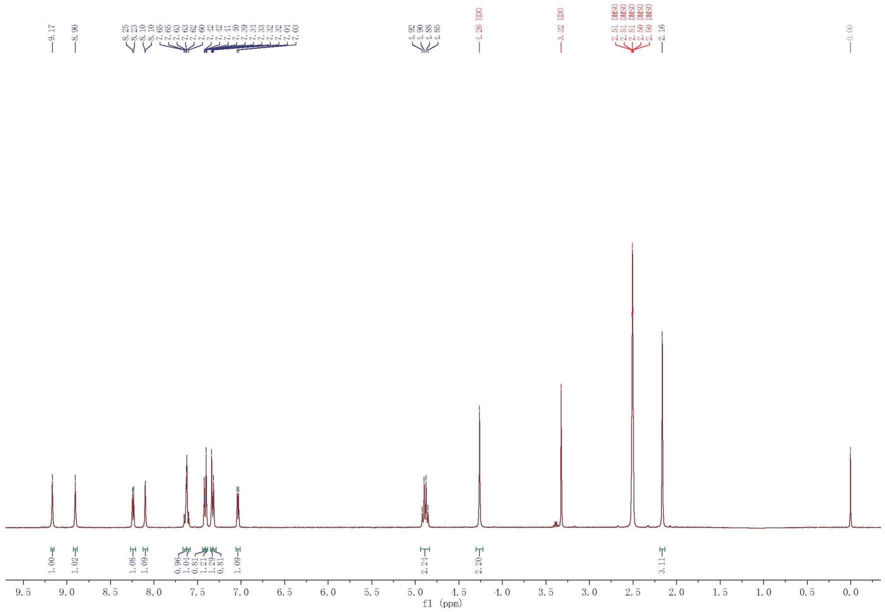

Figure S42. [1] H-NMR spectra of the target compound 7i

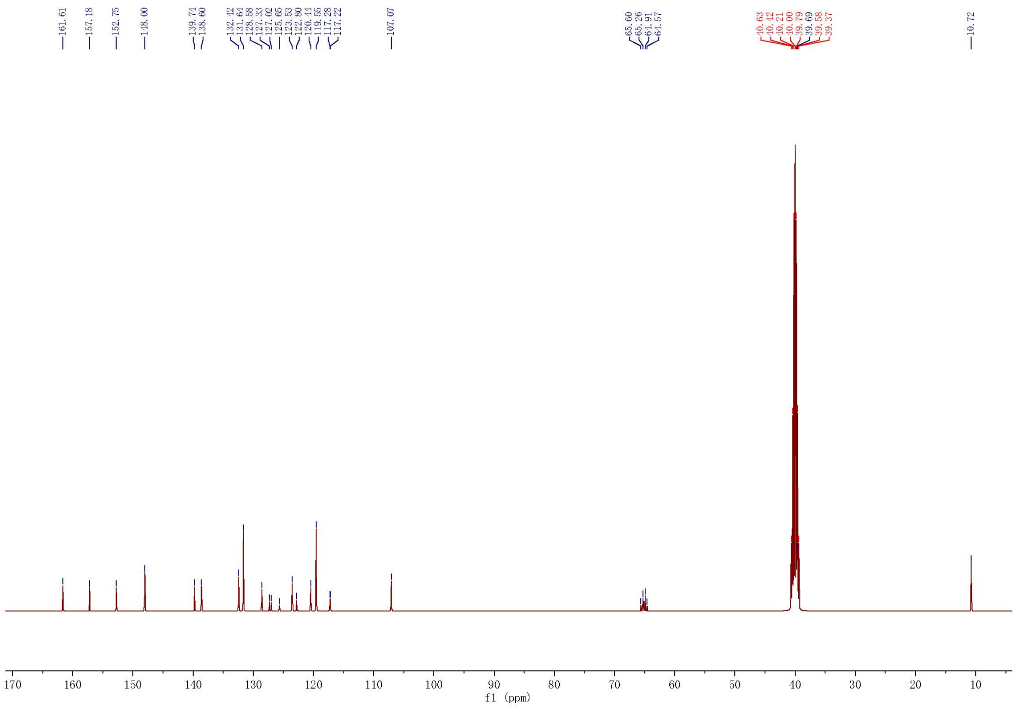

Figure S43. ^{13}C-NMR spectra of the target compound 7i

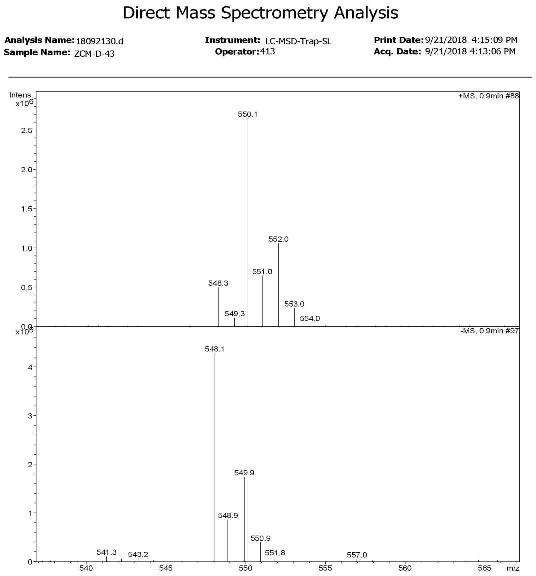

Figure S44. ESI-MS of the target compound 7i

Qualitative Analysis Report

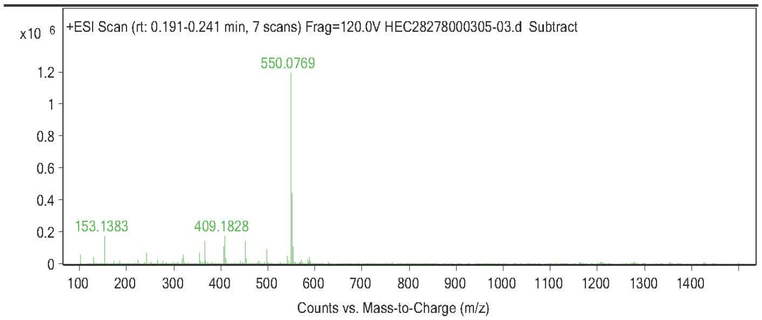

Peak List

m/z	z	Abund
102.1275		52568.8
153.1383	1	172961.95
242.2836	1	70670.22
321.1304		55923.26
355.3673	1	64694.79
365.1567	1	143638.58
407.326	1	104844.37
409.1828	1	174102.91
453.209	1	144233.97
497.2348	1	91380.95
541.2606		48594.79
550.0769	1	1195728.25
551.0805	1	308412.81
552.0751	1	445135.81
553.0779	1	106889.23

--- End Of Report ---

Agilent Technologies Page 2 of 2 Printed at 8:54 AM on 11-Mar-2019

Figure S45. HRMS of the target compound 7i

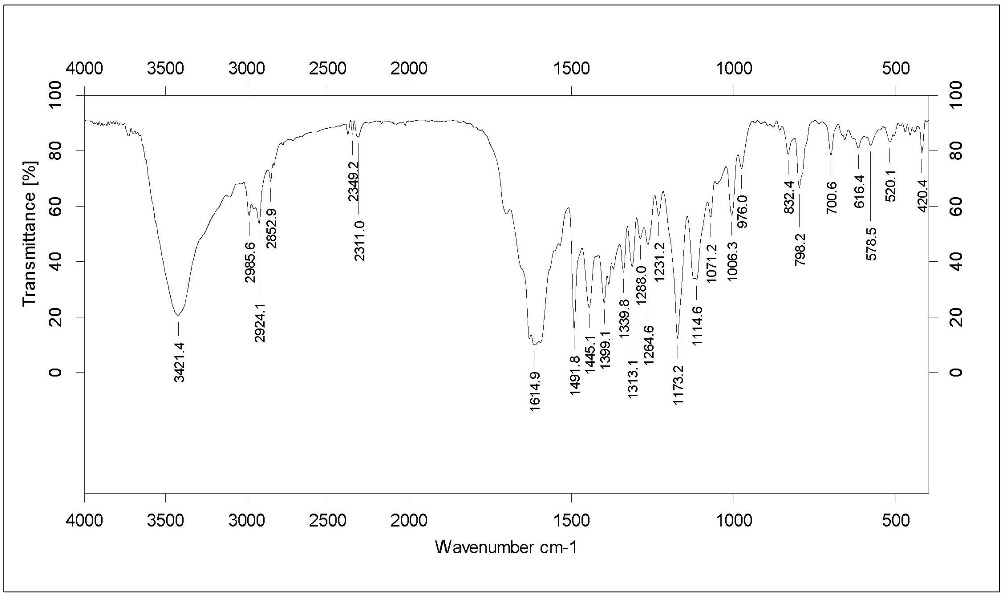

Sample : ZCM-36	Frequency Range : 3999.64 - 400.157	Measured on : 2019-4-11	
Technique : Sample form	Resolution : 2	Instrument : EQUINOX55	Sample Scans : 16
Customer : Default	Zerofilling : 2	Acquisition : Double Sided,Forv	

Figure S46. IR of the target compound 7j

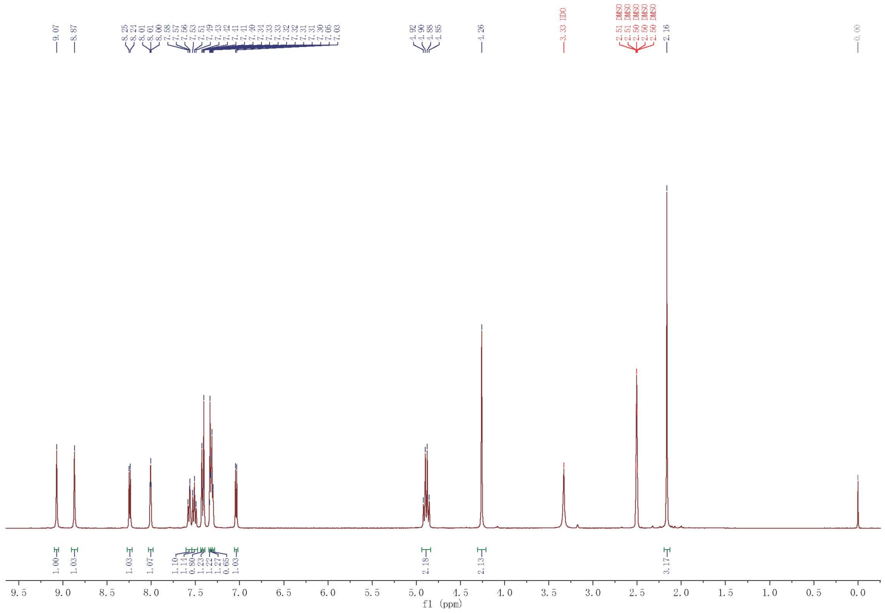

Figure S47. [1] H-NMR spectra of the target compound 7j

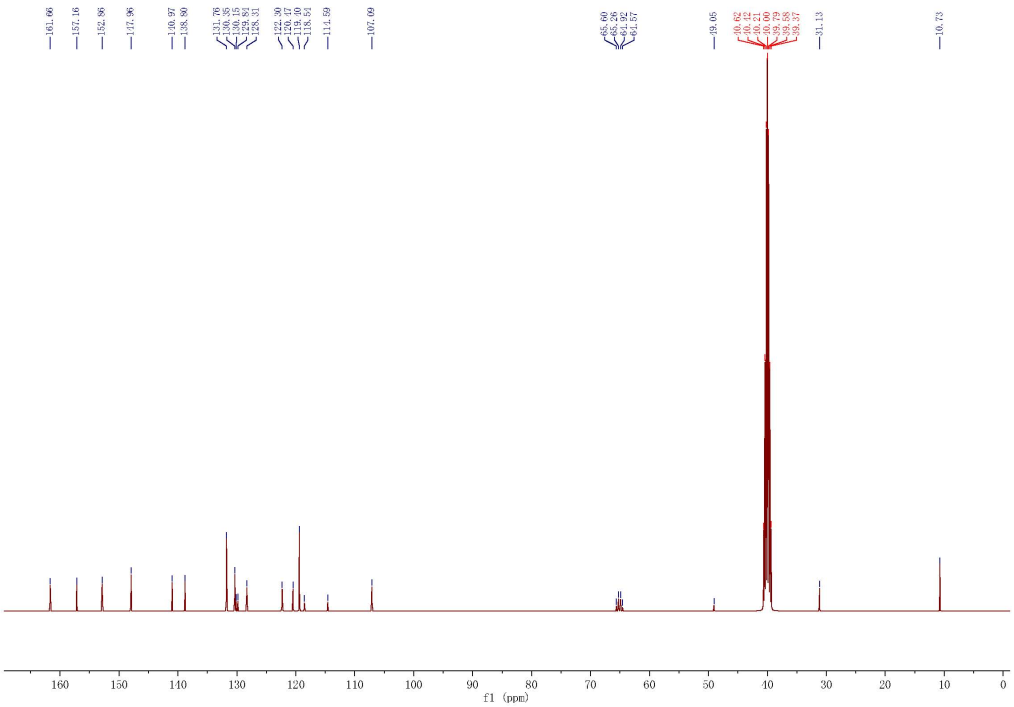

Figure S48. ^{13}C-NMR spectra of the target compound 7j

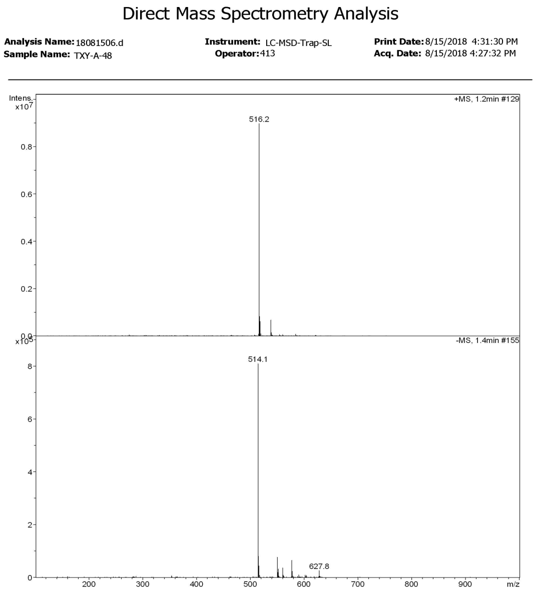

Figure S49. ESI-MS of the target compound 7j

Mass Spectrum SmartFormula Report

Analysis Info

Analysis Name	D:\data\20190521\ZCM-7j POS _000001.d	Acquisition Date	5/21/2019 2:32:56 PM
Method	120-1200_pos_20190520	Operator	
Sample Name		Instrument	solariX
Comment			

Acquisition Parameter

Acquisition Mode	Single MS	Acquired Scans	3	Calibration Date	Mon May 20 10:10:48
Polarity	Positive	No. of Cell Fills	1	Data Acquisition Size	1048576
Broadband Low Mass	118.2 m/z	No. of Laser Shots	500	Data Processing Size (SI)	2097152
Broadband High Mass	1200.0 m/z	Laser Power	20.0 lp	Apodization	Full-Sine
Source Accumulation	0.000 sec	Laser Shot Frequency	0.001 sec		
Ion Accumulation Time	0.050 sec				

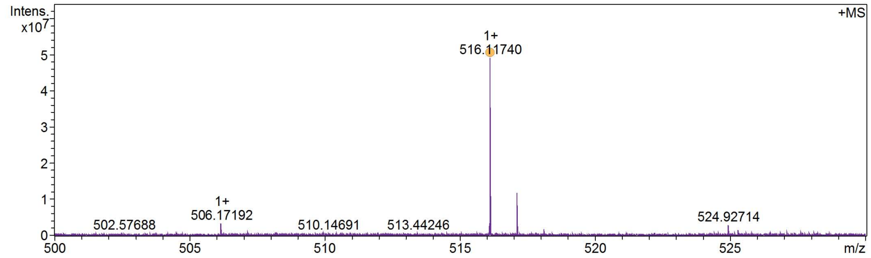

Meas. m/z	#	Ion Formula	Score	m/z	err [ppm]
516.117400	1	C23H20F6N3O2S	100.00	516.117494	0.2

Figure S50. HRMS of the target compound 7j

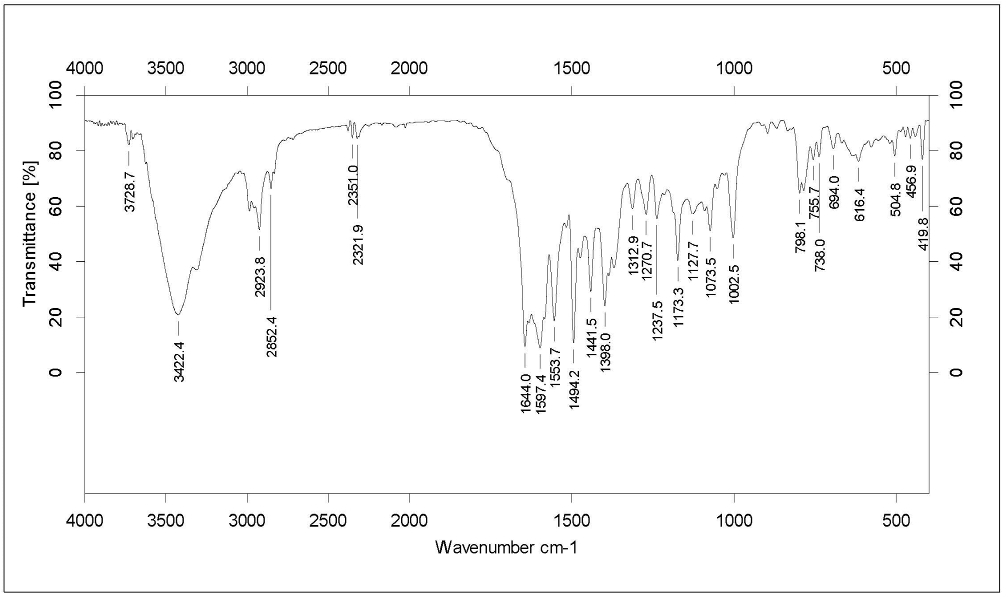

Sample : ZCM-26	Frequency Range : 3999.64 - 400.157	Measured on : 2019-4-11	
Technique : Sample form	Resolution : 2	Instrument : EQUINOX55	Sample Scans : 16
Customer : Default	Zerofilling : 2	Acquisition : Double Sided,Forv	

Figure S51. IR of the target compound 7k

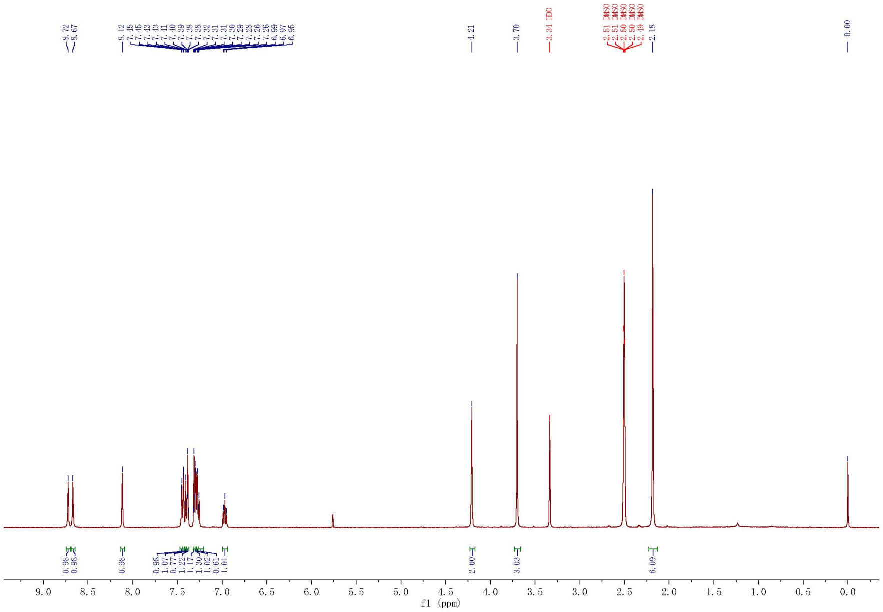

Figure S52. ^{1}H-NMR spectra of the target compound 7k

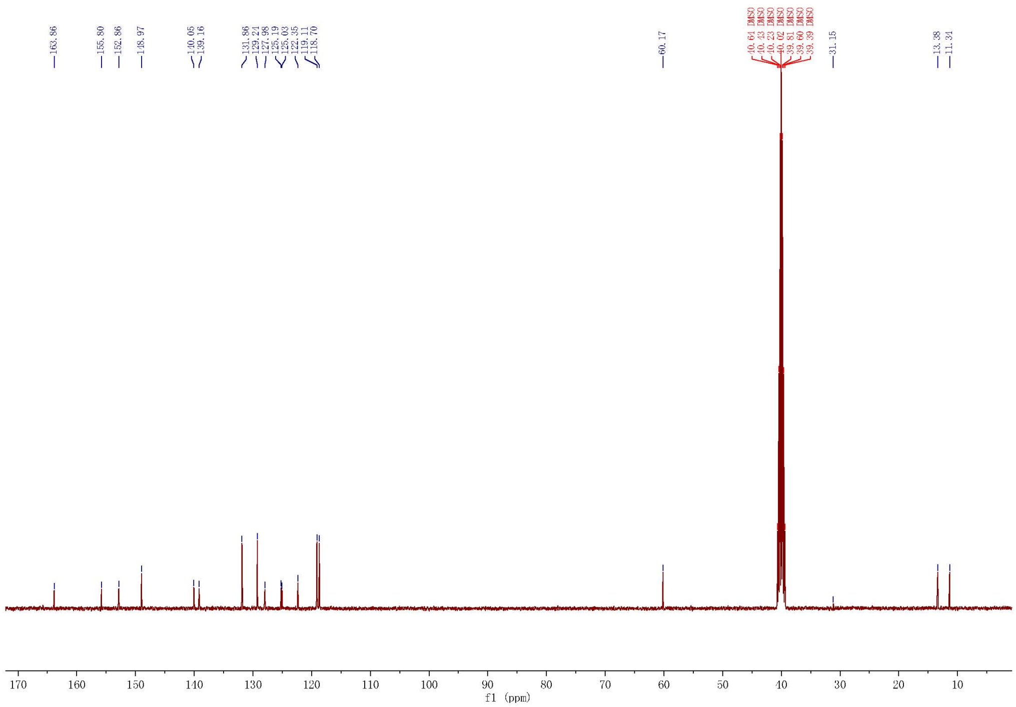

Figure S53. ^{13}C-NMR spectra of the target compound 7k

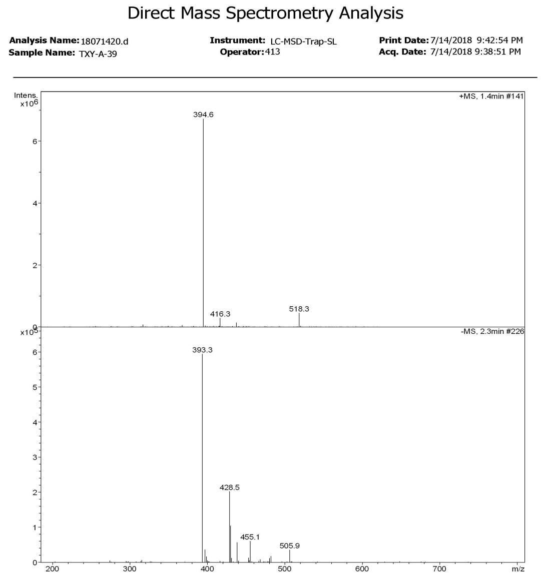

Figure S54. ESI-MS of the target compound 7k

Mass Spectrum SmartFormula Report

Analysis Info

Analysis Name	D:\data\20190521\ZCM-7k pos_000005.d	Acquisition Date	5/21/2019 5:39:10 PM
Method	120-1200_pos_20190520	Operator	
Sample Name		Instrument	solariX
Comment			

Acquisition Parameter

Acquisition Mode	Single MS	Acquired Scans	2	Calibration Date	Mon May 20 10:10:48
Polarity	Positive	No. of Cell Fills	1	Data Acquisition Size	1048576
Broadband Low Mass	118.2 m/z	No. of Laser Shots	500	Data Processing Size (SI)	2097152
Broadband High Mass	1200.0 m/z	Laser Power	20.0 lp	Apodization	Full-Sine
Source Accumulation	0.000 sec	Laser Shot Frequency	0.001 sec		
Ion Accumulation Time	0.050 sec				

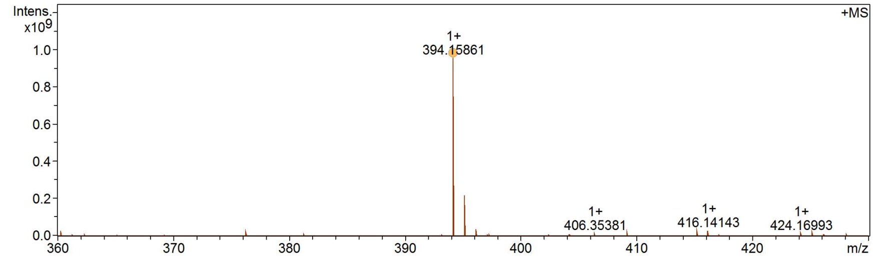

Meas. m/z	#	Ion Formula	Score	m/z	err [ppm]
394.158614	1	C22H24N3O2S	11.41	394.158374	-0.6

Bruker Compass DataAnalysis 4.4 By: Shenyang Pharmaceutical University Page 1 of 1

Figure S55. HRMS of the target compound 7k

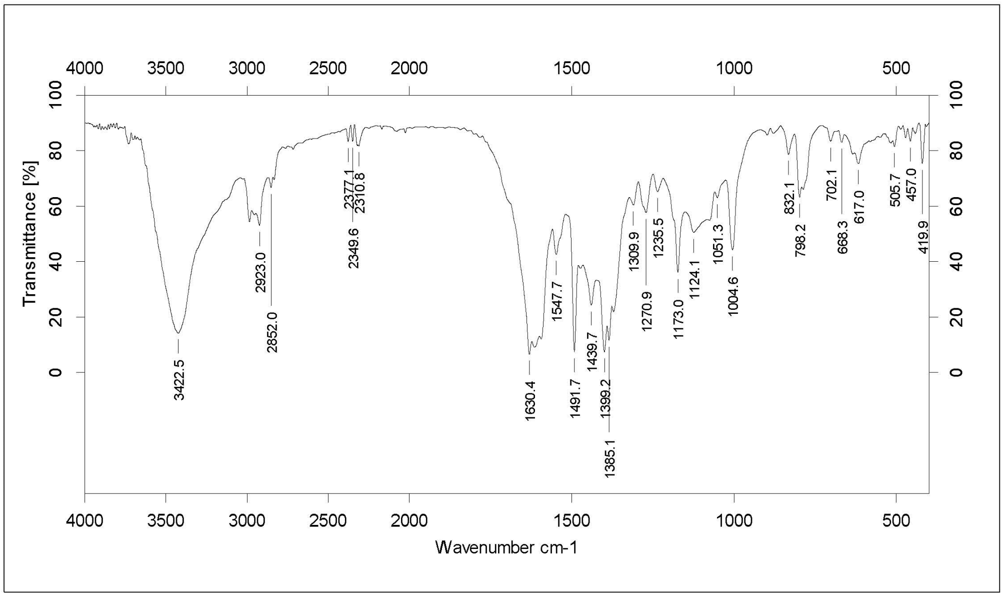

Sample : ZCM-27	Frequency Range : 3999.64 - 400.157	Measured on : 2019-4-11	
Technique : Sample form	Resolution : 2	Instrument : EQUINOX55	Sample Scans : 16
Customer : Default	Zerofilling : 2	Acquisition : Double Sided,Forv	

Figure S56. IR of the target compound 7l

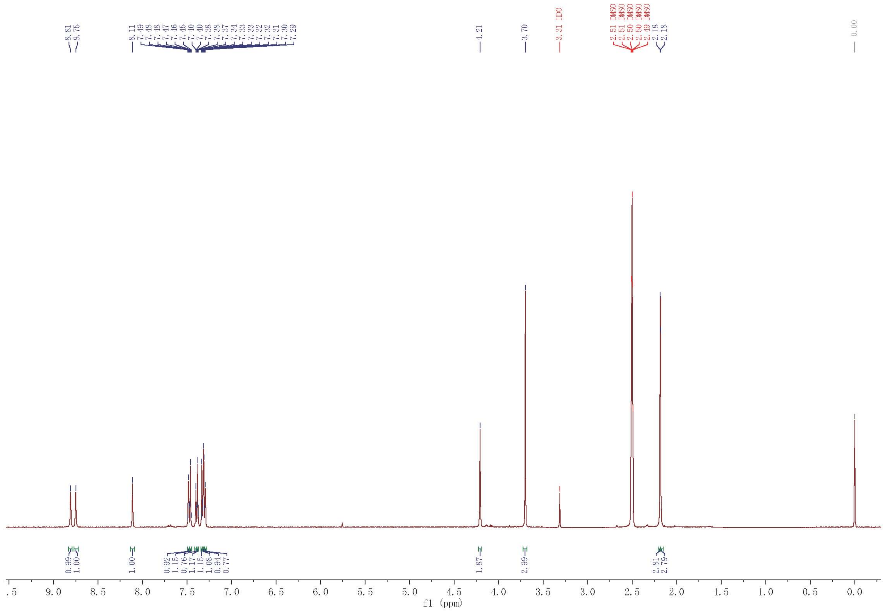

Figure S57. ^{1}H-NMR spectra of the target compound 7l

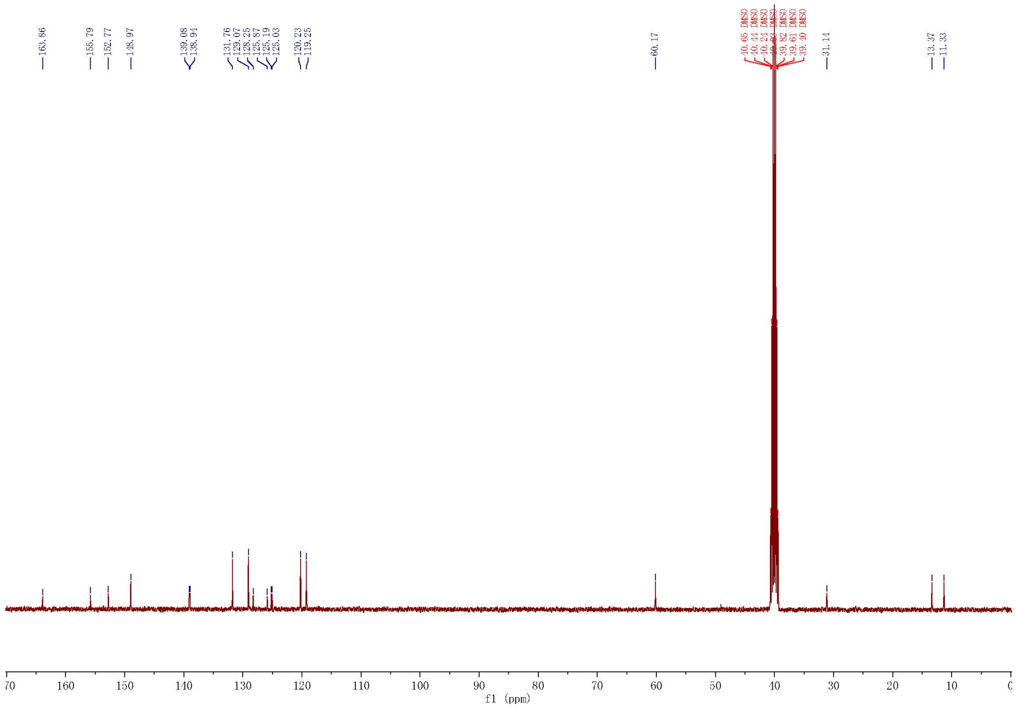

Figure S58. ^{13}C-NMR spectra of the target compound 7l

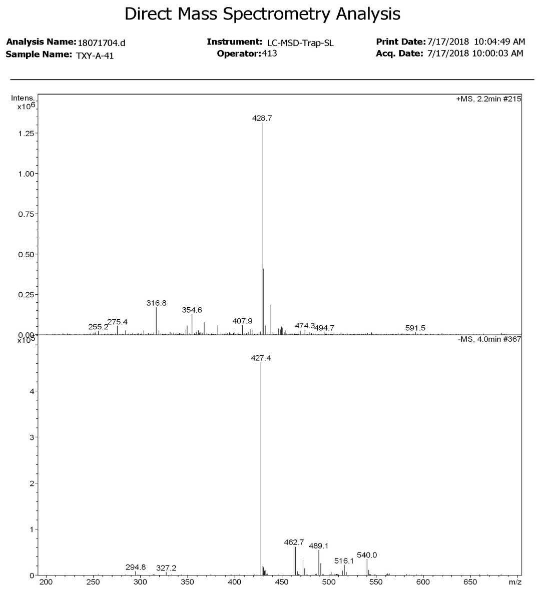

Figure S59. ESI-MS of the target compound 7l

Mass Spectrum SmartFormula Report

Analysis Info

Analysis Name	D:\data\20190521\ZCM-7l pos_000001.d	Acquisition Date	5/21/2019 4:59:37 PM
Method	120-1200_pos_20190520	Operator	
Sample Name		Instrument	solariX
Comment			

Acquisition Parameter

Acquisition Mode	Single MS	Acquired Scans	2	Calibration Date	Mon May 20 10:10:48
Polarity	Positive	No. of Cell Fills	1	Data Acquisition Size	1048576
Broadband Low Mass	118.2 m/z	No. of Laser Shots	500	Data Processing Size (SI)	2097152
Broadband High Mass	1200.0 m/z	Laser Power	20.0 lp	Apodization	Full-Sine
Source Accumulation	0.000 sec	Laser Shot Frequency	0.001 sec		
Ion Accumulation Time	0.050 sec				

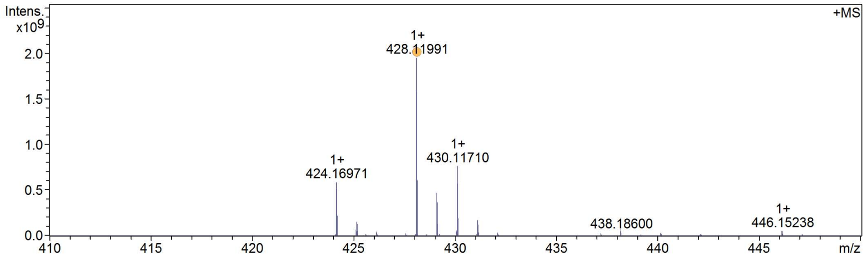

Meas. m/z	#	Ion Formula	Score	m/z	err [ppm]
428.119913	1	C22H23ClN3O2S	100.00	428.119402	-1.2

Figure S60. HRMS of the target compound 7l

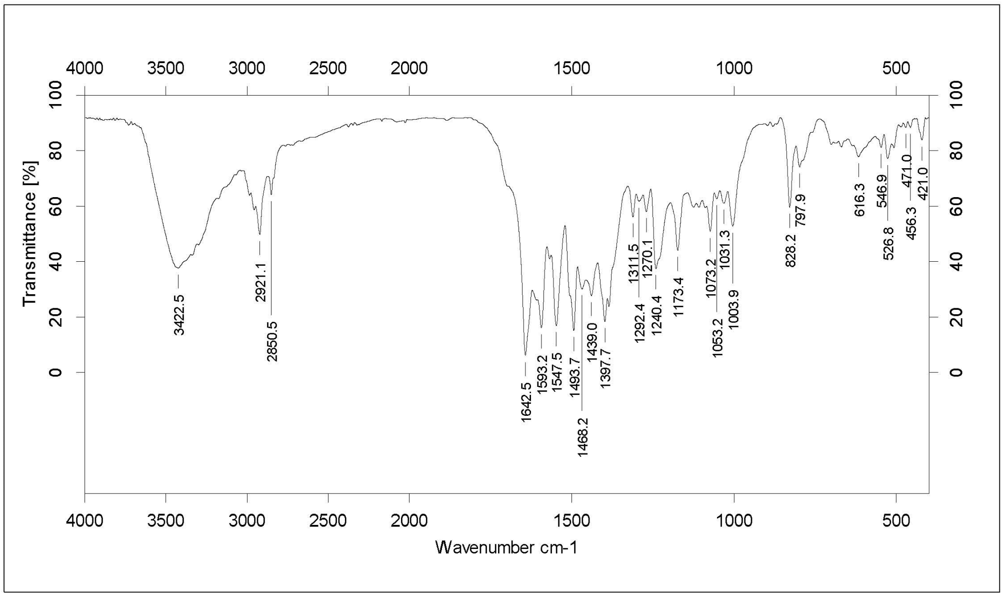

Sample : ZCM-30	Frequency Range : 3999.64 - 400.157	Measured on : 2019-4-12	
Technique : Sample form	Resolution : 2	Instrument : EQUINOX55	Sample Scans : 16
Customer : Default	Zerofilling : 2	Acquisition : Double Sided,Forv	

Figure S61. IR of the target compound 7m

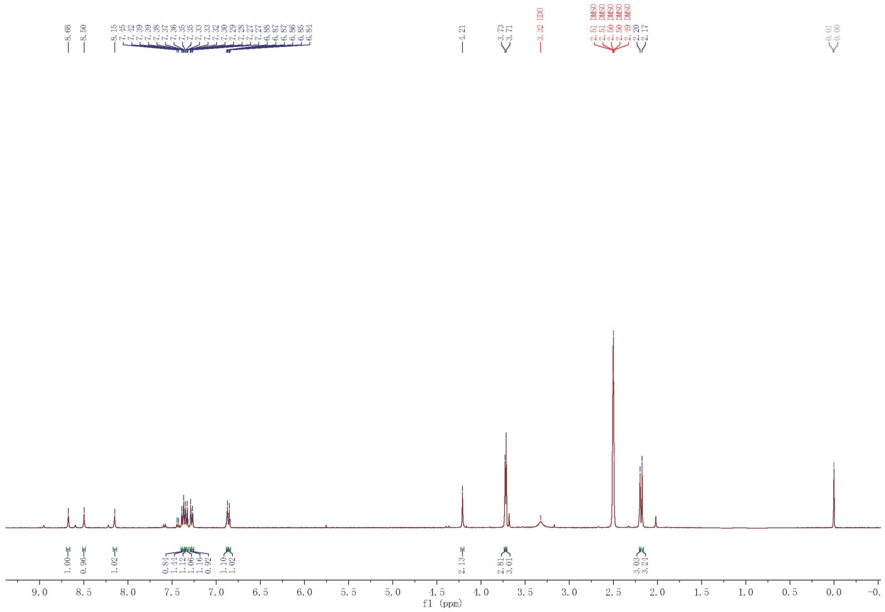

Figure S62. ^{1}H-NMR spectra of the target compound 7m

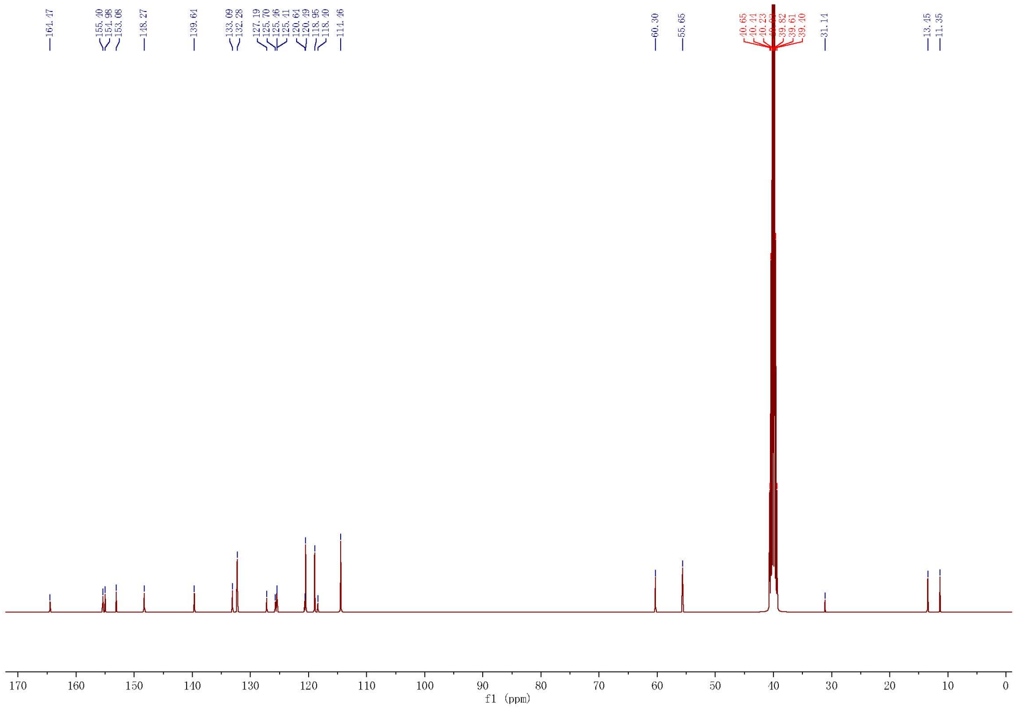

Figure S63. ^{13}C-NMR spectra of the target compound 7m

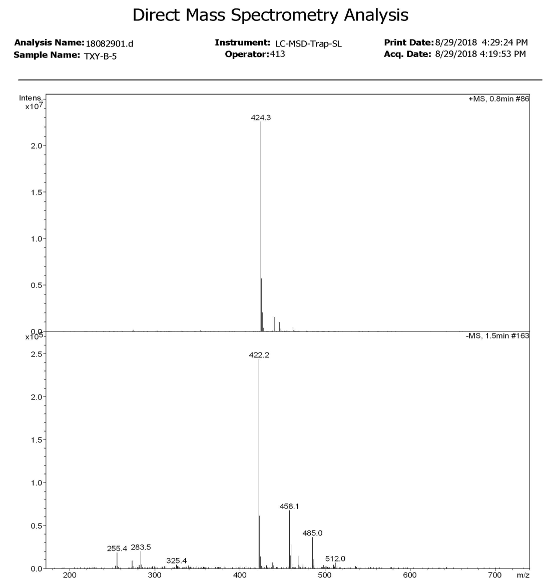

Figure S64. ESI-MS of the target compound 7m

Mass Spectrum SmartFormula Report

Analysis Info

Analysis Name	D:\data\20190521\ZCM-7m pos_000003.d	Acquisition Date	5/21/2019 4:49:02 PM
Method	120-1200_pos_20190520	Operator	
Sample Name		Instrument	solariX
Comment			

Acquisition Parameter

Acquisition Mode	Single MS	Acquired Scans	2	Calibration Date	Mon May 20 10:10:48
Polarity	Positive	No. of Cell Fills	1	Data Acquisition Size	1048576
Broadband Low Mass	118.2 m/z	No. of Laser Shots	500	Data Processing Size (SI)	2097152
Broadband High Mass	1200.0 m/z	Laser Power	20.0 lp	Apodization	Full-Sine
Source Accumulation	0.000 sec	Laser Shot Frequency	0.001 sec		
Ion Accumulation Time	0.050 sec				

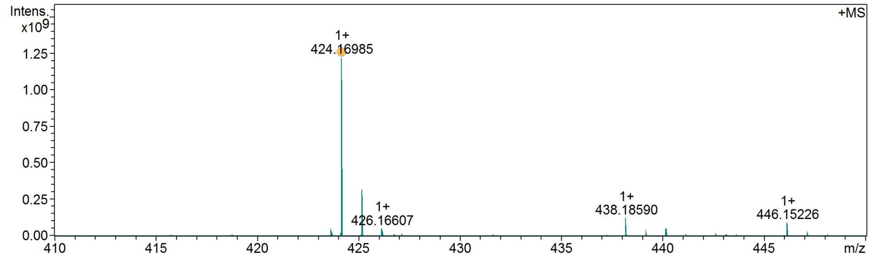

Meas. m/z	#	Ion Formula	Score	m/z	err [ppm]
424.169851	1	C23H26N3O3S	100.00	424.168939	-2.1

Bruker Compass DataAnalysis 4.4 By: Shenyang Pharmaceutical University Page 1 of 1

Figure S65. HRMS of the target compound 7m

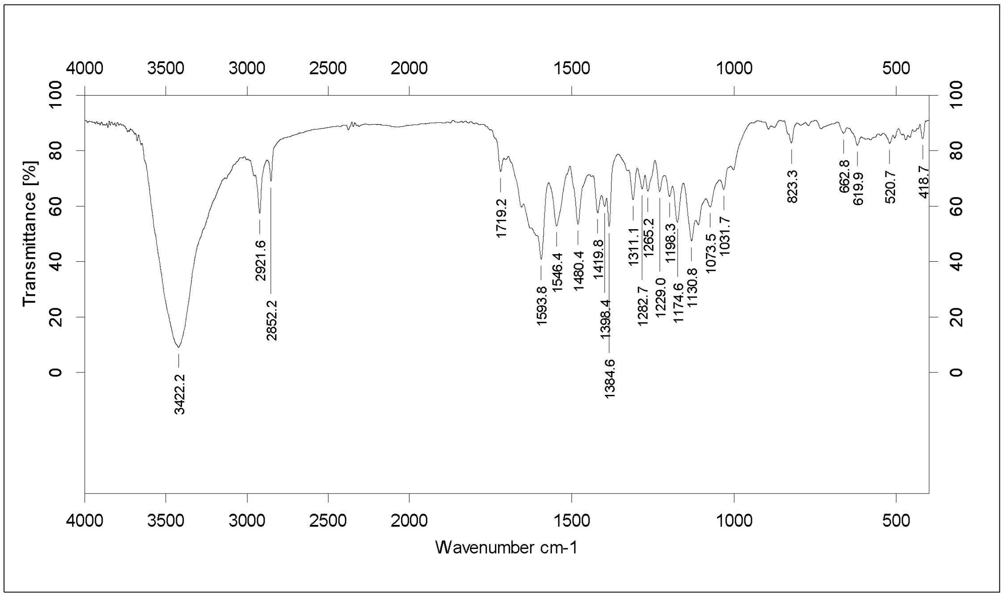

Sample : ZCM-24	Frequency Range : 3999.64 - 400.157	Measured on : 2019-4-25	
Technique : Sample form	Resolution : 2	Instrument : EQUINOX55	Sample Scans : 16
Customer : Default	Zerofilling : 2	Acquisition : Double Sided,Forv	

Figure S66. IR of the target compound 7n

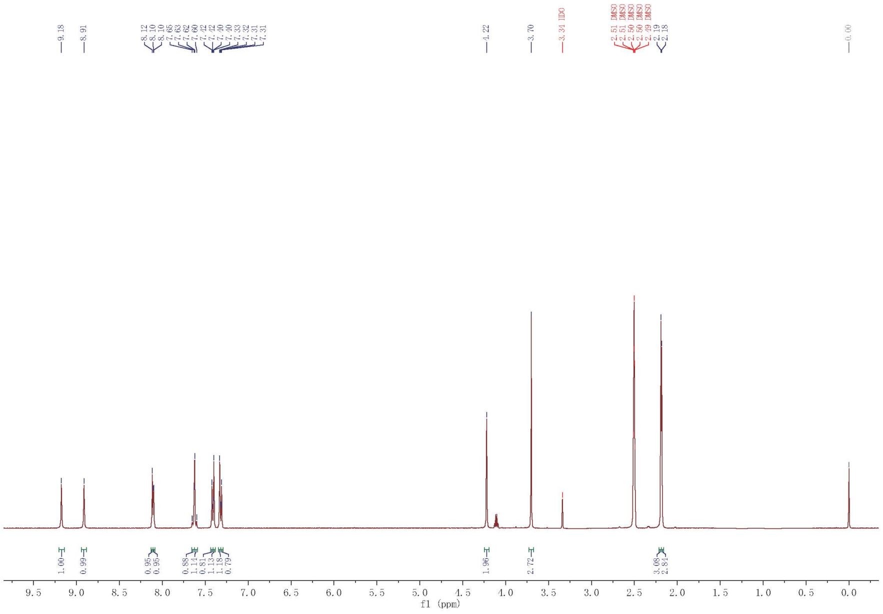

Figure S67. [1] H-NMR spectra of the target compound 7n

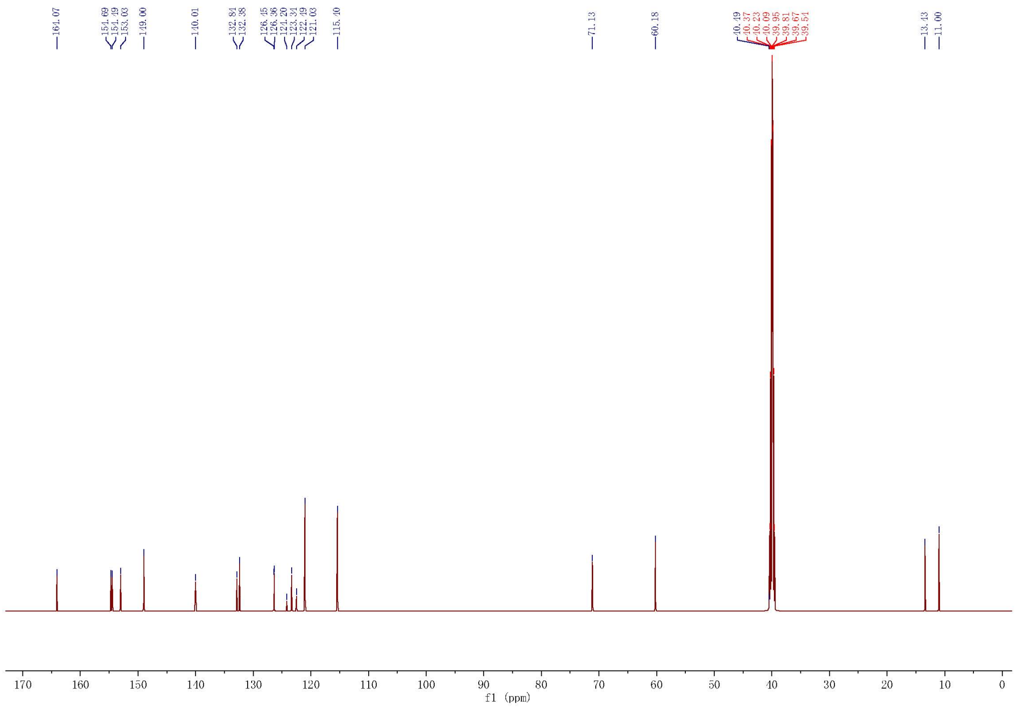

Figure S68. ^{13}C-NMR spectra of the target compound 7n

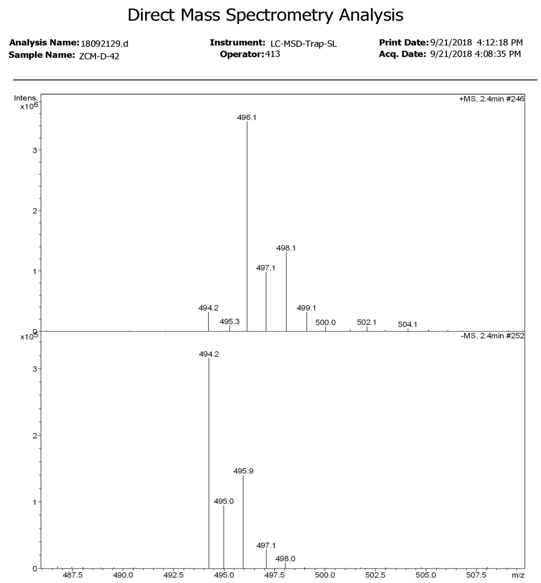

Figure S69. ESI-MS of the target compound 7n

Qualitative Analysis Report

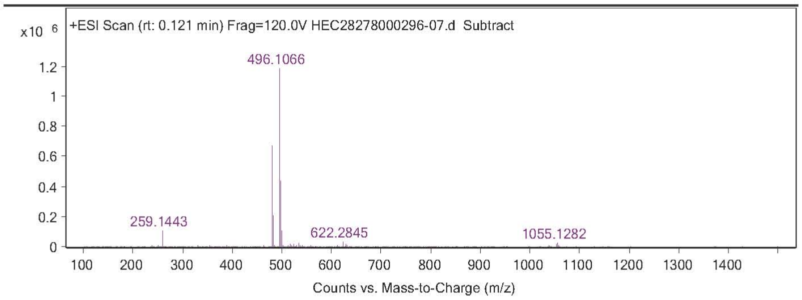

Peak List

m/z	z	Abund
259.1443	1	100371.26
480.1293	1	670731.94
481.1334	1	156642
482.1281	1	206422.86
483.1308	1	49991.47
496.1066	1	1184339.25
497.1102	1	299465.56
498.1048	1	435739.75
499.1076	1	106051.77
622.2845	1	28392.69

--- End Of Report ---

Agilent Technologies Page 2 of 2 Printed at 12:49 PM on 2-Mar-2019

Figure S70. HRMS of the target compound 7n

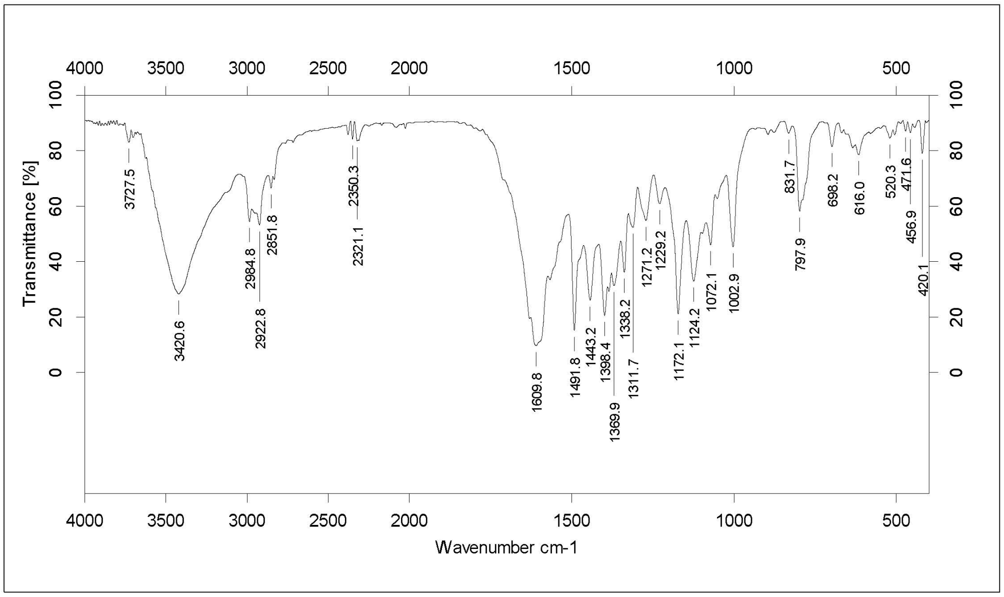

Sample : ZCM-28	Frequency Range : 3999.64 - 400.157	Measured on : 2019-4-11	
Technique : Sample form	Resolution : 2	Instrument : EQUINOX55	Sample Scans : 16
Customer : Default	Zerofilling : 2	Acquisition : Double Sided,Forv	

Figure S71. IR of the target compound 7o

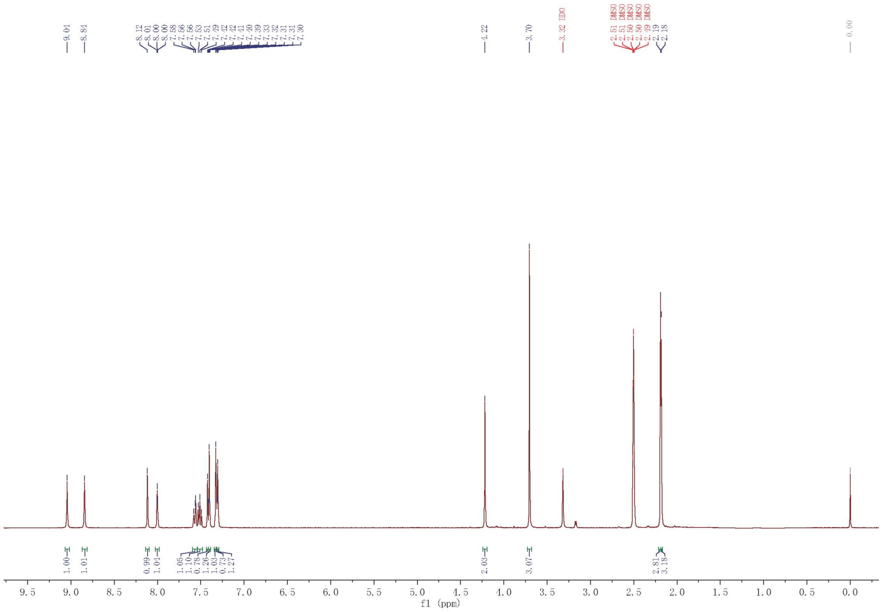

Figure S72. ^{1}H-NMR spectra of the target compound 7o

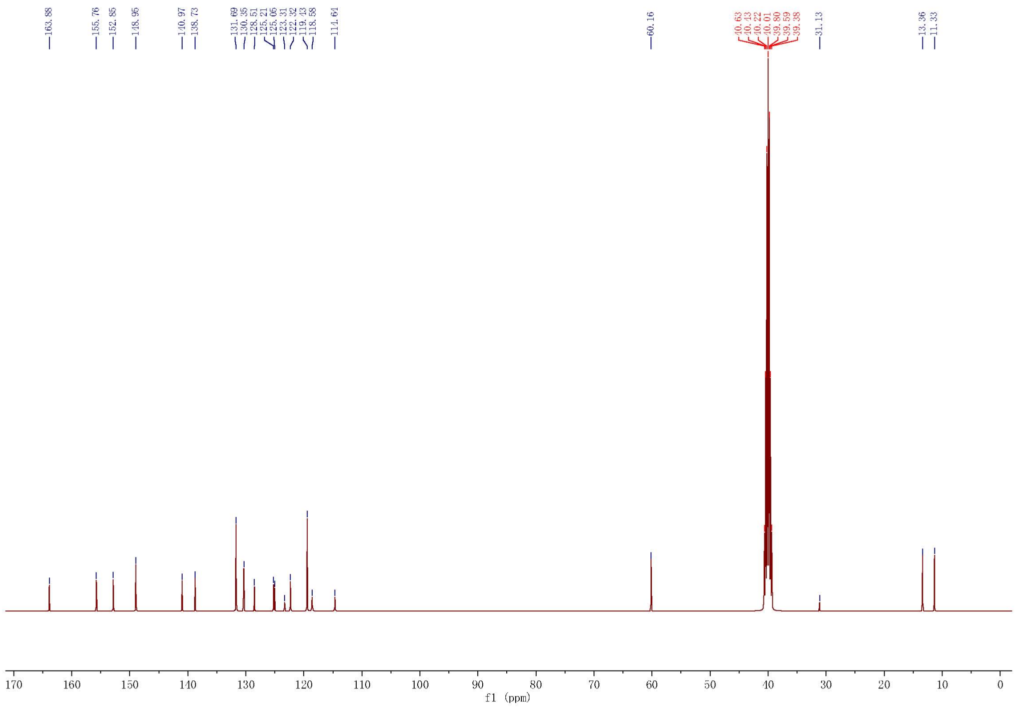

Figure S73. ^{13}C-NMR spectra of the target compound 7o

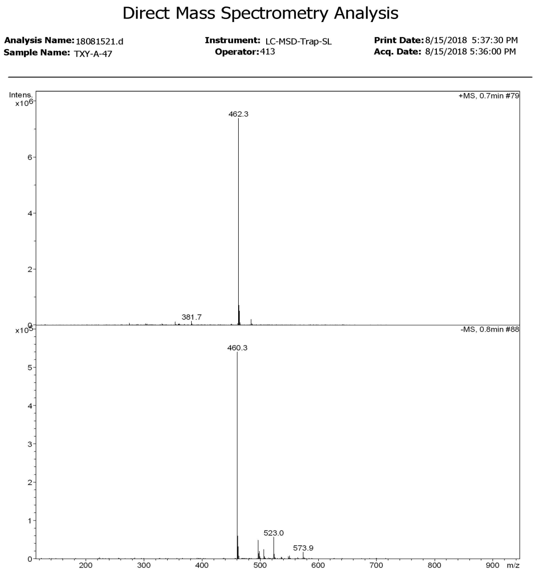

Figure S74. ESI-MS of the target compound 7o

Mass Spectrum SmartFormula Report

Analysis Info

Analysis Name	D:\data\20190521\ZCM-7o pos_000001.d	Acquisition Date	5/21/2019 4:32:42 PM
Method	120-1200_pos_20190520	Operator	
Sample Name		Instrument	solariX
Comment			

Acquisition Parameter

Acquisition Mode	Single MS	Acquired Scans	3	Calibration Date	Mon May 20 10:10:48
Polarity	Positive	No. of Cell Fills	1	Data Acquisition Size	1048576
Broadband Low Mass	118.2 m/z	No. of Laser Shots	500	Data Processing Size (SI)	2097152
Broadband High Mass	1200.0 m/z	Laser Power	20.0 lp	Apodization	Full-Sine
Source Accumulation	0.000 sec	Laser Shot Frequency	0.001 sec		
Ion Accumulation Time	0.050 sec				

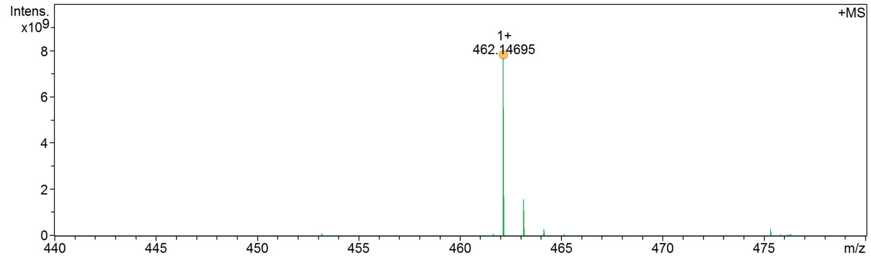

Meas. m/z	#	Ion Formula	Score	m/z	err [ppm]
462.146953	1	C23H23F3N3O2S	100.00	462.145759	-2.6

Figure S75. HRMS of the target compound 7o

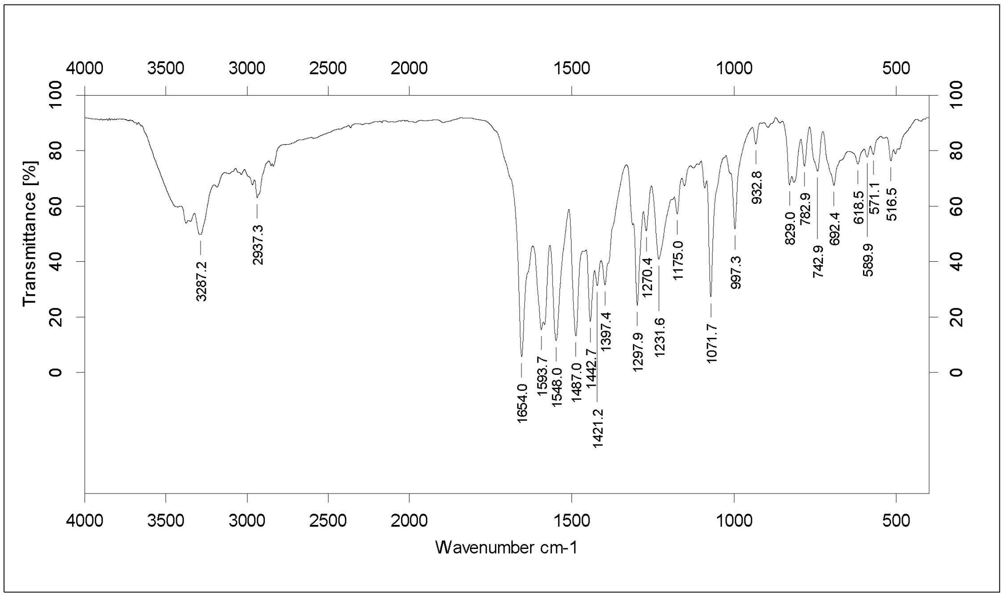

Sample : ZCM-15	Frequency Range : 3999.64 - 400.157	Measured on : 2019-4-12	
Technique : Sample form	Resolution : 2	Instrument : EQUINOX55	Sample Scans : 16
Customer : Default	Zerofilling : 2	Acquisition : Double Sided,Forv	

Figure S76. IR of the target compound 7p

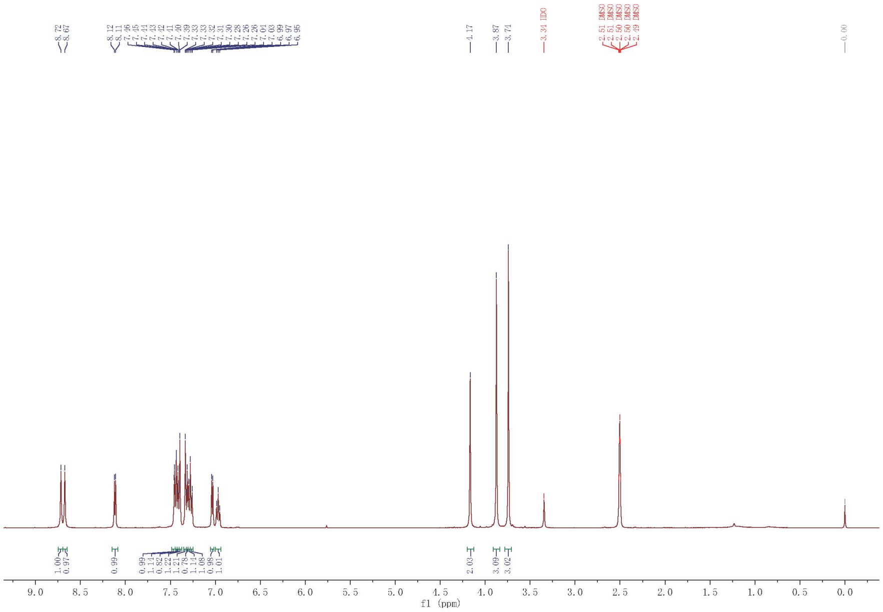

Figure S77. ^{1}H-NMR spectra of the target compound 7p

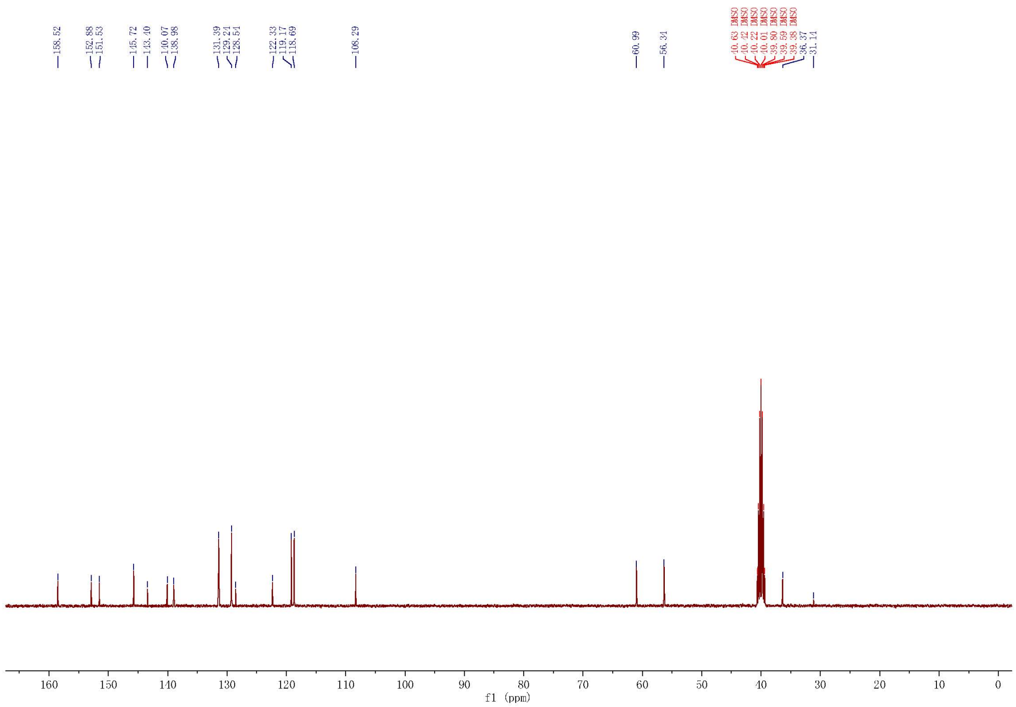

Figure S78. ^{13}C-NMR spectra of the target compound 7p

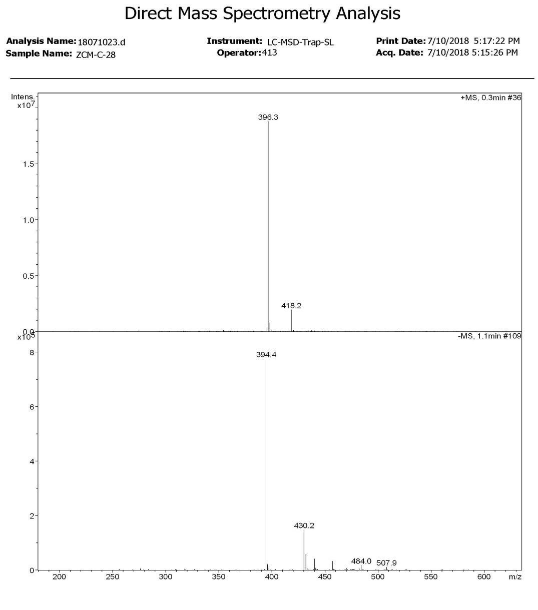

Figure S79. ESI-MS of the target compound 7p

Mass Spectrum SmartFormula Report

Analysis Info

Analysis Name	D:\data\20190521\ZCM-7p pos_000001.d	Acquisition Date	5/21/2019 5:59:51 PM
Method	120-1200_pos_20190520	Operator	
Sample Name		Instrument	solariX
Comment			

Acquisition Parameter

Acquisition Mode	Single MS	Acquired Scans	2	Calibration Date	Mon May 20 10:10:48
Polarity	Positive	No. of Cell Fills	1	Data Acquisition Size	1048576
Broadband Low Mass	118.2 m/z	No. of Laser Shots	500	Data Processing Size (SI)	2097152
Broadband High Mass	1200.0 m/z	Laser Power	20.0 lp	Apodization	Full-Sine
Source Accumulation	0.000 sec	Laser Shot Frequency	0.001 sec		
Ion Accumulation Time	0.050 sec				

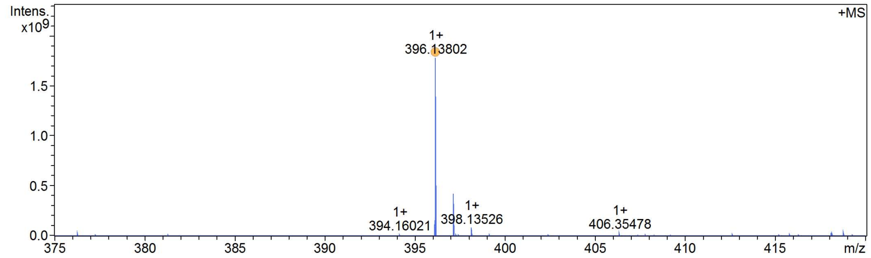

Meas. m/z	#	Ion Formula	Score	m/z	err [ppm]
396.138016	1	C21H22N3O3S	100.00	396.137639	-1.0

Bruker Compass DataAnalysis 4.4 By: Shenyang Pharmaceutical University Page 1 of 1

Figure S80. HRMS of the target compound 7p

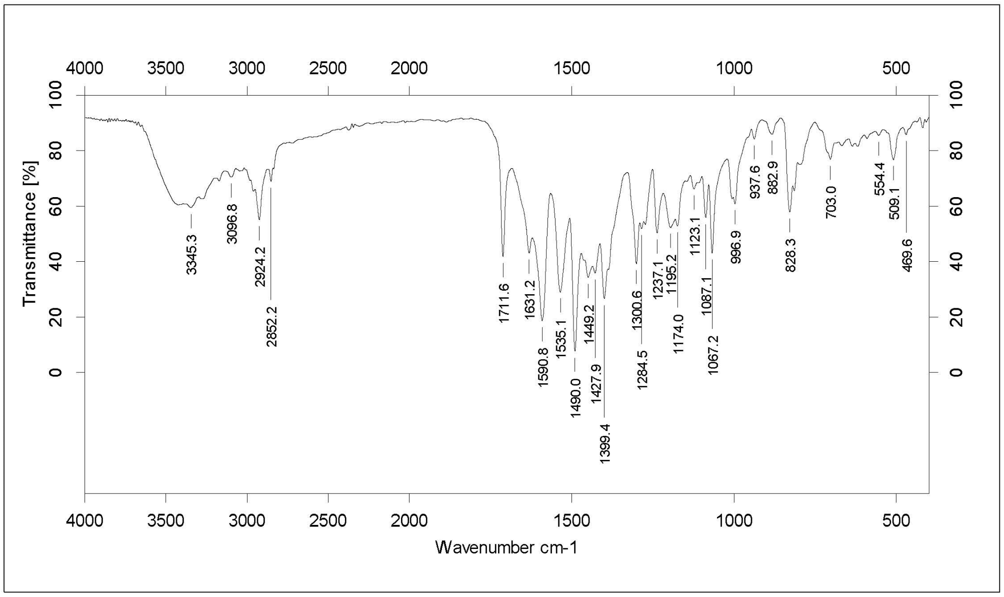

Sample : ZCM-16	Frequency Range : 3999.64 - 400.157		Measured on : 2019-4-12
Technique : Sample form	Resolution : 2	Instrument : EQUINOX55	Sample Scans : 16
Customer : Default	Zerofilling : 2	Acquisition : Double Sided,Forv	

Figure S81. IR of the target compound 7q

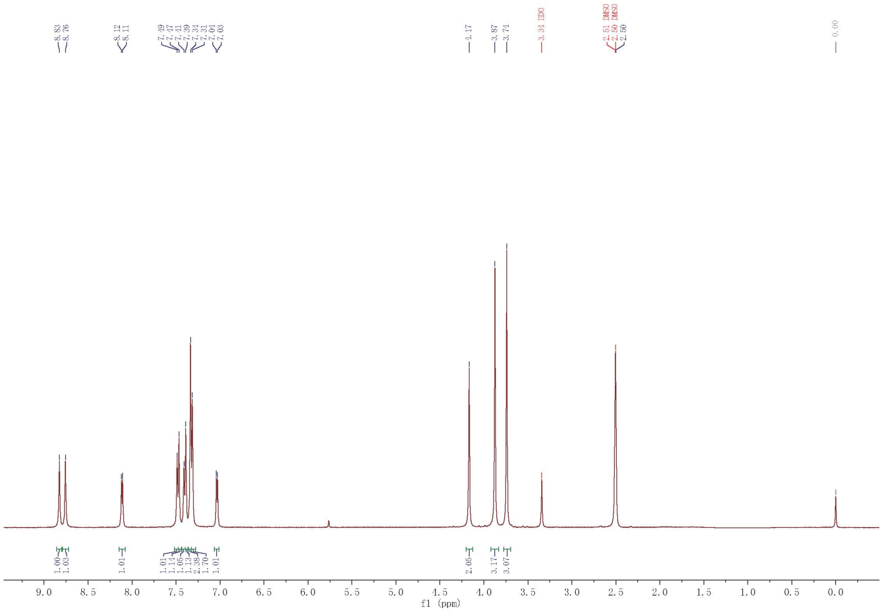

Figure S82. ^{1}H-NMR spectra of the target compound 7q

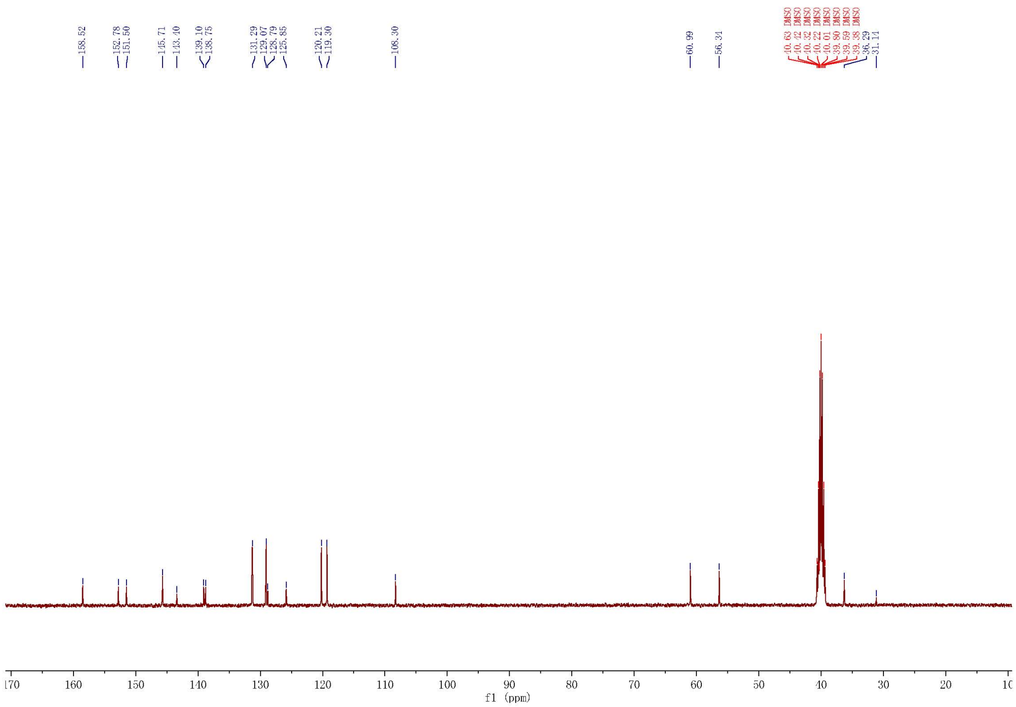

Figure S83. ^{13}C-NMR spectra of the target compound 7q

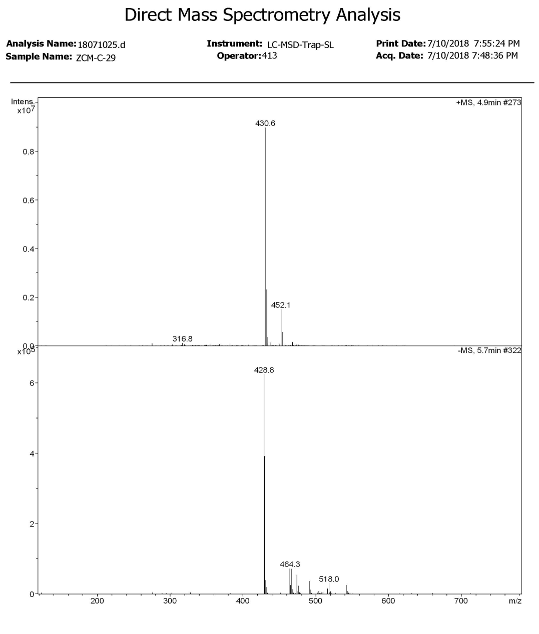

Figure S84. ESI-MS of the target compound 7q

Mass Spectrum SmartFormula Report

Analysis Info

Analysis Name	D:\data\20190521\ZCM-7q pos_000005.d	Acquisition Date	5/21/2019 5:53:06 PM
Method	120-1200_pos_20190520	Operator	
Sample Name		Instrument	solariX
Comment			

Acquisition Parameter

Acquisition Mode	Single MS	Acquired Scans	2	Calibration Date	Mon May 20 10:10:48
Polarity	Positive	No. of Cell Fills	1	Data Acquisition Size	[illegible]576
Broadband Low Mass	118.2 m/z	No. of Laser Shots	500	Data Processing Size (SI)	2097152
Broadband High Mass	1200.0 m/z	Laser Power	20.0 lp	Apodization	Full-Sine
Source Accumulation	0.000 sec	Laser Shot Frequency	0.001 sec		
Ion Accumulation Time	0.050 sec				

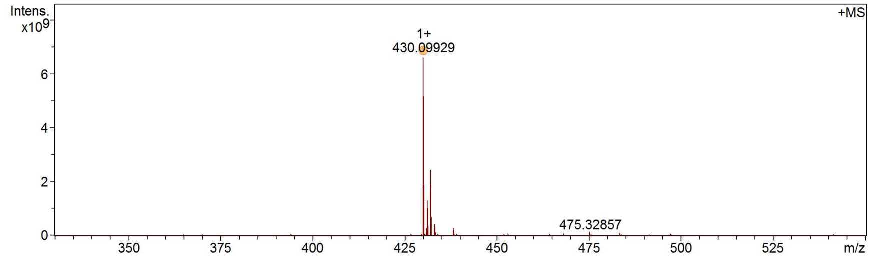

Meas. m/z	#	Ion Formula	Score	m/z	err [ppm]
430.099000	1	C21H21ClN3O3S	n.a.	430.098667	-0.8

Figure S85. HRMS of the target compound 7q

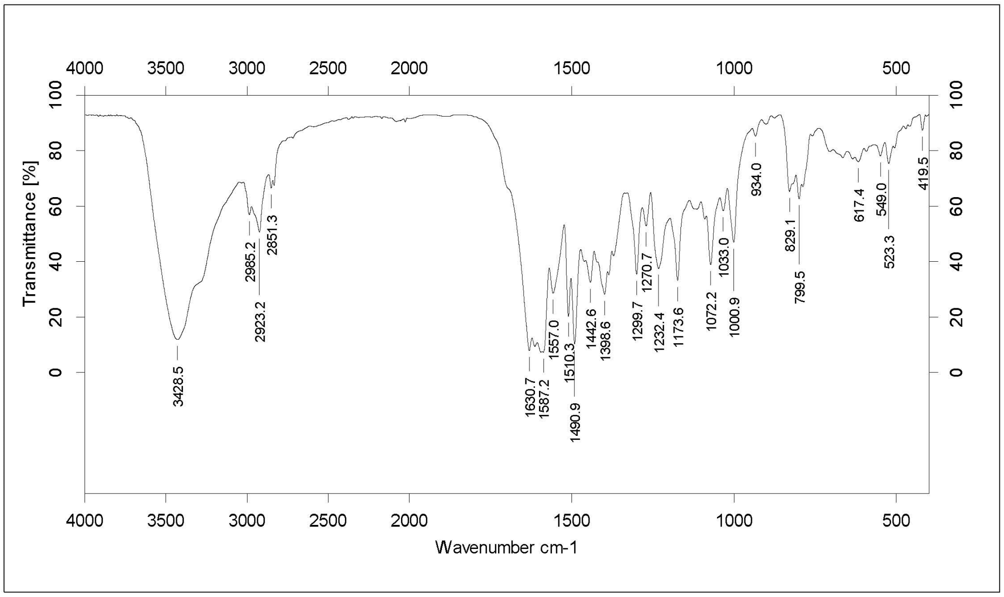

Sample : ZCM-23		Frequency Range : 3999.64 - 400.157	Measured on : 2019-4-12
Technique : Sample form	Resolution : 2	Instrument : EQUINOX55	Sample Scans : 16
Customer : Default	Zerofilling : 2	Acquisition : Double Sided,Forv	

Figure S86. IR of the target compound 7r

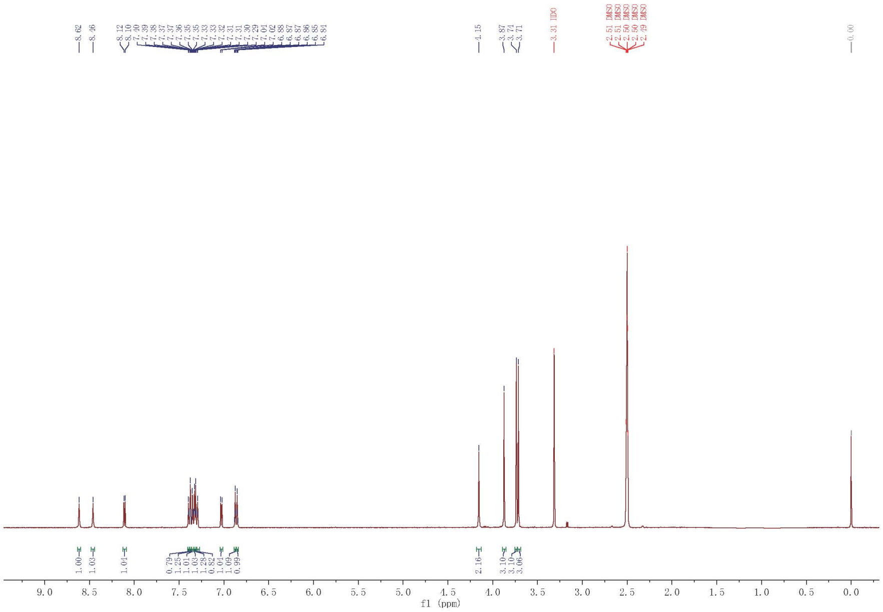

Figure S87. [1] H-NMR spectra of the target compound 7r

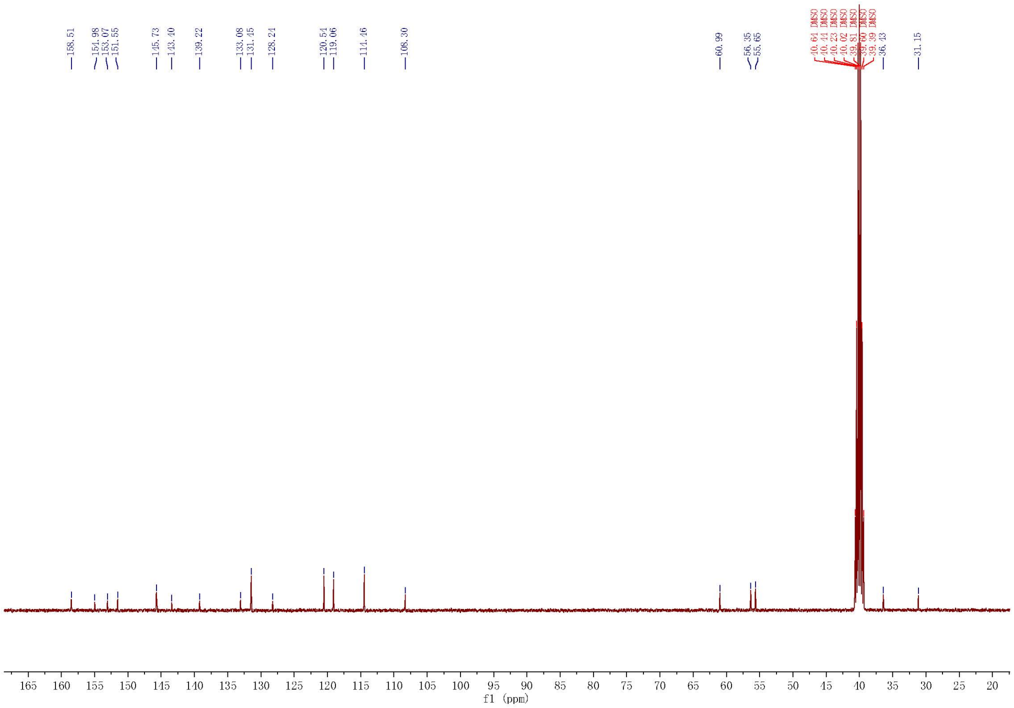

Figure S88. ^{13}C-NMR spectra of the target compound 7r

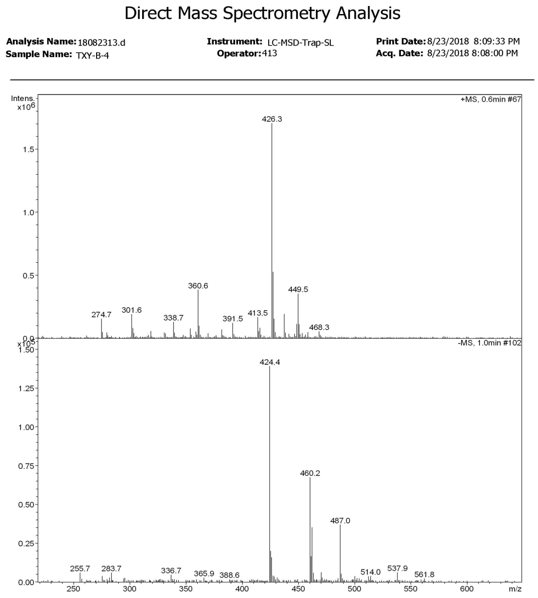

Figure S89. ESI-MS of the target compound 7r

Mass Spectrum SmartFormula Report

Analysis Info

Analysis Name	D:\data\20190521\ZCM-7r pos_000001.d	Acquisition Date	5/21/2019 4:22:23 PM
Method	120-1200_pos_20190520	Operator	
Sample Name		Instrument	solariX
Comment			

Acquisition Parameter

Acquisition Mode	Single MS	Acquired Scans	3	Calibration Date	Mon May 20 10:10:48
Polarity	Positive	No. of Cell Fills	1	Data Acquisition Size	[illegible]
Broadband Low Mass	118.2 m/z	No. of Laser Shots	500	Data Processing Size (SI)	2097152
Broadband High Mass	1200.0 m/z	Laser Power	20.0 lp	Apodization	Full-Sine
Source Accumulation	0.000 sec	Laser Shot Frequency	0.001 sec		
Ion Accumulation Time	0.050 sec				

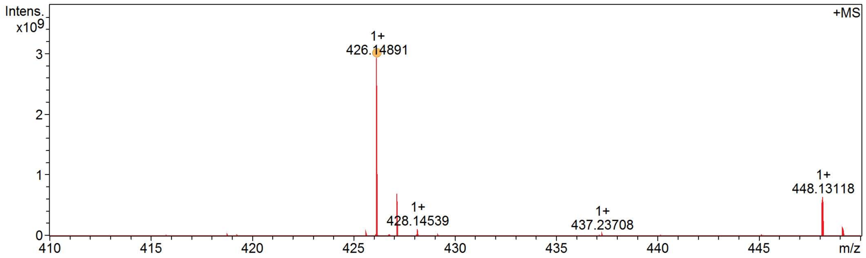

Meas. m/z	#	Ion Formula	Score	m/z	err [ppm]
426.148909	1	C22H24N3O4S	11.71	426.148204	-1.7

Bruker Compass DataAnalysis 4.4 By: Shenyang Pharmaceutical University Page 1 of 1

Figure S90. HRMS of the target compound 7r

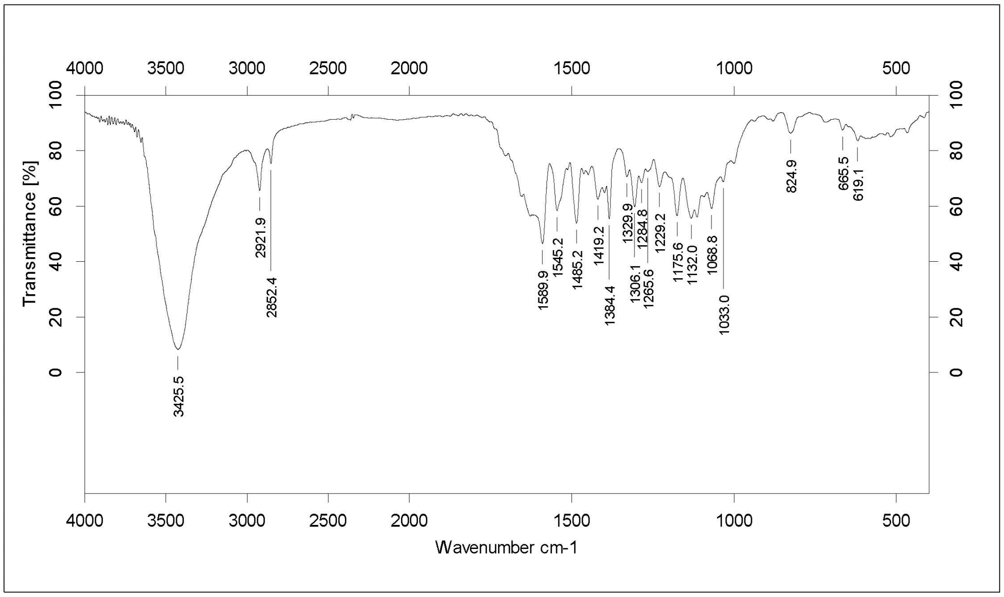

Sample : ZCM-18	Frequency Range : 3999.64 - 400.157	Measured on : 2019-4-25	
Technique : Sample form	Resolution : 2	Instrument : EQUINOX55	Sample Scans : 16
Customer : Default	Zerofilling : 2	Acquisition : Double Sided,Forv	

Figure S91. IR of the target compound 7s

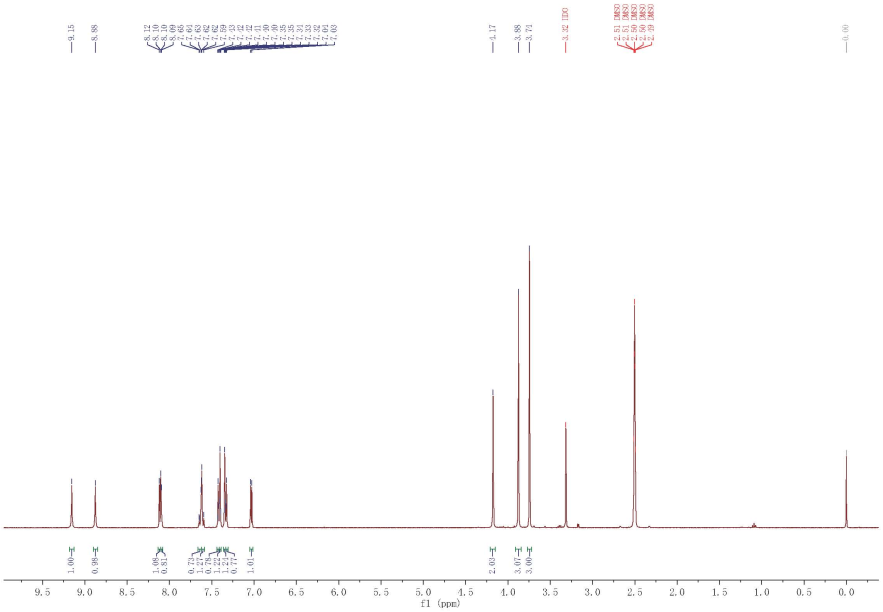

Figure S92. ^{1}H-NMR spectra of the target compound 7s

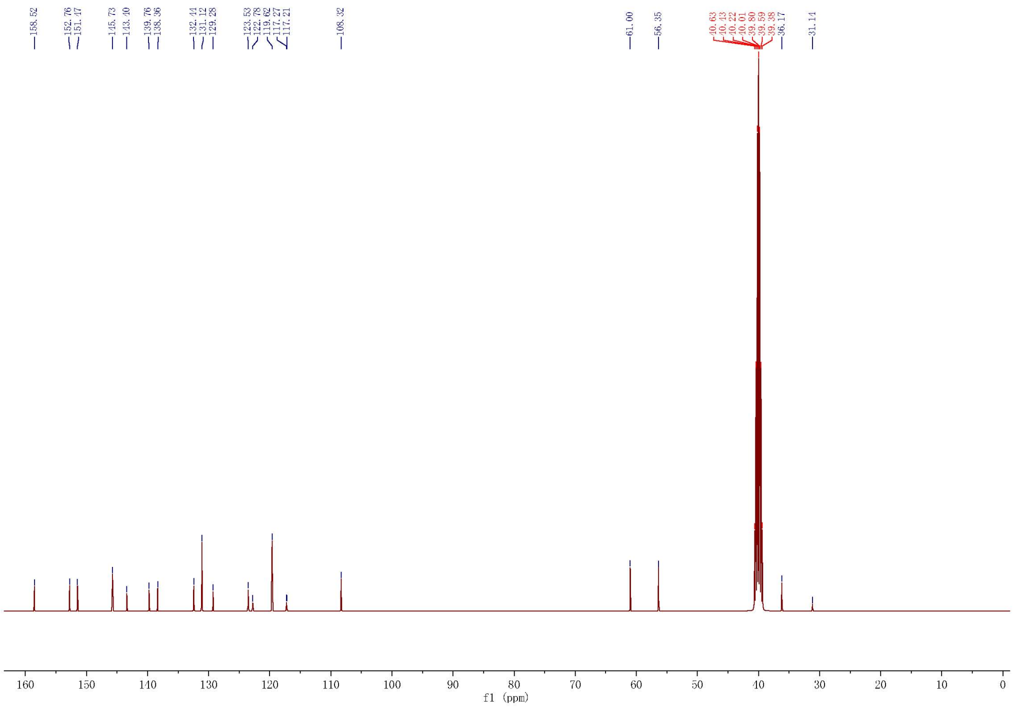

Figure S93. ^{13}C-NMR spectra of the target compound 7s

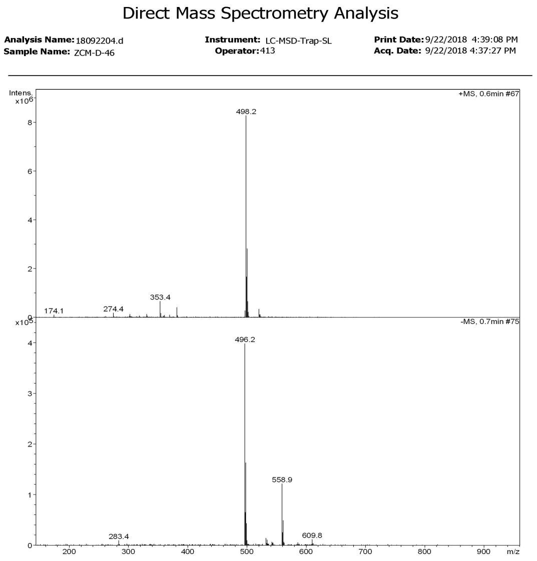

Figure S94. ESI-MS of the target compound 7s

Qualitative Analysis Report

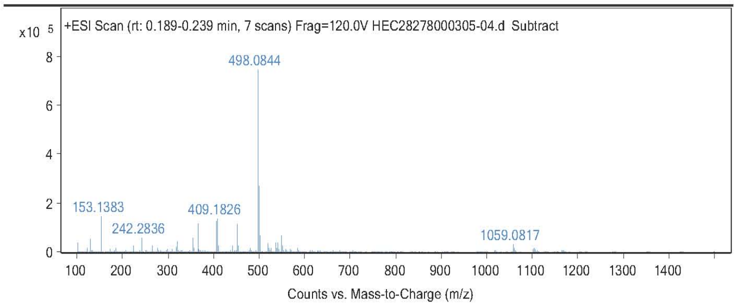

Peak List

m/z	z	Abund
130.1587		52332.19
153.1383	1	145043.88
242.2836	1	55239.47
321.1303		43142.63
355.3671	1	53752.88
365.1565	1	114514.13
407.326	1	123190.45
409.1826	1	133855.06
453.2087	1	111088.47
497.2347		72172.86
498.0844	1	743363.94
499.0881	1	176050.73
500.0828	1	269775
501.0851	1	66460.7
550.0769	1	65974.03

--- End Of Report ---

Agilent Technologies Page 2 of 2 Printed at 8:54 AM on 11-Mar-2019

Figure S95. HRMS of the target compound 7s

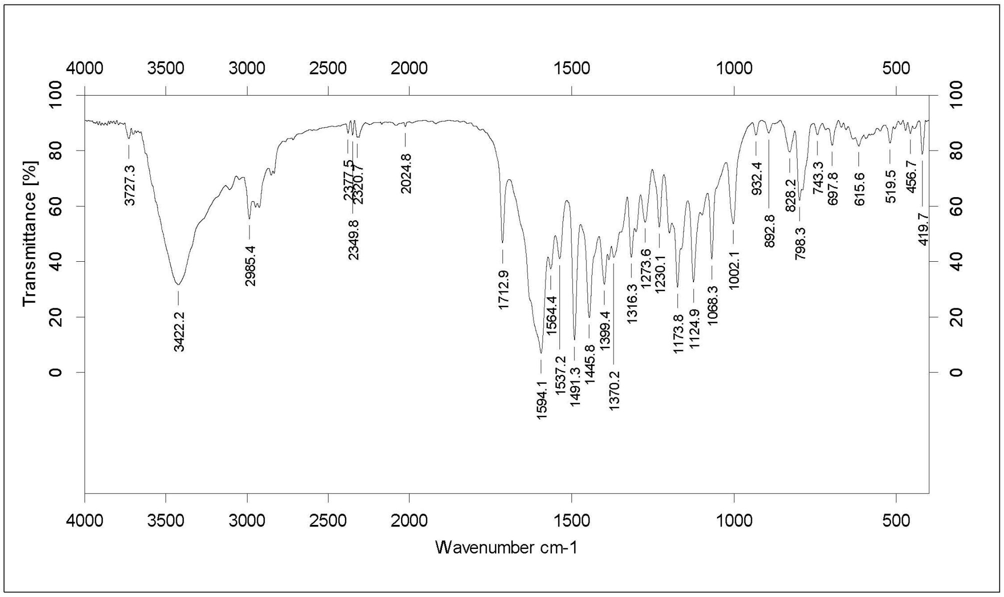

Sample : ZCM-21		Frequency Range : 3999.64 - 400.157	Measured on : 2019-4-11
Technique : Sample form	Resolution : 2	Instrument : EQUINOX55	Sample Scans : 16
Customer : Default	Zerofilling : 2	Acquisition : Double Sided,Forv	

Figure S96. IR of the target compound 7t

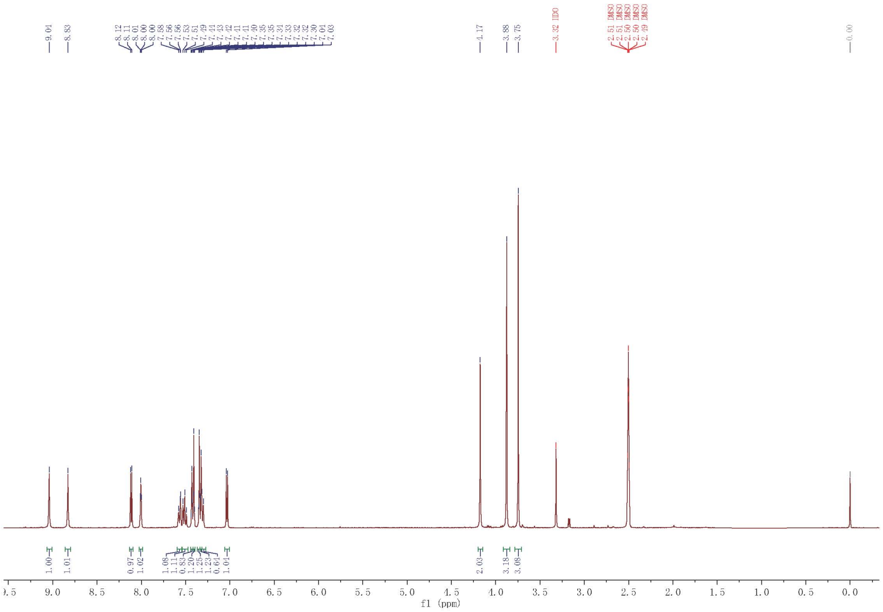

Figure S97. [1] H-NMR spectra of the target compound 7t

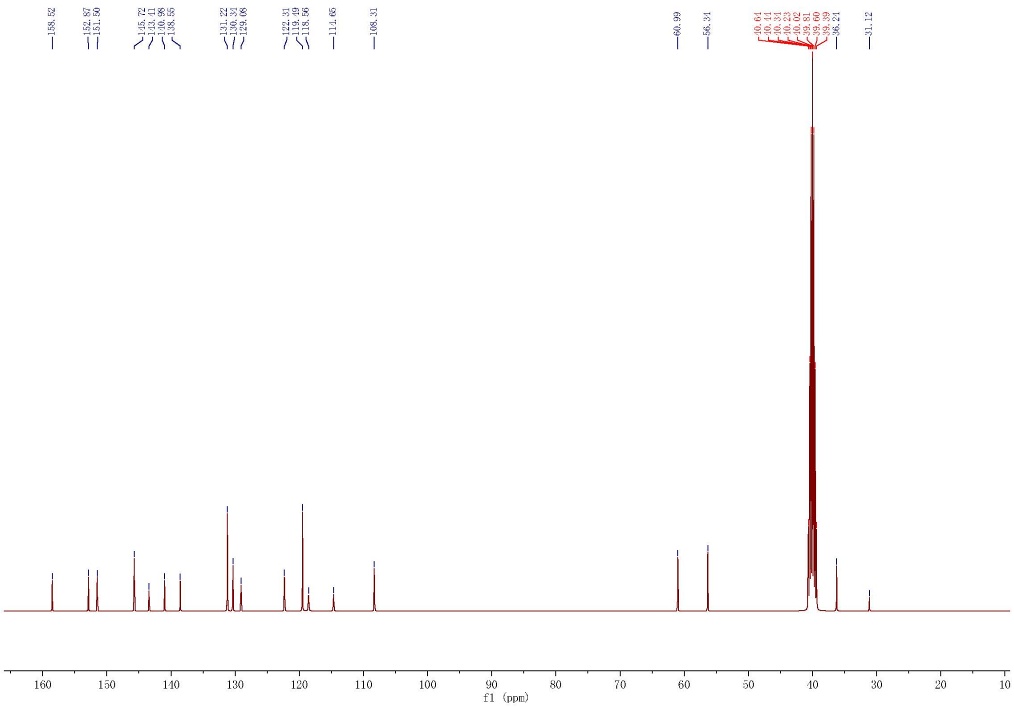

Figure S98. ^{13}C-NMR spectra of the target compound 7t

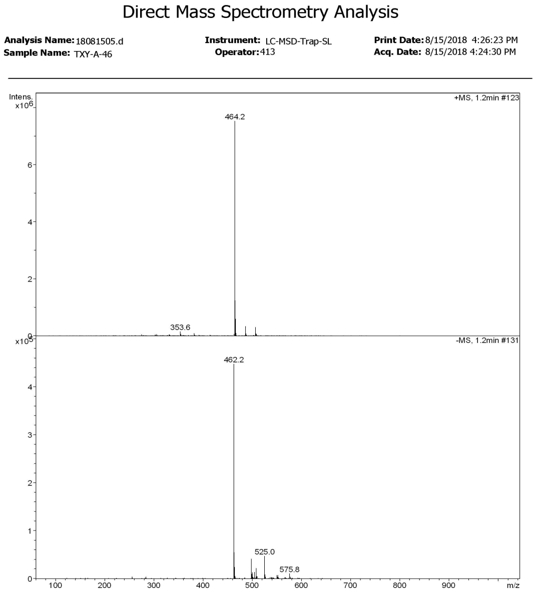

Figure S99. ESI-MS of the target compound 7t
